# Host ZCCHC3 blocks HIV-1 infection and production through a dual mechanism

**DOI:** 10.1016/j.isci.2024.109107

**Published:** 2024-02-05

**Authors:** Binbin Yi, Yuri L. Tanaka, Daphne Cornish, Hidetaka Kosako, Erika P. Butlertanaka, Prabuddha Sengupta, Jennifer Lippincott-Schwartz, Judd F. Hultquist, Akatsuki Saito, Shige H. Yoshimura

**Affiliations:** 1 Graduate School of Biostudies, Kyoto University, Yoshida-Konoe-Cho, Sakyo-ku, Kyoto 606-8501, Japan; 2 Department of Veterinary Medicine, Faculty of Agriculture, University of Miyazaki, 1-1 Gakuen Kibanadai-nishi, Miyazaki, Miyazaki 889-2192, Japan; 3 Division of Infectious Diseases, Northwestern University Feinberg School of Medicine, Chicago, IL 60611, USA; 4 Center for Pathogen Genomics and Microbial Evolution, Northwestern University Havey Institute for Global Health, Chicago, IL 60611, USA; 5 Division of Cell Signaling, Fujii Memorial Institute of Medical Sciences, Institute of Advanced Medical Sciences, Tokushima University, 3-18-15 Kuramoto-cho, Tokushima 770-8503, Japan; 6 Howard Hughes Medical Institute, Janelia Research Campus, 19700 Helix Drive, Ashburn, VA 20147, USA; 7 Center for Animal Disease Control, University of Miyazaki, 1-1 Gakuen Kibanadai-nishi, Miyazaki, Miyazaki 889-2192, Japan; 8 Graduate School of Medicine and Veterinary Medicine, University of Miyazaki, 5200 Kiyotakecho Kihara, Miyazaki, Miyazaki 889-1692, Japan; 9 Center for Living Systems Information Science (CeLiSIS), Kyoto University, Yoshida-Konoe-Cho, Sakyo-ku, Kyoto 606-8501, Japan

**Keywords:** Immunology, Virology, Biological sciences

## Abstract

Most mammalian cells prevent viral infection and proliferation by expressing various restriction factors and sensors that activate the immune system. Several host restriction factors that inhibit human immunodeficiency virus type 1 (HIV-1) have been identified, but most of them are antagonized by viral proteins. Here, we describe CCHC-type zinc-finger-containing protein 3 (ZCCHC3) as a novel HIV-1 restriction factor that suppresses the production of HIV-1 and other retroviruses, but does not appear to be directly antagonized by viral proteins. It acts by binding to Gag nucleocapsid (GagNC) via zinc-finger motifs, which inhibits viral genome recruitment and results in genome-deficient virion production. ZCCHC3 also binds to the long terminal repeat on the viral genome via the middle-folded domain, sequestering the viral genome to P-bodies, which leads to decreased viral replication and production. This distinct, dual-acting antiviral mechanism makes upregulation of ZCCHC3 a novel potential therapeutic strategy.

## Highlights

• ZCCHC3 is a host restriction factor for HIV-1
• ZCCHC3 suppresses HIV-1 production by binding to the Gag nucleocapsid
• ZCCHC3 binds to LTR of HIV-1 genomic RNA and sequesters it to P-body

## Graphical abstract

**Figure:**
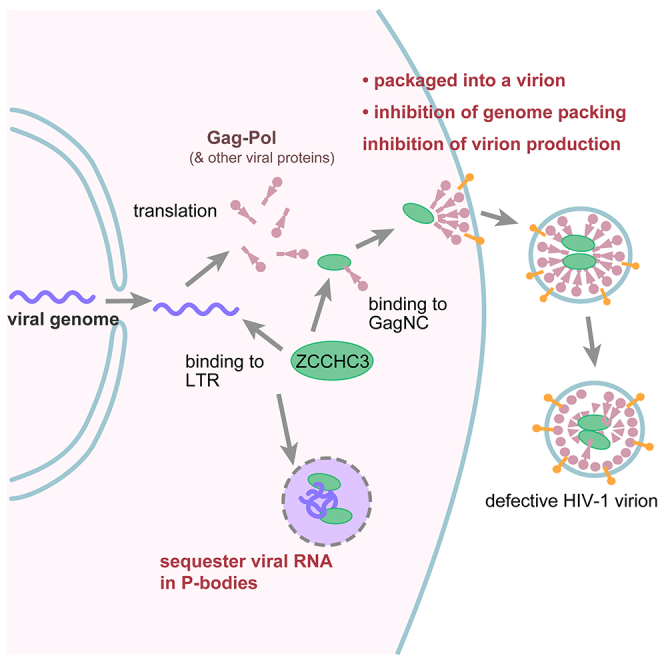

## Introduction

Human immunodeficiency virus type 1 (HIV-1) infection is a major global health concern, affecting tens of millions of individuals worldwide. The HIV-1 life cycle relies on a large number of host proteins that promote efficient progress of viral replication, packaging, and budding. As a defense strategy, host cells produce a number of restriction factors, i.e., proteins that prevent a specific step in the viral life cycle or recognize specific viral components and activate the immune system.^1^^,^^2^ Several anti-HIV-1 restriction factors have been identified with diverse mechanisms involving direct interaction with, modification of, and/or blocking of specific viral structures.^3^ Several restriction factors target the viral genome. For example, the APOBEC3^4^^,^^5^^,^^6^^,^^7^ family of proteins inhibits viral proliferation by introducing nucleotide mutations in the viral genome during its reverse transcription.^8^^,^^9^ ZAP1^10^^,^^11^ recognizes single-stranded CG dinucleotide-rich regions of HIV-1 RNA^12^^,^^13^ and recruits a ribonuclease and RNA exosome to degrade it. As another example, the nuclear exosome-targeting (NEXT) complex recognizes the transactivation response (TAR) element on the HIV-1 genome and inhibits transcription.^14^ Several other host factors inhibit critical steps in the life cycle without directly targeting the genome itself. For instance, tetherin anchors budding HIV-1 particles to the host plasma membrane and prevents the release of the virion into the extracellular space.^15^^,^^16^ SAMHD1 prevents the reverse transcription of HIV-1 by depleting the intracellular dNTP pool.^17^^,^^18^ To counteract these restriction factors, HIV-1 encodes viral accessory proteins that relocalize and/or degrade these antiviral proteins. For example, the HIV-1 protein Vif antagonizes the antiviral activity of APOBEC3,^6^ and the HIV-1 protein Vpu antagonizes the antiviral activity of tetherin.^16^^,^^19^ Restriction factors with broad antiretroviral activity are attractive targets for next-generation therapeutics.

The host protein CCHC-type zinc-finger-containing protein 3 (ZCCHC3; [GeneBank: NP_149080.2]) has several links to antiviral immunity and was previously reported to interact with HIV-1 Gag,^20^ but its role in the HIV-1 life cycle has not been thoroughly investigated. ZCCHC3 has been shown to function with cGAS,^21^ RIG-I,^22^ and TLR3,^23^ major cytoplasmic nucleic acid sensors that activate the innate immune signaling pathway, to regulate interferon-stimulated gene (ISG) expression, resulting in the degradation of viral RNA.^24^ ZCCHC3 acts together with cGAS to recognize double-stranded (ds)DNA and with RIG-I and TLR3 to recognize dsRNA, although no direct physical interaction between ZCCHC3 and these proteins has been reported. Porcine ZCCHC3 has also been shown to suppress the replication of the pseudorabies virus by activating IFN-β expression.^25^ Here, we sought to test the hypothesis that ZCCHC3 is an HIV-1 restriction factor that functions through its interaction with Gag.

## Results

### ZCCHC3 and retroviral infectivity

**Figure 1:**
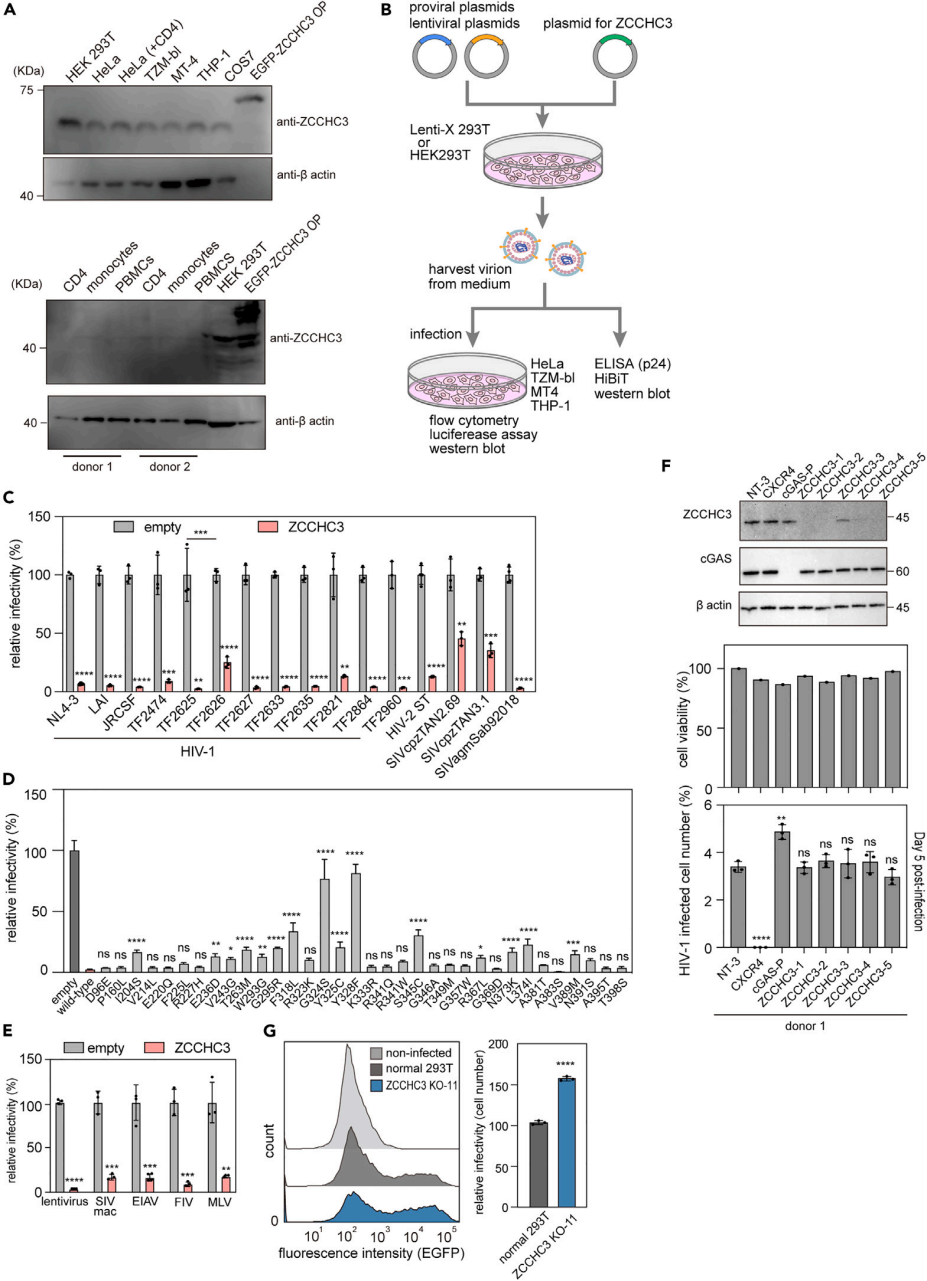
Effect of ZCCHC3 on viral infection (A) ZCCHC3 expression in different cell types, determined in cell lysates by western blotting with an anti-ZCCHC3 antibody. HEK293T cells expressing EGFP-tagged ZCCHC3 were the positive control. The control lane contained five times less lysate than the other lanes. The panel shown here is a representative image of three independent experiments. (B) Experimental flow overview for panels in Figures 1 and 2. Producer cells (HEK293T or Lenti-X 293T) were transfected with viral plasmids and a ZCCHC3 expression plasmid, and the virions were collected from the culture medium and then analyzed as indicated. (C) Effect of ZCCHC3 on the infectivity of various viral strains. Plasmids encoding individual viral strains were introduced into Lenti-X 293T cells in the presence or absence of an HA-ZCCHC3 expression plasmid. Culture supernatant was collected 2 days after transfection and used to infect TZM-bl cells. Infectivity was determined as relative light units of luciferase 2 days after infection. The mean and standard deviation values are shown (n = 3). (D) Effect of SNPs in human *ZCCHC3* gene on viral infectivity of lentiviral vectors. Lenti-X 293T cells were co-transfected with pWPI-Luc2, psPAX2-IN/HiBiT, and pMD2.G with or without an HA-ZCCHC3 expression plasmid carrying a missense mutation. Culture supernatant was collected 2 days after transfection and used to infect MT4 cells. Infectivity was determined 2 days after infection. Values relative to those for cells infected with a lentiviral vector produced with empty vector are shown as the mean ± standard deviation. (E) Effect of ZCCHC3 on viral infectivity of retroviral vectors. A plasmid encoding the indicated retrovirus and luciferase reporter gene was introduced into Lenti-X 293T cells with or without an HA-ZCCHC3 expression plasmid. Culture supernatant was collected 2 days after transfection and used to infect MT4 cells. Infectivity was determined as in (C). Values relative to those for cells harboring empty vector are shown as the mean ± standard deviation. (F) Effect of *ZCCHC3* knockout on viral infection in primary CD4^+^ T cells. Knockout cells were generated by CRISPR-Cas9 gene editing and infected with a GFP reporter HIV-1_NL4-3_ virus in a spreading infection assay. Infection rates were determined by flow cytometry as percent GFP+ cells. The upper panel shows the western blot for the indicated proteins. Bar charts show cell viability (middle) and fold change in infection rates at day 5 (bottom) post-infection. A representative donor out of three shown with average of technical triplicates ±standard deviation. The results from other donors are shown in Figure S1F. (G) Effect of *ZCCHC3* knockout on viral production. Lentiviruses produced in WT and *ZCCHC3*-knockout Lenti-X 293T cells were normalized to p24 and used to infect HeLa cells. The expression of a viral gene (EGFP) was assessed using flow cytometry (left). EGFP-positive cells were quantified (right); the mean and standard deviation values are shown (n = 3). Differences were examined using a two-tailed, unpaired Student’s *t* test; ∗∗∗∗p < 0.0001, ∗∗∗p < 0.001, ∗∗p < 0.01. In (C) TF2625 and TF2626, and (F), differences were examined using one-way ANOVA, followed by Tukey’s test; ∗∗∗p < 0.001, ns p ≧ 0.05.

We first confirmed that ZCCHC3 was expressed in a wide variety of cell types, including epithelial, monocytic, and T cell lines, though it is much lower in several subsets of primary peripheral blood mononuclear cells (Figure 1A). To examine the effect of ZCCHC3 on HIV-1 infectivity, we transfected Lenti-X 293T cells with a pNL4-3 plasmid encoding a replication-competent HIV-1_NL4-3_ virus, with or without a plasmid encoding human influenza hemagglutinin (HA)-tagged human ZCCHC3 (Figure 1B). Human ZCCHC3 cDNA was used in all experiments unless indicated otherwise. We then harvested the released virions and tested their infectivity on TZM-bl cells. TZM-bl is a reporter cell line that expresses a luciferase reporter protein upon infection dependent on the viral Tat protein.^26^^,^^27^^,^^28^^,^^29^^,^^30^^,^^31^ We observed a clear cytopathic effect in the HIV-1_NL4-3_-infected TZM-bl cells (Figure S1A, bottom right panel), but this was largely negated in the presence of ZCCHC3 (Figure S1A, bottom left panel). Consistent with these observations, ZCCHC3 overexpression reduced the infectivity of HIV-1_NL4-3_ by two orders of magnitude as measured by luciferase reporter activity (Figure 1C). We observed a similar effect with different HIV-1 strains, HIV-2, and simian immunodeficiency virus from chimpanzees (SIVcpz), although the extent of suppression varied by strain and subtype (Figure 1C).

We next tested the impact of several naturally occurring single nucleotide polymorphisms (SNPs) in the human *Zcchc3* gene on antiviral activity. We identified 2,440 SNPs in the human *ZCCHC3* gene (NCBI: https://www.ncbi.nlm.nih.gov/snp/?term=zcchc3, accessed on 11/16/2023). Of these SNPs, 388 are missense mutations that result in non-synonymous mutations. We generated 34 ZCCHC3 expression vectors with different SNPs and compared their antiviral activity with wild-type (WT) ZCCHC3. While a majority showed comparable antiviral activity with WT ZCCHC3, several SNPs such as G324S and Y328F had statistically decreased activity compared with WT (Figures 1D and S1B). Furthermore, the extent of inhibition was proportional to the amount of ZCCHC3 plasmid used for viral production, consistent with a dose-dependent effect (Figure S1C). These findings suggest that the antiviral activity of ZCCHC3 is not a non-specific effect of overexpression, but rather represents a novel antiviral mechanism with host and viral determinants.

HIV-1 cell entry is triggered by binding between the viral envelope (Env) and the cellular receptor (CD4) and co-receptor (CCR5). To test whether the antiviral effect of ZCCHC3 is HIV-1 Env-dependent, we used an HIV-1 Δ*env* GFP reporter virus (pMSMnG) pseudotyped with a VSV-G envelope that enables cell entry through a clathrin-mediated endocytic route.^32^ We observed an antiviral effect of ZCCHC3 similar to that seen in the initial experiment, indicating that the antiviral effect of ZCCHC3 is HIV-1 Env-independent (Figure S1D). While we observed comparable inhibition of infectivity by ZCCHC3 in TZM-bl and CD4^+^ T cell-derived C8166-CCR5 cells, the inhibitory effect was more pronounced in THP-1 cells (Figure S1D), suggesting that the antiviral effect of ZCCHC3 may also depend on the target cell type. In addition to WT HIV-1, we tested two HIV-1 Gag capsid (CA) mutants that cannot interact with CPSF6 (N74D)^33^ or CypA (RGDA/Q112D + Q4R).^34^ Previous studies demonstrated that CPSF6^33^ and CypA^34^ are involved in the early steps of HIV-1 replication, binding to the capsid core to regulate reverse transcription, uncoating, nuclear entry, and integration site targeting of proviral DNA (reviewed in a study by Lu et al.^35^). While WT and N74D viruses showed comparable sensitivity to ZCCHC3, the RGDA/Q112D + Q4R virus was slightly more resistant to suppression (Figure S1E). This suggests that CypA-binding or CA sequence may affect the susceptibility of HIV-1 to ZCCHC3.

To test whether ZCCHC3 antiviral activity is antagonized by antiviral proteins, we used an HIV-1-based lentiviral vector, psPAX2-IN/HiBiT, that lacks accessory proteins such as Vif, Vpu, Vpr, and Nef. We observed that ZCCHC3 suppresses the infectivity of psPAX2-IN/HiBiT to an extent similar to that of HIV-1, suggesting that the accessory proteins do not negate the effect of ZCCHC3, at least upon overexpression (compare Figures 1C and 1E). Notably, ZCCHC3 also suppressed the infection of other retroviruses, such as SIV from rhesus macaques (SIVmac), feline immunodeficiency virus (FIV), equine infectious anemia virus (EIAV), and murine leukemia virus (MLV) (Figure 1E), suggesting that ZCCHC3 inhibits the infection of a broad range of retroviruses.

To explore the role of ZCCHC3 in HIV-1 replication in primary CD4^+^ T cells, we next used CRISPR-Cas9 gene editing to knockout the protein in cells from three independent blood donors alongside non-targeting, CXCR4, and cGAS controls. Four of the five independent CRISPR guides resulted in efficient knockout of *ZCCHC3* (Figure 1F, upper panel; Figure S1F, upper panel) and had little impact on cell viability across all donors (Figure 1F, middle panel; Figure S1F, middle panel). These cells were subsequently challenged with a GFP reporter HIV-1_NL4-3_ virus and spreading infection was monitored over 5 days by flow cytometry. At 5 days post-challenge, there was no significant difference in the percent infected cells in the *ZCCHC3* knockout versus the non-targeting cells in any donor (Figure 1F, lower panel; Figure S1F, lower panel). Knockout of the CXCR4 co-receptor, however, resulted in strong restriction of replication. These data suggest that basal levels of ZCCHC3 in primary CD4^+^ T cells do not restrict HIV-1 replication, though this may be due to the low basal level of ZCCHC3 expression in this cell type (Figure 1A).

To determine if ZCCHC3 depletion would have an effect in a cell line with higher basal levels of expression, we generated *ZCCHC3* knockdown Lenti-X 293T producer cells with three independent siRNA (Figure S1G). Viruses produced from these lines were statistically more infectious (Figure S1H). To confirm these results, we then generated monoclonal *ZCCHC3* knockout Lenti-X 293T producer cells using CRISPR-Cas9 (Figure 1G, more clones in Figures S1I and S1J). Finally, we cloned ZCCHC3 from different species, which suppressed the infectivity of HIV-1 JR-CSF and TF2625 strains similarly to human ZCCHC3 (Figures S1K and S1L). These observations suggested that the antiretroviral activity is conserved in mammals. Notably, the HIV-1 TF2625 strain, which exhibited higher sensitivity to human ZCCHC3 than TF2626 (Figure 1C), was also more sensitive to ZCCHC3 proteins from other species (Figures S1K and S1L), suggesting a viral strain-specific resistance. Taken together, these results suggest that ZCCHC3 suppresses the infectivity of HIV-1 and other retroviruses with the extent of restriction dependent on producer cell expression level, viral strain, and target cell type.

### ZCCHC3 and viral production

**Figure 2:**
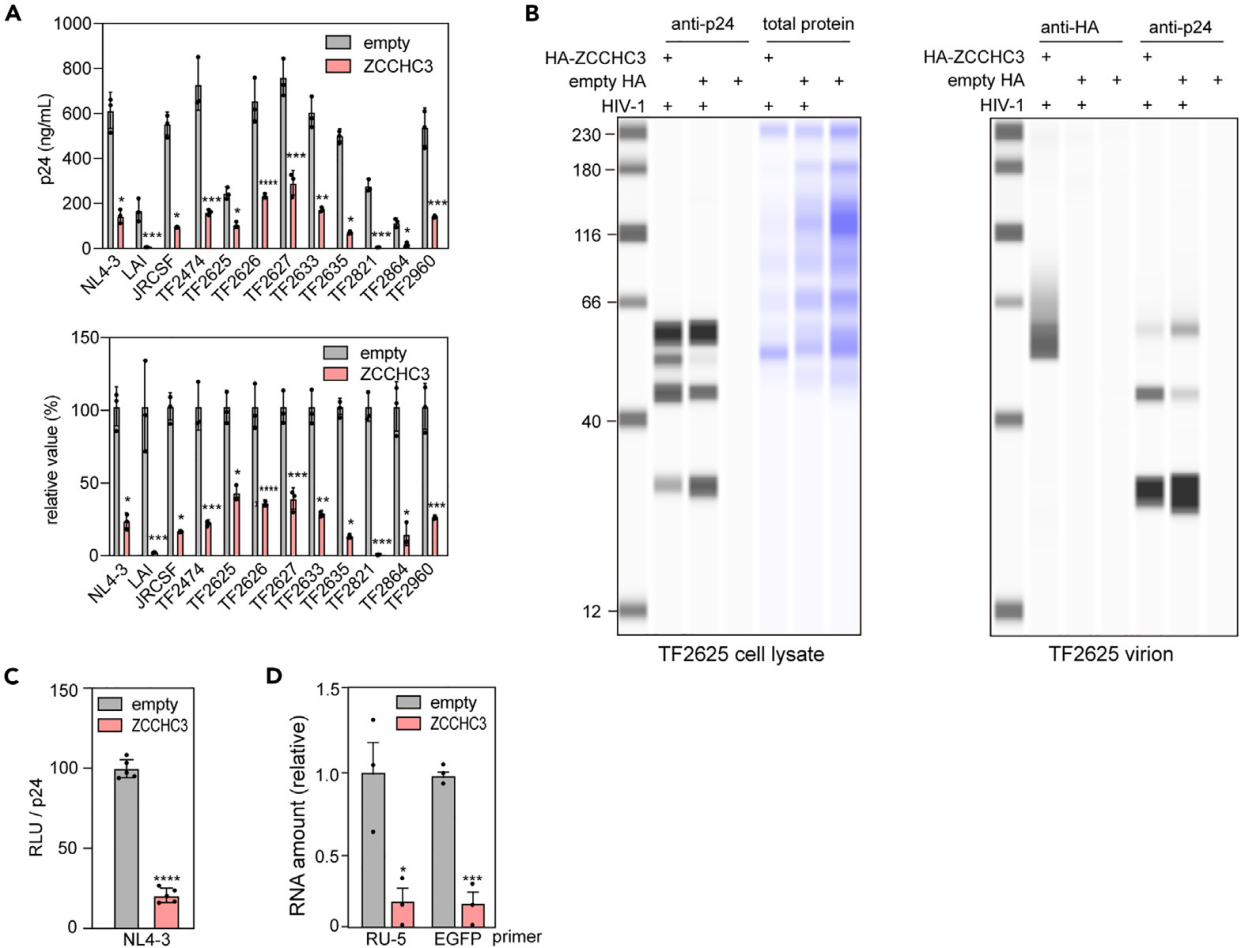
Effect of ZCCHC3 on viral production (A) Effect of ZCCHC3 on HIV-1 viral production. A plasmid encoding HA-tagged ZCCHC3 or empty HA-vector was introduced into Lenti-X 293T cells together with an HIV-1-encoding plasmid. Virions released into the culture medium were quantified by p24 ELISA. The absolute value (top) and the value relative to that without ZCCHC3 (bottom) are shown (n = 3). (B) ZCCHC3 affects the Gag processing. Lenti-X 293T cells were co-transfected with pTF2625 plasmids in the presence or absence of HA-ZCCHC3. Equal volumes of the pelleted virions and cell lysates were analyzed via western blotting using anti-p24 and anti-HA antibodies. The total protein in the cell lysate was also analyzed via CBB staining. (C) Effect of ZCCHC3 on infectivity of ZCCHC3-loaded virions. TZM-bl cells were infected with the same amount (p24-normalized) of HIV-1_NL4-3_ or lentivirus with or without ZCCHC3 in the virion. Infectivity was analyzed by luciferase assay and is presented relative to that without ZCCHC3 as the mean and standard deviation (n = 5). (D) Effect of ZCCHC3 co-expression on lentivirus production. HEK293T cells were transfected with lentiviral plasmids (pLV-EGFP, psPAX2, and pMD2.G) with or without an HA-ZCCHC3 expression plasmid. Total RNA was purified from lentiviruses harvested from the culture medium and analyzed by RT-qPCR. The mean and standard deviation values are shown (n = 3). In (A), (C), and (D), differences were examined by a two-tailed, unpaired Student’s *t* test; ∗∗∗∗p < 0.0001, ∗∗∗p < 0.001, ∗∗p < 0.001, ∗p < 0.05.

We next assessed if ZCCHC3 decreases HIV-1 infectivity by affecting viral production. Accordingly, we quantified virions released from pNL4-3-transfected Lenti-X 293T cells into the culture medium by enzyme-linked immunosorbent assay (ELISA) of viral p24 protein. Co-expression of ZCCHC3 with HIV-1 proviral plasmids reduced the production of virions by all the viruses tested (Figure 2A). Titrating the amount of exogenous ZCCHC3 relative to endogenous ZCCHC3 demonstrated that doubling the expression of ZCCHC3 reduced viral production by 40–50% (Figure S2A), excluding a possible artifact of over-production. In agreement with this result, viral production was increased by up to 72% upon *ZCCHC3* knockout in Lenti-X 293T cells (Figure S2B, KO-11). Western blot analysis using anti-p24 antibody revealed that the amount of virion released into the medium, but not the amount of Gag in the producer cells, was reduced by ZCCHC3 co-expression (Figure 2B, right panel). In addition, we found that ZCCHC3 affected the processing of Gag as shown by an increase in the amount of p41 and a reduction in p24 (Figure 2B, left panel). Together, these results suggested that ZCCHC3 reduces the amount of virion production and produces abnormal virions.

To test the infectivity of the ZCCHC3-containing virion, we infected TZM-bl cells with a p24-normalized virions. Notably, ZCCHC3-containing HIV-1_NL4-3_ virions showed lower infectivity than those produced by non-ZCCHC3-overexpressing cells (Figure 2C). This suggested that virions produced in the presence of ZCCHC3 are defective and have reduced infectivity. Next, to start dissecting the underlying mechanism, we analyzed the amount of viral RNA in virions. We harvested lentiviruses produced by Lenti-X 293T cells in the absence or presence of ZCCHC3 overexpression and analyzed the p24-normalized virions by reverse transcription quantitative polymerase chain reaction (RT-qPCR). Indeed, the amount of viral RNA in the virions was greatly reduced upon ZCCHC3 co-expression (Figure 2D). This result is in consensus with previous reports that the viral RNA is involved in the viral packaging and production,^36^ as well as the processing of Gag. Together, these results indicated that ZCCHC3 inhibits the packaging of viral RNA and reduces the production of infectious virus particles.

### ZCCHC3 domains for GagNC-RNA binding

**Figure 3:**
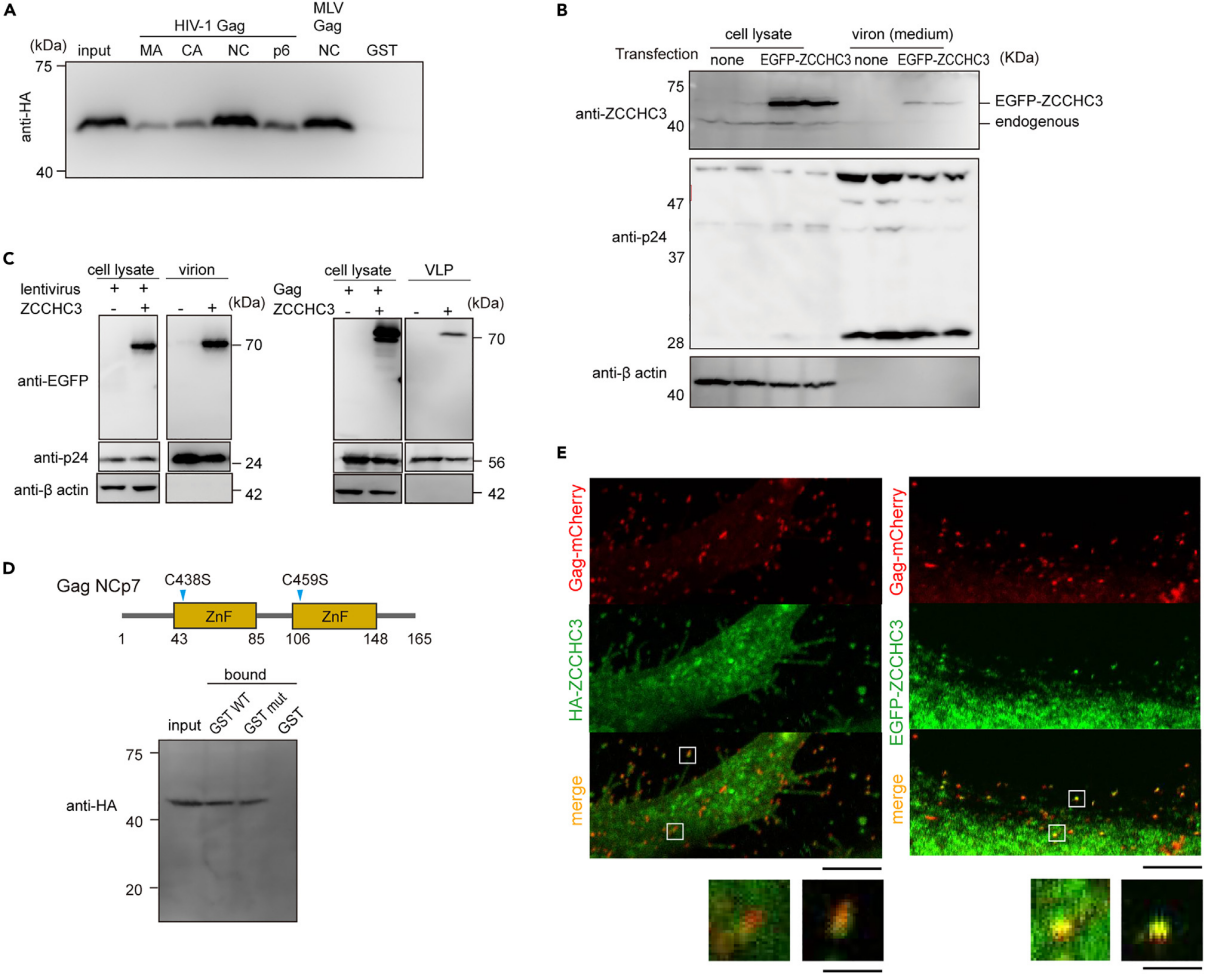
ZCCHC3 binding to GagNC (A) ZCCHC3 binding to NCp7 of HIV-1 Gag. GST-tagged HIV-1 Gag MAp17, CAp24, NCp7 or p6 protein, or MLV Gag NC was mixed with HEK293T cell lysate containing HA-ZCCHC3 and GSH beads after treatment with RNase A. The eluted fraction was analyzed by western blotting using an anti-HA antibody. A representative image from three independent experiments is shown here. See also Figure S3A. (B) ZCCHC3 incorporated into the HIV-1 TF2625 virion. Lenti-X 293T cells were transfected with pTF2625 plasmid in the presence or absence of an EGFP-ZCCHC3 expression plasmid. Pelleted virions were analyzed via western blotting with anti-ZCCHC3, anti-p24, and anti-β-actin antibodies. (C) Lentiviral plasmids (pLV-EGFP, psPAX2, and pIIIenv3-1) (left) or a plasmid encoding HIV-1 Gag (right) were introduced into 293T cells with or without an HA-ZCCHC3 expression plasmid. Virions were harvested by centrifugation, and analyzed by immunoblotting with anti-EGFP, anti-p24, and anti-β-actin antibodies. (D) The zinc-finger motifs of GagNCp7 are not necessary for interaction with ZCCHC3. GST-tagged GagNCp7 (WT or mutant) was mixed with the cell lysate containing HA-ZCCHC3 and GSH beads. The position of the mutations (C to S) in GagNCp7 is depicted in the top panel. The bound fraction was analyzed via western blotting using an anti-HA antibody. A representative image from three independent experiments is shown here. See also Figure S3B. (E) Presence of ZCCHC3 in HIV-1 Gag VLP. COS7 cells expressing HA-ZCCHC3 and mCherry-HIV-1 Gag were fixed, stained with an anti-HA antibody, and observed using confocal laser scanning microscopy (CLSM) (left). COS7 cells expressing EGFP-ZCCHC3 and mCherry-HIV-1 Gag were fixed and observed using CLSM (right). Enlarged images are also shown at the bottom. Scale bars, 5 μm (top), 25 μm (bottom).

Our previous results suggested that ZCCHC3 interferes with Gag processing and viral genome incorporation into viral particles. To determine if this is due to a direct interaction with Gag, we performed a pull-down assay with different glutathione *S*-transferase (GST)-tagged Gag fragments: matrix (MAp17), capsid (CAp24), nucleocapsid (NCp7), and p6. The experiment revealed that ZCCHC3 bound to HIV-1 GagNCp7 and MLV GagNC (Figures 3A and S3A). In agreement with this result, we also detected ZCCHC3 (endogenous and exogenous) in virions released into the culture medium by Lenti-X 293T cells (Figure 2B, right panel, 3B). In fact, ZCCHC3 was present not only in TF2625 virions (Figure 2B, right panel), but also in lentiviral virions and virus-like particles (VLPs) produced by HIV-1 Gag in the absence of other viral components (Figure 3C), indicating that viral RNA is not necessary for the incorporation. Mutating cysteine residues in GagNC to abolish the zinc-finger motifs did not affect the interaction with ZCCHC3 (Figures 3D and S3B), suggesting that the zinc-finger motif itself is not necessary for the interaction. Subsequent analysis with COS7 cells expressing HA-ZCCHC3 or enhanced green fluorescent protein (EGFP)-fused ZCCHC3 and Gag-mCherry confirmed that ZCCHC3 co-localized with Gag both inside the producer cell and in the released VLPs (Figure 3E). This demonstrated that a direct interaction between ZCCHC3 and GagNC is sufficient for the incorporation of ZCCHC3 into the virion.

**Figure 4:**
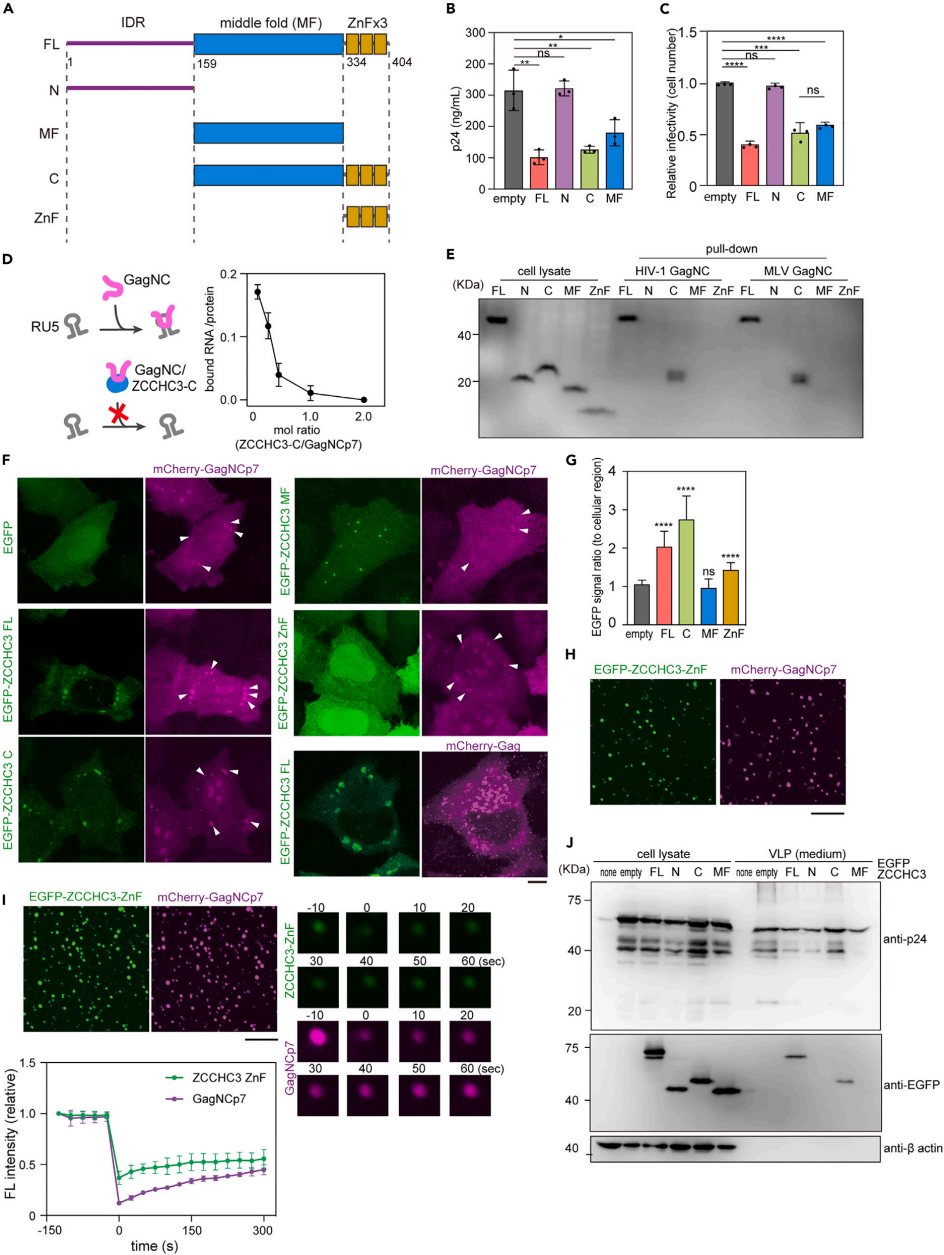
ZCCHC3 binding to GagNC via C-terminal domain (A) The domain structure of human ZCCHC3. IDR, MF, and zinc-finger (ZnF) domains are indicated. (B and C) Effect of C-terminal fragment of ZCCHC3 on viral production and infectivity. HEK293T cells were transfected with lentiviral vectors, and a ZCCHC3 FL, N, C, or MF expression vector or an empty vector. The resultant lentiviruses were harvested 2 days after transfection and quantified by p24 ELISA (B). HeLa cells were infected with a p24-normalized amount of harvested lentiviruses, and infectivity was quantified based on the expression of a viral gene (EGFP) using flow cytometry (C). The mean and standard deviation values from three independent experiments are shown. (D) Hypothetical mechanism of ZCCHC3 inhibition of the interaction between Gag NC and viral RNA and the effect of ZCCHC3 on the interaction between HIV-1 GagNCp7 and LTR RNA. Purified ZCCHC3 was tested in RNA pull-down assay of GagNCp7 and HIV-1 LTR, at the molar ratios indicated. A fluorescent probe was used to quantify RNA (ng) in the bound fraction, which is presented as a ratio to the amount of the bait protein (ng) in the bound fraction quantified by Coomassie brilliant blue (CBB) staining. The mean and standard deviation values from three independent experiments are shown. (E) Binding of different ZCCHC3 domains to GagNCp7. GST-tagged HIV-1 or MLV Gag NC was mixed with HEK293T cell lysate containing HA-tagged ZCCHC3 FL, N, C, MF, or ZnF fragments after treating with RNase A, and the eluted fraction was analyzed using western blotting with anti-HA antibody. See also Figure S3F. (F and G) Localization of HIV-1 GagNCp7 and ZCCHC3 in HeLa cells. HeLa cells expressing mCherry-Gag NCp7 and EGFP-tagged ZCCHC3 FL, C, MF, or ZnF fragments were fixed and observed using CLSM (F). The arrowheads indicate the cytoplasmic GagNCp7 condensate. Scale bar, 5 μm. The fluorescence intensity ratio of GagNCp7 foci and cytoplasm was quantified (n = 15) (G). (H and I) ZCCHC3 ZnF domain co-localizes with GagNCp7 *in vitro*. *In vitro* droplet assay using recombinant His_x6_-EGFP-ZCCHC3 ZnF and His_x6_-mCherry-GagNCp7. Both proteins formed droplets in the presence of 15% polyethyleneglycol (H). Scale bar, 100 μm. Both proteins co-localized in the same droplet when they were mixed together (I, top left). FRAP analysis was performed against the droplet to measure the mobility of the proteins. Representative images are presented (right). The average fluorescence intensity of the bleached region was quantified and plotted against time as a value relative to that of the pre-bleached signal. Data are presented as mean ± standard deviation from three independent measurements. Scale bar, 100 μm. (J) Incorporation of ZCCHC3 domains into Gag VLP. Plasmid encoding EGFP-tagged ZCCHC3 FL, N, C, or MF was introduced into HEK293T cells together with a plasmid encoding HIV-1 Gag. VLPs released into the culture medium were harvested and analyzed by immunoblotting with anti-GFP, anti-p24, and anti-β-actin antibodies. In (B), (C), and (G) differences were examined by a two-tailed, unpaired Student’s *t* test; ∗∗∗∗p < 0.0001, ∗∗∗p < 0.001, ∗∗p < 0.01, ns, p ≥ 0.05.

We next assessed which ZCCHC3 domain is involved in the interaction with GagNC. ZCCHC3 is composed of an N-terminal intrinsically disordered region (IDR), middle fold (MF) domain, and three C-terminal tandem repeats of CCHC-type zinc-finger motifs (ZnF) (Figure 4A). We expressed these fragments in HEK293T cells and quantified viral production and infectivity. We observed that the IDR fragment did not reduce lentiviral production or infectivity, whereas the C-terminal fragments (MF and ZnF) suppressed both lentiviral production and infectivity (Figures 4B, 4C, and S3C). The C-terminal fragment likewise was sufficient to inhibit other retroviruses in this assay (SIVmac, FIV, and EIAV) (Figure S3D). Together, these results demonstrated that the C-terminal fragment of ZCCHC3 had a suppressive effect on HIV-1 and other retroviruses.

Because GagNC interacts with viral genomic RNA and promotes genome packaging into the virion,^29^ we speculated that ZCCHC3 inhibits the interaction between GagNC and viral RNA (Figure 4D). Accordingly, we tested the effect of the C-terminal fragment of ZCCHC3 on RNA binding of GagNCp7 in an RNA pull-down assay. The presence of the ZCCHC3 fragment decreased the amount of RNA pulled down by GagNC in a dose-dependent manner (Figure 4D). This result supports direct binding of GagNC by ZCCHC3 and inhibition of RNA binding.

As proof-of-concept, we next tested if less viral RNA correlates with less viral production. It is well-established that each viral particle contains a dimerized genomic RNA in its viral core, and the dimerized genomic RNA is critical for an interaction with GagNC.^37^ To this end, we produced a lentiviral vector with different amounts of genome donor plasmid. We observed that increasing the amount of viral RNA augmented viral production (Figure S3E). This observation supports a model by which a reduction of viral RNA packaging by ZCCHC3 in producer cells could lead to decreased virion production. Together, these results demonstrated that the C-terminal fragment of ZCCHC3 inhibits the interaction between GagNC and viral RNA and reduces viral production.

### ZCCHC3 interactions with GagNC

The C-terminal fragment of ZCCHC3 contains three ZnF motifs. ZnFs are functionally versatile structural motifs involved in various types of molecular interactions with DNA, RNA, and protein.^38^ We therefore investigated the role of ZnFs in the antiviral effect of ZCCHC3. A pull-down assay using purified GST-tagged GagNCp7 revealed that while the C fragment of ZCCHC3 strongly bound to GagNCp7 (Figures 4E and S3F), the deletion of ZnFs (the MF fragment) almost completely abolished the interaction. This demonstrated that ZnF domain is necessary for the interaction of ZCCHC3 and GagNCp7. However, we could not detect a direct interaction between the ZnF domain of ZCCHC3 and GagNCp7 in the pull-down assay (Figure 4E), suggesting that the MF fragment is also involved in the interaction.

We then probed ZnF-mediated interaction with Gag in cultured cells. The full-length ZCCHC3 co-localized with both Gag and GagNCp7 (Figures 4F and 4G). Notably, mCherry-fused GagNCp7 and EGFP-fused ZCCHC3 C fragment formed a liquid-like condensate in the cytoplasm (Figures 4F and S3G). In agreement with the pull-down assay (Figure 4E), the deletion of the entire ZnF domain (the MF fragment) severely abrogated the co-localization, and the ZnF domain only weakly associated with GagNCp7 (Figures 4F and 4G). To further examine the interaction between ZCCHC3 ZnF domain and GagNCp7, we prepared recombinant proteins of EGFP-tagged ZCCHC3 ZnF and mCherry-tagged GagNCp7 and examined whether they co-existed in the same liquid-like condensate *in vitro*. EGFP-ZCCHC3 ZnF domain and mCherry-GagNCp7 independently form liquid-like condensate in the presence of polyethylene glycol (Figure 4H) and they co-existed in the same droplet when they were mixed together (Figure 4I). This demonstrated that the ZnF domain of ZCCHC3 weakly interacts with GagNCp7. Notably, the ZnF-mediated interaction with Gag was necessary for ZCCHC3 incorporation into the virion: the C fragment, but not the MF fragment, was incorporated into Gag VLPs (Figure 4J) and lentiviral virions (Figure S3H). Altogether, these results indicate that the ZCCHC3 ZnF domain interacts with GagNCp7 and plays a role in the incorporation into the virion, though this is also dependent on the MF domain.

### ZCCHC3 interactions with retroviral RNA

**Figure 5:**
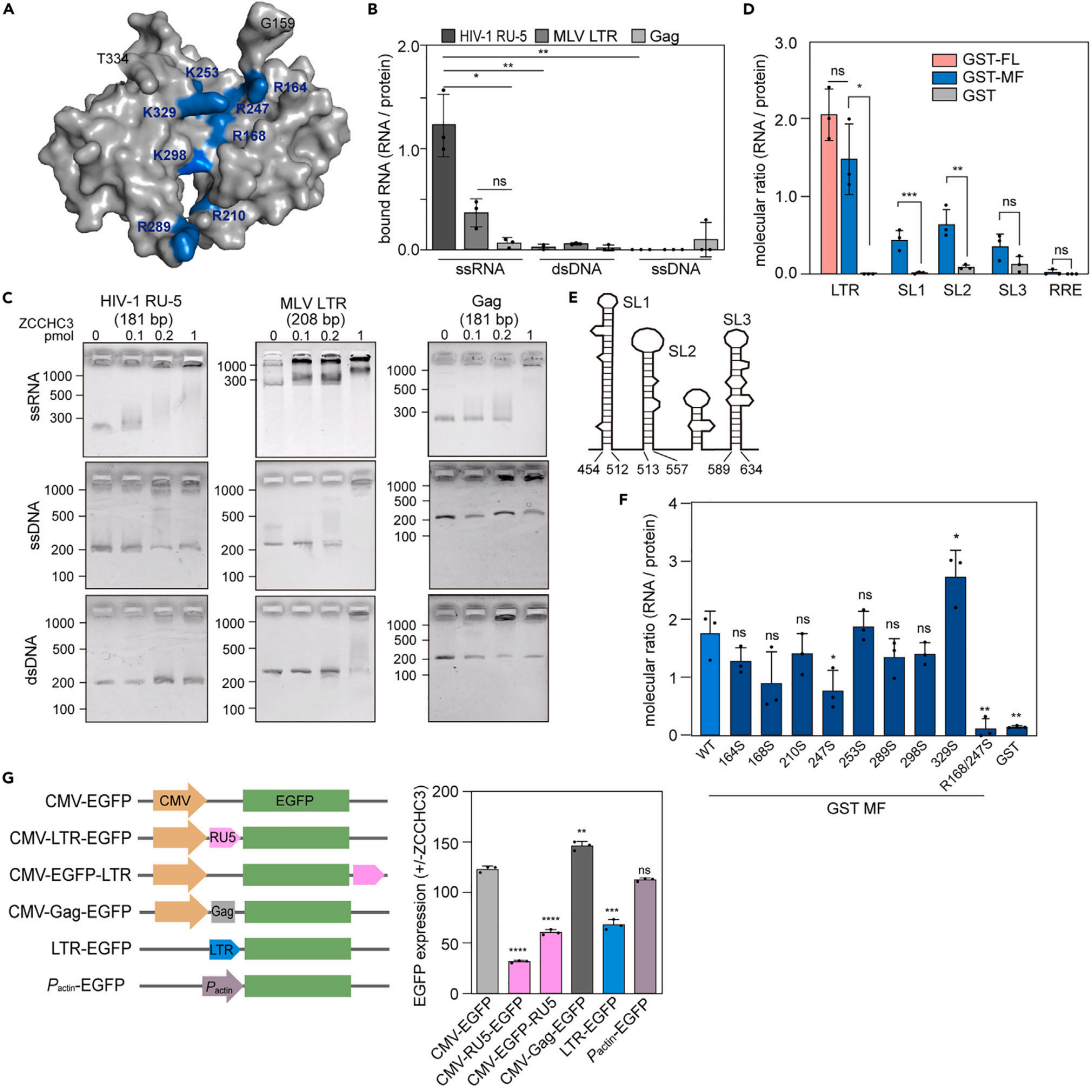
ZCCHC3 binding to retroviral RNA (A) 3D structure of ZCCHC3 MF region predicted by using AlphaFold2. Basic residues in the central cleft are labeled in blue. (B) ZCCHC3 MF domain binding to LTR RNAs of HIV-1 and MLV. GST-tagged ZCCHC3 MF or GST was mixed with ssRNA, dsDNA, or ssDNA of HIV-1 LTR (R-U5), MLV LTR, or a coding region of Gag. Nucleic acids in the bound fraction were quantified using a fluorescent probe, and the amount is presented as a molecular ratio to the bait protein. The mean and standard deviation values from three independent experiments are shown. (C) EMSA analysis of ZCCHC3–HIV-1 genome interaction. Different amounts of purified ZCCHC3 were incubated with ssRNA, dsDNA, and ssDNA (0.1 pmol, prepared as in B) and analyzed. (D) Binding of ZCCHC3 domains to LTR, stem-loops of the LTR and RRE. RNA pull-down assay was performed with HIV-1 LTR (R-U5), SL1, SL2, SL3 RNA or HIV-1 RRE and GST-tagged ZCCHC3 FL or MF as described in (B). The amount of bound RNA is presented as the molecular ratio to the bait protein, with the mean and standard deviation values from three independent experiments shown. (E) Schematic illustration of HIV-1 LTR (R-U5) secondary structure. (F) Binding of ZCCHC3 MF domain WT and basic amino acid mutants to the stem-loops of HIV-1 LTR (R-U5). RNA pull-down assay was performed with the HIV-1 LTR (R-U5), and GST-ZCCHC3 MF (WT or mutants), as described in (D). The amount of bound RNA is presented as the molecular ratio to the bait protein, with the mean and standard deviation values from three independent experiments shown. (G) ZCCHC3 suppresses the expression of LTR-containing genes. The promotor constructs are depicted (left). HIV-1 LTR (R-U5) was inserted upstream or downstream of the *EGFP* ORF. A fragment of the HIV-1 Gag gene (181 bp) was used as a control. Some constructs carry HIV-1 LTR (full length) or chicken β-actin promoter in place of the CMV promoter. The EGFP reporter constructs were introduced into HEK293T cells with or without an HA-ZCCHC3 expression plasmid, and EGFP fluorescence was quantified by flow cytometry. Quantification of EGFP-expressing cells as the ratio of signal between cells with (+) and without (−) HA- ZCCHC3 is shown (right). The mean and standard deviation values from three independent experiments are shown. Differences in B, D, F and G were examined by a two-tailed, unpaired Student’s *t* test. ∗∗∗p < 0.001, ∗∗p < 0.01, ∗p < 0.05; ns, p ≥ 0.05.

We next examined the role of the MF domain in the antiviral effect of ZCCHC3. Although the MF domain does not bind to GagNCp7 in our pull-down assay (Figure 4E) and facilitates ZnF binding, it was sufficient to inhibit the infectivity of HIV-1 (Figures 4B and 4C). These observations suggested that the MF domain may inhibit viral production by a mechanism distinct from the ZnF-dependent interaction with Gag. According to a structural prediction by AlphaFold2,^39^ the MF domain contains a basic cleft (Figure 5A). Furthermore, a catRAPID prediction of RNA–protein interactions^40^ identified several potential binding sites for HIV-1 long terminal repeats (LTRs) within the MF domain (Figure S4A; Table S3). These findings suggest that ZCCHC3 may also bind to viral RNA to inhibit the packaging of genomic RNA or the production of infectious virions.

Accordingly, we investigated the interaction between the MF domain of ZCCHC3 and the viral genome. An RNA pull-down assay revealed that ZCCHC3 MF bound to the R-U5 region of HIV-1 LTR and MLV LTR, but not to the coding region of the HIV-1 *gag* (Figure 5B). The protein also bound to dsDNA and ssDNA molecules carrying the same nucleotide sequences, but with a much lower affinity (Figure 5B). We obtained similar results in electrophoretic mobility shift assay (EMSA) (Figure 5C). Unlike the binding to GagNCp7, the MF domain was sufficient for binding to HIV-1 LTR (Figure 5D) and to LTRs of other retroviruses (Figure S4B).

The LTR of HIV-1 contains three stem-loop structures (Figure 5E). To identify the region of HIV-1 LTR that interacts with ZCCHC3 MF, we performed an RNA pull-down assay with the individual stem-loops, which revealed that ZCCHC3 MF bound to all these structures (Figure 5D). Notably, ZCCHC3 MF did not bind to the RRE region of the HIV-1 genome (Figure 5D), which also contains several stem-loop structures, suggesting that ZCCHC3 may recognize the stem length of a stem-loop structure. Further, screening of ZCCHC3 MF variants with single substitutions of basic amino acid residues in the middle basic cleft (Figure 5A) identified two residues (R168 and R247) involved in RNA binding; a single substitution (R168S and R247S) reduced the affinity for LTR RNA to approximately 50% of that of the WT, and the double substitution (R168/247S) further reduced to approximately 15% (Figure 5F). In contrast, the incorporation of ZCCHC3 into the virion was not affected by the R168/247S mutations (Figure S4C). This suggests that the amino acids responsible for RNA recognition are not associated with Gag NC binding. Put together, these results demonstrate that ZCCHC3 binds to LTR RNA via its MF domain.

### ZCCHC3 and viral protein expression

Next, we constructed several reporter genes to confirm the LTR requirement for ZCCHC3-dependent suppression of protein expression (Figure 5G). The expression of the reporter protein (EGFP) under the control of a cytomegalovirus (CMV) promoter/enhancer was not affected by the co-expression of ZCCHC3 (Figures 5G and S4D). The addition of R-U5 region of HIV-1 LTR at either end of the protein-coding region conferred sensitivity on the reporter protein amount to ZCCHC3 (Figures 5G and S4D), whereas insertion of a fragment of the Gag-coding region of a similar nucleotide length did not (Figures 5G and S4D). Notably, ZCCHC3 expression did not alter the mRNA abundance of any of the reporter genes (Figure S4E). The observed suppression of reporter gene expression was in accordance with the *in vitro* assays of RNA binding, demonstrating that the stem-loops in the R-U5 region are the major binding site of ZCCHC3 on viral RNA (Figure 5D). Further, the expression of the reporter driven by HIV-1 LTR was also reduced in the presence of ZCCHC3 (Figure 5G). We used the TZM-bl cell line, which harbors the luciferase reporter gene within the genome, and the expression level of luciferase is activated by the viral Tat protein to test whether the suppression of reporter gene expression occurs on the proviral state gene. The expression of ZCCHC3 in TZM-bl cells suppressed the expression of a luciferase reporter gene (Figure S4F). Together, these results indicated that the expression of mRNA carrying HIV-1 LTR is suppressed by ZCCHC3.

### ZCCHC3 and viral RNA in P-body

**Figure 6:**
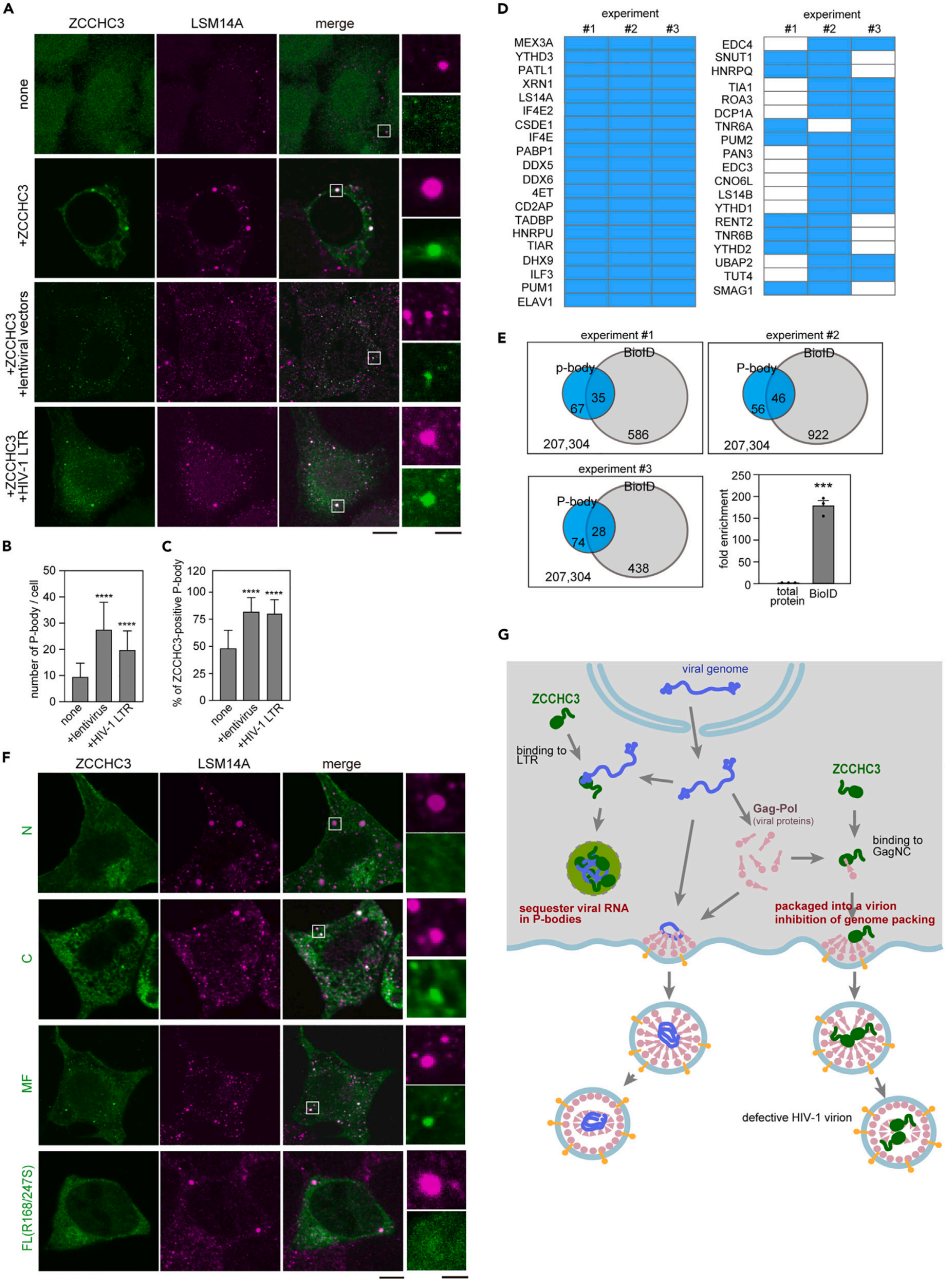
ZCCHC3 sequestration of viral RNA in P-body (A–C) ZCCHC3 localization in the P-body. HEK293T cells were non-transfected or transfected with EGFP-ZCCHC3-encoding plasmid, lentiviral plasmid, or a plasmid carrying HIV-1 LTR, and immuno-stained with anti-LSM14A and anti-ZCCHC3 antibodies. Representative images are shown (A). Scale bar, 5 μm. The number of LSM14A-positive foci per cell was counted (20 cells in each condition) and is summarized in (B). The fraction of ZCCHC3-containing P-bodies was counted and is summarized in (C). (D and E) BioID analysis of 293T cells stably expressing TurboID-fused ZCCHC3. P-body proteins were extracted from the set of identified proteins using GO terms and are shown for three independent experiments (D). The original data of mass spectrometry is provided in Table S2. The total numbers of proteins identified in individual experiments are summarized in a Venn diagram (E). Fold enrichment of P-body proteins is shown as the mean and standard deviation (n = 3; E, right bottom panel). (F) The role of ZCCHC3 MF domain in P-body localization. HEK293T cells expressing EGFP-tagged fragments (N, MF, C) of ZCCHC3 or ZCCHC3 FL carrying R168/248S mutations were immuno-stained with an anti-LSM14A antibody and observed using CLSM. Scale bars, 5 μm (left panels), 1 μm (right panels). (G) Proposed mechanism of HIV-1 suppression by ZCCHC3. The 5′ LTR region of nuclear-exported HIV-1 genomic RNA is recognized by the ZCCHC3 MF, and sequestered in the P-bodies, which impairs virion maturation. ZCCHC3 also binds to HIV-1 Gag NCp7, which leads to ZCCHC3 incorporation into the virion and promotes the antiviral function of ZCCHC3 during subsequent infection. Differences in (B), (C), and (E) were examined by a two-tailed, unpaired Student’s *t* test. ∗∗∗∗p < 0.0001, ∗∗∗p < 0.001, ∗p < 0.05.

Previously, proteomic analysis identified ZCCHC3 in the P-body, a membrane-less organelle that sequesters various mRNAs and inhibits protein production.^41^ We therefore speculated that ZCCHC3 delivers viral RNA to the P-body and thus suppresses viral protein production. Indeed, immuno-staining of HEK293T cells with ZCCHC3 and a P-body marker protein (LSM14A) revealed that endogenous ZCCHC3 was barely present in the P-body, and overexpressed ZCCHC3 was more clearly localized in the P-body (5.7 ± 4.2% of total ZCCHC3 was localized in P-bodies) (Figure 6A). Co-transfection of lentiviral vectors, as well as HIV-1 LTR-containing plasmid, increased both the total number of P-bodies and ZCCHC3-containing P-bodies in a cell (Figures 6A–6C). G3BP1, which is a major component of stress granules, was also detected in the ZCCHC3-containing foci (Figure S5A), which agrees with previous studies showing that the P-body and stress granules share some proteins.^42^ To confirm the interaction between ZCCHC3 and the P-body, we performed proximity-dependent biotin identification of proteins (BioID analysis) using TurboID-fused ZCCHC3. We identified 610 proteins (1,228 biotinylated peptides) as spatial neighbors of ZCCHC3, including some P-body proteins (Figure 6D). Enrichment analysis using GO terms revealed a nearly 100-fold enrichment of P-body proteins among the biotinylated proteins (Figure 6E). Further, the MF domain, which binds to LTR RNA, was necessary and sufficient for P-body localization of ZCCHC3 (Figure 6F). The R168/247S mutant, which lacked viral RNA-binding ability (Figure 5F), failed to localize in the P-body (Figure 6F), supporting that localization to the P-body is HIV-1 genomic RNA-dependent. By using IF/FISH (fluorescence *in situ* hybridization) methods, we showed co-localization of HA-tagged ZCCHC3, LSM14A, and the HIV-1 lentivirus gRNA in HeLa CD4^+^ cells 30 min after infection (Figure S5B). Collectively, these results suggested that ZCCHC3 recognizes LTR-carrying RNA and sequesters it to the P-body.

## Discussion

In this study, we demonstrated that ZCCHC3 is a novel HIV-1 restriction host factor that acts on multiple viral components (GagNC and LTR) to suppress viral production and infectivity. We showed that ZCCHC3 binds to GagNC primarily via ZnFs and prevents it from binding to viral RNA. This results in the assembly of ZCCHC3-loaded virions instead of genome-loaded virions (Figure 6G). Further, ZCCHC3 binds to the LTR of viral genomic RNA via the basic pocket in the MF domain and sequesters viral genomic RNA in the P-body (Figure 6G). The broad spectrum of retroviruses inhibited by ZCCHC3 and the unique dual-acting mechanism may make it an attractive target for next-generation therapeutic development.

ZCCHC3 and APOBEC3G, a well-researched HIV-1 restriction factor antagonized by the viral Vif protein, are similar in that both can interact with HIV-1 genomic RNA^43^ and are incorporated in the HIV-1 virion in a GagNC-dependent manner.^44^ However, the antiviral mechanism of ZCCHC3 is clearly distinct from that of APOBEC3G: ZCCHC3 sequesters the viral RNA in the P-body (Figure 6A), whereas APOBEC3G increases the mutation rate at the reverse transcription step of viral life cycle.^43^ Although APOBEC3G was detected in the P-body,^45^ the functional significance of this observation for the antiviral effect of this protein is not known. In addition, unlike the clear role of Vif in antagonizing APOBEC3G, the antiviral activity of ZCCHC3 is not clearly counteracted by Vif, Vpr, Vpu, or Nef (Figures 1C and 1E).^46^^,^^47^^,^^48^ In this study, we observed TF2626 partially evaded inhibition by ZCCHC3, implying that some HIV-1 strains might evolved their viral genome to counteract inhibitory effect by ZCCHC3.

We found that the ZCCHC3-viral RNA interaction is mediated by the MF domain (amino acid [aa] 159–334) but not the ZnF domain (aa 334–404) (Figure 5D). The ZnF domain plays a major role in the protein’s interaction with GagNCp7 (Figures 4E–4J). This result was unexpected since in many host restriction factors, ZnF motifs are mainly involved in viral RNA binding.^49^ Previously, ZCCHC3 ZnF was shown to bind to dsRNA and poly I:C with a K_*d*_ of 40 nM, although the protein fragment assayed in the previous study (aa 300–404) also contained a part of the MF domain.^24^ Mutagenesis experiments presented herein revealed that two basic residues of ZCCHC3, R168 and R247, are involved in viral RNA binding (Figure 5F). These two residues are predicted to be located opposite one another within the middle cleft (Figure 5A). The predicted 3D structure of this RNA binding motif is distinct from that of any other known RNA-binding motifs. Crystal structures of ZCCHC3 MF fragment and its complex with viral RNA are needed to confirm the spatial relationship between the two identified residues, and their roles in interactions with viral RNA. Notably, the MF domain failed to bind to the RRE region in the HIV-1 genome, which contains several stem-loops carrying shorter stems (<24 nt) and smaller loops compared to those in the LTR,^50^ suggesting that ZCCHC3 prefers longer stems. However, we found that relatively ZCCHC3-resistant HIV-1 strain (TF2626, Figure 1C) contains two additional nucleotides in the second stem of the LTR, implying that ZCCHC3 recognizes a certain range of the stem length or a sequence-dependent secondary structure. Further studies will be required for the detailed mechanism of RNA recognition by ZCCHC3.

The interaction between ZCCHC3 (ZnFs) and GagNC is a novel type of protein-protein interaction. Both proteins contain CCHC-type ZnFs (3 and 2, accordingly). We observed that mCherry-fused GagNCp7 formed a liquid-like condensate in the cytoplasm when it was overexpressed in HEK293T cells (Figures 4F; S3G). This supports the findings of a recent study demonstrating that GagNC undergoes liquid-liquid phase separation (LLPS) together with viral RNA, which plays a critical role in viral packaging and maturation.^51^ We found that ZCCHC3 co-localized in GagNC condensate in a ZnF-dependent manner (Figures 4F and 4G). This result implies that CCHC ZnFs undergo LLPS in the intracellular milieu, and that ZCCHC3 utilizes this mechanism to associate with the viral component. Indeed, recent studies demonstrated that CCHC-type ZnF, as well as other types of ZnF, undergo LLPS, both *in vivo* and *in vitro.*^52^^,^^53^ While Zn^2+^-mediated conformation of the CCHC sequence and/or electrostatic interactions among charged residues within and between individual ZnFs could be involved in the ZCCHC3-GagNC interaction, further structural and functional studies are required to elucidate the molecular details.

Overall, here, we demonstrated that ZCCHC3 is a novel anti-HIV-1 host factor that evades antagonization by viral proteins. Given that ZCCHC3 targets multiple steps of HIV-1 replication, it is important to test the therapeutic effect of its overexpression in cells persistently or latently infected with HIV-1. Furthermore, structural and biochemical investigations are required to design a modified ZCCHC3 with higher potency.

### Limitations of the study

There are several limitations in this study. First, we observed a marginal effect of *ZCCHC3* knockout in primary CD4^+^ T cells (Figure 1F). This may be due to the low basal expression of ZCCHC3 in primary CD4^+^ T cells (Figure 1A) or due to a yet unknown mechanism by which the virus may circumvent ZCCHC3 antiviral activity. It remains to be seen if overexpression of ZCCHC3 (through CRISPRa or other means of transcriptional upregulation) in primary CD4^+^ T cells will result in restriction, as seen in other models. Furthermore, the impact of the single nucleotide polymorphism (SNP) in the *Zcchc3* gene on the anti-HIV-1 activity in primary CD4^+^ T cells should be investigated in future studies. Second, another limitation of this study is that most of the experiments herein used an overexpression system to test the antiviral activity of ZCCHC3 (Figure 1B). However, the fact that mutant ZCCHC3 with G324S or Y328F substitutions did not show any antiviral activity suggests the specificity of the antiviral activity by WT ZCCHC3 (Figures 1D and S1B). Furthermore, our data showed that the ZCCHC3-containing virus showed significantly lower infectivity in monocyte-derived THP-1 cells (Figure S1D), suggesting that cell type may affect the antiviral activity of ZCCHC3.

## STAR★Methods

### Key resources table

**Table:** 

REAGENT or RESOURCE	SOURCE	IDENTIFIER
**Antibodies**		

mouse anti-HIV-1 p24 monoclonal antibody	HIV Reagent Program, NIH	Cat# ARP-3537; RRID: AB_3086785
horseradish peroxidase (HRP)-conjugated goat anti-mouse IgG antibody	KPL	Cat#074–1806; RRID: AB_2891080
HRP-conjugated goat anti-mouse IgG antibody	Sigma-Aldrich	Cat# 12-349; RRID: AB_390192
anti-HA antibody	Cell Signaling Technology	Cat# 2999; RRID: AB_1264166
anti-EGFP antibody	Medical & Biological Laboratories Co., Ltd.	Cat#598; RRID: AB_591819
HRP-conjugated sheep anti-mouse antibody	Cytiva	Cat# NA931V; RRID: AB_772210
HRP-conjugated goat anti-rabbit antibody	Thermo Fisher Scientific	Cat# A-11036; RRID: AB_10563566
anti-LSM14A rabbit polyclonal antibody	Atlas Antibodies	Cat# HPA017961; RRID: AB_1845610
anti-G3BP1 mouse monoclonal antibody	Santa Cruz Biotechnology	Cat# sc-365338; RRID: AB_10846950
anti-ZCCHC3 mouse monoclonal antibody	Sigma-Aldrich	Cat# SAB1408147; RRID: AB_10744359
Alexa Fluor 647 goat anti-mouse IgG	Abcam	Cat# ab150115; RRID: AB_2687948
Alexa Fluor 568 goat anti-rabbit IgG	Thermo Fisher Scientific	Cat#A-11036; RRID:AB_10563566
Alexa Fluor 488 goat anti-mouse IgG	Thermo Fisher Scientific	Cat# A-11034; RRID: AB_2576217
anti-p24 antibody	R&D	Cat# MAB7360; RRID: AB_10993570
anti-β actin antibody	Sigma-Aldrich	Cat# A5441; RRID: AB_476744
anti-CD3 (UCHT1) antibody	Tonbo Biosciences	Cat# 40-0038-U500; RRID: AB_2621439
anti-CD28 (CD28.2) antibody	Tonbo Biosciences	Cat# 40-0289-U500; RRID: AB_2621446
anti-ZCCHC3 antibody	Cell Signaling Technology	Cat# 65321; RRID: AB_3086784
anti-cGAS (D1DG3) antibody	Cell Signaling Technology	Cat# 15102S; RRID: AB_2732795
anti- mouse β-actin (8H10D10) antibody	Cell Signaling Technology	Cat# 3700; RRID: AB_2242334
Peroxidase AffiniPure Goat Anti-Rabbit IgG (H + L)	Jackson ImmunoResearch	Cat# 111-035-003; RRID: AB_2313567

**Bacterial and virus strains**		

HIV-1, Strain NL4-3 Infectious Molecular Clone (pNL4-3)	NIH HIV Reagent Program, Division of AIDS, NIAID, NIH	ARP-2852
HIV-1NL4-3 Nef-IRES-GFP plasmid	NIH HIV Reagent Program, Division of AIDS, NIAID, NIH	ARP-11349
pMSMnG	Gift from Dr. Jun-ichi Sakuragi	N/A
Panel of Full-Length Transmitted/Founder (T/F) HIV-1 Infectious Molecular Clones	NIH HIV Reagent Program, Division of AIDS, NIAID, NIH	ARP-11919
Human Immunodeficiency Virus Type 2 (HIV-2) ST Infectious Molecular Clone	NIH HIV Reagent Program, Division of AIDS, NIAID, NIH	ARP-12444
SIVcpzTAN 2.69 Infectious Molecular Clone	NIH HIV Reagent Program, Division of AIDS, NIAID, NIH	ARP-11497
SIVcpzTAN3.1 Infectious Molecular Clone	NIH HIV Reagent Program, Division of AIDS, NIAID, NIH	ARP-11498
SIVagmSab92018ivTF	NIH HIV Reagent Program, Division of AIDS, NIAID, NIH	ARP-12140
SIV Packaging Construct, SIV3+,	NIH HIV Reagent Program, Division of AIDS, NIAID, NIH	ARP-13456
SIV LTR Luciferase mCherry Reporter Vector	NIH HIV Reagent Program, Division of AIDS, NIAID, NIH	ARP-13455
pEIAV-SIN6.1 CGFPW	44171	Addgene
*Escherichia coli* strain BL21(DE3) CondonPlus RIL	Agilent Technologies, Inc.	230245
NEB 5-alpha F′Iq Competent *E. coli* (High Efficiency)	NEB, Ipswich	C2992H

**Chemicals, peptides, and recombinant proteins**		

KOD-Plus-Neo	TOYOBO, CO., ltd.	KOD-401
PrimerSTAR® Max DNA Polymerase	Takara Bio Inc.	R046A
Dulbecco’s Modified Eagle Medium (DMEM) high glucose	Nacalai Tesque	08468–16
penicillin/streptomycin	Corning	30-002-CI
DMEM with low glucose	Sigma-Aldrich	D6046
fetal bovine serum (FBS)	Sigma-Aldrich	173012
G418	Nacalai Tesque	16512–81
RPMI-1640	Sigma-Aldrich	R8758
2.5 g/L- trypsin/EDTA solution	Nacalai Tesque	32777–44
polyethylenimine hydrochloride (PEI)	Polyscience	49553-93-7
TransIT®-293 Transfection Reagent	Mirus Bio LLC	MIR2700
NuPAGE LDS sample buffer	Thermo Fisher Scientific	NP0007
Western BLoT Ultra Sensitive HRP Substrate	Takara	T7104A
Ni-NTA column	Fujifilm	141–09764
Glutathione Sepharose 4B-column	Cytiva	17127901
Amicon Ultra (M.W. 3,000 Da)	Sigma-Aldrich	UFC9003
protease inhibitor cocktail	Nacalai Tesque	25955–11
Chemi-Lumi One Super Kit	Nacalai Tesque	02230–14
RNase inhibitor	TOYOBO, CO., ltd.	SIN-201
Quanti Fluor RNA dye	Promega	E286A
goat serum	Cedarlane	CL1200-100
polyethene glycol	Sigma-Aldrich	P2139
Esp3I	NEB	R0734S
puromycin	Invivogen	ant-pr-1
SYBR™ Gold Nucleic Acid Gel Stain	Thermo Fisher Scientific	S11494
4X lenti-concentrator	OriGene	TR30025

**Critical commercial assays**		

Lenti-X p24 Rapid Titer Kit	Takara Bio Inc.	632200
Nano Glo HiBiT Lytic Detection System	Promega	N3040
TriFECTa® RNAi Kit	IDT	107099486
TransIT-X2 Dynamic Delivery System	Takara Bio Inc.	V6100
CellAmp Direct RNA Prep Kit for RT-PCR (Real Time)	Takara Bio Inc.	3732
One Step TB Green PrimeScript PLUS RT-PCR Kit (Perfect Real Time)	Takara Bio Inc.	RR096A
DNA Ligation Kit	Takara Bio Inc.	6023
RNeasy Mini Kit	QIAGEN	74104
QIAshredder	QIAGEN	79656
NucleoSpin® RNA Virus kit	Takara Bio Inc.	U0956A
MegaSscript T7 Transcription Kit	Ambion	AM1333
Bright-Glo Luciferase Assay System	Promega	E2620
ViewRNA® Cell Plus Assay kit	Thermo Fisher	QVC001
Ficoll-Paque	Cytiva	17-5442-03
EasySep™ Human CD4^+^ T cell Isolation Kit	STEMCELL Technologies	17952
4-(2-hydroxyethyl)-1-piperazineethanesulfonic acid (HEPES)	Corning	45000–692
Human IL-2	Miltenyi Biotec	130097744
Cas9	UC-Berkeley Macrolab	Cas9-NLS
P3 Nucleofector solution	Lonza	V4SP-3960
T cell Activation/Expansion Kit, human	Miltenyi Biotec	130-091-441
Polyethylene glycol	Sigma-Aldrich	81260-5KG
Criterion Precast Tris-HCl 4–20% gel	BioRad	3450033
ReBlot Plus mild antibody stripping solution	Millipore	2502
CellTiter-Glo luminescent cell viability assay reagent	Promega	G7570
Polyjet™ *In Vitro* DNA Transfection Reagent	SignaGen Laboratories	SL100688
Immun-Blot polyvinylidene difluoride (PVDF) Membrane	Millipore	1620177
RPMI-1640	Corning	10-040-CV
Sodium Pyruvate, Liquid 100 mM Solution	Corning	25-000-CI
HyClone™ Penicillin-Streptomycin Solution	Cytiva	SV30010

**Experimental models: Cell lines**		

TZM-bl cells	NIH HIV Reagent Program, Division of AIDS, NIAID, NIH	ARP-8129
Lenti-X 293T cells	Takara Bio Inc.	632180
COS7 cells	Riken BRC Cell Bank	0539
HEK293T	ATCC	CRL-3216
HeLa S3 cells	ATCC	CCL-2.2
HeLa CD4 cells	NIH AIDS reagent program	154
MT4 cells	Japanese Collection of Research Bioresources Cell Bank	JCRB1216
Peripheral Blood Leukopaks	STEMCELL Technologies	200–0092
Primary CD4^+^ T cells	STEMCELL Technologies	Isolated from peripheral blood leukopaks

**Oligonucleotides**		

primers	See Table S2 for the primers used in this study	N/A
RNA sequences	See Table S4 for the primers used in this study	N/A
lyophilized crRNA and tracrRNA	Dharmacon	N/A

**Recombinant DNA**		

pLionII	Addgene	1730
pCPRDEnv	Addgene	1732
pEV53D	Addgene	44168
lentiCRISPR v2	Addgene	52961
pMD2.G	Addgene	12259
psPAX2	Addgene	12260
psPAX2-IN/HiBiT and	Gift from Dr. Kenzo Tokunaga	N/A
pWPI-Luc2	Gift from Dr. Kenzo Tokunaga	N/A
pLV-eGFP	Addgene	36083
pGP	Takara Bio Inc.	6161
pDON-5 *Neo* DNA	Takara Bio Inc.	3657
pEGFP-C3	Takara Bio Inc.	PT3832-5
pCMV-HA-N2	Takara Bio Inc.	Z5690N
pET28a(+)	Takara Bio Inc.	N/A
pGEX6P1	Cytiva	27-1542-01
pmCherry-C1	Takara Bio Inc.	Z2524N
pBluescript II KS(−)	addgene	212207

**Software and algorithms**		

iBright Analysis Software v5.2	Thermo Fisher Scientific	https://www.thermofisher.com
FlowJo software (vX)	BD	https://www.flowjo.com
Proteome Discoverer version 2.5	Thermo Fisher Scientific	https://www.thermofisher.com
Prism 9 software v9.1.1	GraphPad Software Inc.	https://www.graphpad.com/
ImageJ (v 1.52q)	NIH	https://wsr.imagej.net/ij/
Adobe Illustrator 2021	Adobe	https://www.adobe.com/products/illustrator.html
Attune NxT Software v5.3.0	Thermo Fisher Scientific	https://www.thermofisher.com

**Other**		

LAS-3000 Imager	Fujifilm	https://www.biocompare.com
FLUOstar Omega Plate reader	BMG Labtech	https://www.bmglabtech.com
confocal laser scanning microscope FV3000	Olympus	https://www.olympus-lifescience.com
stage incubator	TOKAI HIT Corporation	https://www.tokaihit.com
flow cytometer, LSRFortessa	BD Biosciences	https://www.bdbiosciences.com
flow cytometer, Attune CytPix Flow Cytometer	Thermo Fisher Scientific	https://www.thermofisher.com
flow cytometer, Attune NxT acoustic focusing cytometer	Thermo Fisher Scientific	https://www.thermofisher.com
QuantStudio 5 Real-Time PCR System	Thermo Fisher Scientific	https://www.thermofisher.com
StepOne Plus Real-Time PCR System	Thermo Fisher Scientific	https://www.thermofisher.com
Orbitrap Fusion mass spectrometer	Thermo Fisher Scientific	https://www.thermofisher.com
GloMax Explorer Multimode Microplate Reader	Promega	https://www.promega.jp
SimpleWestern Abby	Protein Simple	http://www.bio-techne.com/instruments/simple-western

### Resource availability

#### Lead contact

Further information and requests for resources and reagents should be directed to and will be fulfilled by the lead contact, Shige H. Yoshimura (yoshimura@lif.kyoto-u.ac.jp).

#### Materials availability

All unique and stable reagents generated in this study are available from the lead contact with a completed materials transfer agreement.

#### Data and code availability

• All data reported in this paper will be shared by the lead contact upon request.
• This paper does not report original code.
• Any additional information required to reanalyze the data reported in this paper is available from the lead contact upon request.

### Experimental model and study participant details

#### Cell culture

TZM-bl cells (ARP-8129^32^^,^^33^^,^^34^^,^^35^^,^^36^^,^^37^) were obtained through the NIH HIV Reagent Program, Division of AIDS, NIAID, NIH (contributed by Dr. John C. Kappes, Dr. Xiaoyun Wu and Tranzyme Inc.) Lenti-X 293T cells (632180, Takara Bio Inc.), TZM-bl cells, HEK293T cells (CRL-3216, ATCC) and COS7 cells (0539, Riken BRC Cell Bank) were cultured in Dulbecco’s Modified Eagle Medium (DMEM) high glucose (08468-16, Nacalai Tesque, Kyoto, Japan), while HeLa S3 cells (CCL-2.2, ATCC) were cultured in DMEM with low glucose (D6046, Sigma-Aldrich, St. Louis, MO, USA), supplied with 10% (v/v) fetal bovine serum (FBS) (173012, Sigma-Aldrich). HeLa CD4^+^ cells (154, NIH AIDS reagent program) were maintained in DMEM supplemented with 10% (v/v) FBS and 2 mg/mL G418 (16512-81, Nacalai Tesque, Kyoto, Japan). MT4 cells (JCRB1216, Japanese Collection of Research Bioresources Cell Bank), C8166-CCR5 cells, and THP-1 cells were cultured in RPMI-1640 (R8758, Sigma-Aldrich) supplemented with 10% (v/v) FBS. For passaging the adherent cells, cells were treated with 2.5 g/L-trypsin/EDTA solution (32777-44, Nacalai Tesque). All the cells were incubated in a humidified incubator at 37°C with 5% CO_2_.

#### Isolation of primary CD4^+^ T cells

Primary CD4^+^ T cells from healthy human donors were isolated from peripheral blood leukopaks (STEMCELL Technologies). Peripheral blood mononuclear cells (PBMCs) were isolated by Ficoll-Paque (Cytiva) centrifugation, followed by CD4^+^ T cell isolation using an EasySep Human CD4^+^ T cell Isolation Kit according to manufacturer’s instructions (STEMCELL Technologies). Cells were resuspended at a density of 2.5×10^6^ cells/mL in complete Roswell Park Memorial Institute (RPMI) media consisting of RPMI-1640 (Corning) media with 5 mM 4-(2-hydroxyethyl)-1-piperazineethanesulfonic acid (Corning), 50 μg/mL penicillin/streptomycin (Corning), 5 mM sodium pyruvate (Corning), and 10% fetal bovine serum (Gibco) and supplemented with 20 IU/mL IL-2 (Miltenyi Biotec) immediately prior to use. Cells were plated 500 μL per well in stimulation plates made by coating 48 well plates with 20 mg/mL anti-CD3 antibody (UCHT1, Tonbo Biosciences) diluted in Dulbecco’s Phosphate Buffer Saline (DPBS, Corning) overnight at 4°C, with soluble anti-CD28 (CD28.2, Tonbo Biosciences) added at 5 mg/mL to the cell suspension at the time of plating. Cells were stimulated for 72 h in a cell culture incubator at 37°C with 5% CO_2_ prior to CRISPR-Cas9 editing.

### Method details

#### Materials

The following reagents were obtained through the NIH HIV Reagent Program, Division of AIDS, NIAID, NIH: HIV-1, Strain NL4-3 Infectious Molecular Clone (pNL4-3) (ARP-2852^54^) contributed by Dr. M. Martin, Panel of Full-Length Transmitted/Founder (T/F) HIV-1 Infectious Molecular Clones (ARP-11919^55^^,^^56^) contributed by Dr. John C. Kappes, Human Immunodeficiency Virus Type 2 (HIV-2) ST Infectious Molecular Clone (ARP-12444) contributed by Dr. Beatrice Hahn and Dr. George Shaw, SIVcpzTAN 2.69 Infectious Molecular Clone (ARP-11497^57^) and SIVcpzTAN3.1 Infectious Molecular Clone (ARP-11498^57^) contributed by Drs. Jun Takehisa, Matthias H. Kraus and Beatrice H. Hahn, SIVagmSab92018ivTF (ARP-12140) contributed by Drs. Frank Kirchhoff, Clement Gnanadurai, and Beatrice Hahn, and SIV Packaging Construct (SIV3+, ARP-13456) and SIV LTR Luciferase mCherry Reporter Vector (ARP-13455) contributed by Dr. Tom Hope. psPAX2-IN/HiBiT and pWPI-Luc2 were kind gifts from Dr. Kenzo Tokunaga.^58^ pMSMnG was a kind gift from Dr. Jun-ichi Sakuragi.^59^ pLionII (1730; http://n2t.net/addgene:1730; RRID: Addgene_1730) and pCPRDEnv (1732; http://n2t.net/addgene:1732; RRID: Addgene_1732) were gifts from Dr. Garry Nolan. pEIAV-SIN6.1 CGFPW (44171; http://n2t.net/addgene:44171; RRID: Addgene_44171) and pEV53D (44168; http://n2t.net/addgene:44168; RRID: Addgene_44168) were gifts from Dr. John Olsen. lentiCRISPR v2 was a gift from Feng Zhang (Addgene plasmid# 52961; http://n2t.net/addgene:52961; RRID: Addgene_52961).^60^ pMD2.G (12259; http://n2t.net/addgene:12259; RRID: Addgene_12259) and psPAX2 (12260; http://n2t.net/addgene:12260; RRID: Addgene_12260) were gifts from Dr. Didier Trono. pLV-eGFP was a gift from Dr. Pantelis Tsoulfas (36083; http://n2t.net/addgene:36 083; RRID: Addgene_36083).^61^ pGP (# 6161) and pDON-5 Neo DNA (# 3657) were purchased from Takara Bio Inc. (Shiga, Japan). The luciferase-encoding and ZsGreen-encoding retroviral vectors were described previously.^62^

#### DNA constructions

The cDNA encoding human ZCCHC3 (NM_033089.7) was amplified from cDNA pool of HeLa cells by PCR and cloned into pEGFP-C3 (PT3832-5, Takara Bio Inc., Shiga, Japan), pCMV-HA-N2 (Takara Bio Inc.), pET28a(+) (Takara Bio Inc.) or pGEX6P1 (Cytiva, Marlborough, MA, USA). Fragments of the ZCCHC3 N-terminus (a.a. 1–159), ZCCHC3 C terminus (a.a. 159–404), ZCCHC3 MF fragment (a.a. 159–334) and ZCCHC3 ZnF fragment (a.a. 334–404) were amplified by PCR using KOD-Plus-Neo (KOD-401, TOYOBO, CO., ltd., Osaka, Japan), and subcloned into pEGFP-C3 and pCMV-HA-N2 for mammalian expression. Fragments of ZCCHC3 C terminus (a.a. 159–404) and ZCCHC3 MF fragment (a.a. 159–334) were amplified by PCR and subcloned into pET28a(+), for ZCCHC3 MF fragment also pGEX6P1 for expression in *Escherichia coli*.

The ZCCHC3 MF fragments carrying amino acid substitution(s) were generated by replacing the amino acids of R164, R168, R210, R247, K253, R289, K298 or K329 with S, respectively; the R268SR247S mutation was generated by replacing the amino acid of R168 and R247 with S. Single, and double amino acid mutations were introduced using PrimerSTAR Max DNA Polymerase (R046A, Takara Bio Inc.). The ZCCHC3 expression vectors carrying missense mutations in the human *Zcchc3* gene were generated using PrimerSTAR Max DNA Polymerase (#R046A, Takara Bio Inc.). The primers used for the mutation generation were listed in Table S2.

The whole Gag sequence was amplified from wild-type Gag plasmid by PCR and cloned into pGEX6P1 and pmCherry-C1 (Takara Bio Inc.). Fragments of MAp17 (a.a. 1–132), CAp24 (a.a. 133–363), Gag NCp7 (a.a. 378–432) and p6 (a.a. 449–500) were amplified by PCR using KOD-Plus-Neo and subcloned into pGEX6P1 for expression in *Escherichia coli*.

The DNA fragments encoding 5′-LTR (454–634 nt, GenBank: MN989412.1) and Gag protein (1–181 nt) of HIV-1 were amplified by PCR using KOD-Plus-Neo and pNL4-3 as a template and cloned into pBluescript II KS(−) (212208, Agilent). The MLV 5′-LTR (1–207 nt, GenBank: KU324804.1) sequence was amplified from pDON-5 *Neo*, the EIAV 5′ LTR (1–114 nt, GenBank: AF247394.1) was amplified from pEIAV-SIN6.1 CGFPW, and SIV 5′ LTR (1–351 nt, GenBank: DQ374657.1) was amplified from SIVcpzTAN 2.69 and cloned into pBluescript II KS(−).

To generate reporter constructs, HIV-1 5′-LTR (FL) (377–634 nt, GenBank: MN989412.1), HIV-1 5′-LTR (R-U5) region (377–634 nt, GenBank: MN989412.1), a coding region of *gag* (3023–3203 nt, GenBank: MN989412.1), β-actin promoter on pCAGGS (386–661 nt) were amplified by PCR using KOD-Plus-Neo. The R-U5 fragment was cloned into NheI/AgeI or XhoI/EcoRI sites of pEGFP-C1 to generate CMV-LTR-EGFP or CMV-EGFP-LTR, respectively. The gag fragment was cloned into NheI/AgeI sites of pEGFP-C1 to generate CMV-gag-EGFP. The LTR (FL) fragment or the β-actin promoter fragment was cloned into AseI/NheI sites of pEGFP-C1 to generate LTR-EGFP or Pactin-EGFP, respectively. Information on all the primers and restriction sites for cloning is summarized in Table S2.

#### Plasmid transfection, virus production and collection, virus infection

Plasmid DNAs were introduced into Lenti-X 293T or HEK293T cells using either polyethyleneimine hydrochloride (PEI) (49553-93-7, Polyscience, Niles, IL, USA) or TransIT-293 Transfection Reagent (MIR2700, Mirus Bio LLC, Madison, WI, USA). For production of VSV-G-pseudotyped HIV-1, Lenti-X 293T cells were co-transfected with pMSMnG and pMD2.G plasmids. The supernatant was collected and filtered 48 h after transfection. For production of lentivirus, a transfer vector (pLV-EGFP or pWPI-Luc2) was introduced into HEK293T or Lenti-X 293T cells together with a packaging vector (psPAX2 or psPAX2-IN/HiBiT) and an envelope vector (pMD2.G) at a ratio of 5:3:2. The culture medium was collected 48 h after the transfection and centrifuged at 1,500 g for 10 min at 4°C to remove cell debris. For other plasmid transfections, PEI was used.

Both lentiviral and retroviral vectors were rescued as described previously in the presence or absence of pCMV-HA-ZCCHC3 plasmids. To rescue a SIVmac-based lentiviral vector, Lenti-X 293T cells were co-transfected with the pSIV3+ plasmid, SIV LTR Luciferase mCherry Reporter Vector, and pMD2.G plasmid. To rescue an FIV-based lentiviral vector, Lenti-X 293T cells were co-transfected with the pCPRDEnv, pLionII-luc2, and pMD2.G plasmids. To rescue an EIAV-based lentiviral vector, Lenti-X 293T cells were co-transfected with pEV53D, EIAV-SIN6.1-luc2, and pMD2.G plasmids. To rescue an MLV-based retroviral vector, Lenti-X 293T cells were co-transfected with pGP, pDON-5 Neo-luc2, and pMD2.G plasmids. The supernatant was collected and filtered 48 h after transfection.

Expression and processing of Gag proteins in Lenti-X 293T cells were evaluated with western blot. Lenti-X 293T cells transfected with either TF2625, pNL4-3 or pMSMnG were washed and lysed in 1× NuPAGE LDS sample buffer (NP0007, Thermo Fisher Scientific) containing 2% (v/v) β-mercaptoethanol and incubated at 70°C for 10 m. The incorporation of HA-tagged ZCCHC3 in the virions was evaluated using SimpleWestern Abby (ProteinSimple, San Jose, CA, USA) with an anti-HA tag mouse monoclonal antibody (1: 50; Cell Signaling Biotechnology, Danvers, MA, USA, Cat# 2367S) and an anti-mouse detection module (ProteinSimple, Cat# DM-001). HIV-1 Gag expression was evaluated with anti-HIV-1 p24 mouse monoclonal antibody (1: 500; clone 183-H12-5C, ARP-3537, obtained from the HIV Reagent Program, NIH)^63^ and an Anti-Mouse Detection Module. The amount of input protein was visualized using a total protein detection module (ProteinSimple, Cat# DM-TP01).

#### Purification of recombinant protein

Plasmids encoding hexahistidine (Hisx6)- and glutathione S-transferase (GST)-tagged proteins were introduced into *Escherichia coli* strain BL21(DE3) CondonPlus RIL (230245, Agilent Technologies, Inc.), and the expression of the recombinant protein was induced by 0.5 mM isopropylthio-β-D-galactoside (IPTG) in Luria Bertani broth at 18°C for 6 h. The Hisx6-fused protein was purified from the cell lysate by Ni-NTA column (141–09764, Fujifilm, Tokyo, Japan) and dialyzed by 200 mM NaCl, 50 mM HEPES, 1 mM β-mercaptoethanol for 6 h at 4°C. The GST-fused proteins were purified by Glutathione Sepharose 4B-column (17127901, Cytiva) and dialyzed by 200 mM NaCl, 50 mM HEPES, 7:100000 (V/V) β-mercaptoethanol (99%) for 6 h at 4°C. The proteins were concentrated by Amicon Ultra (M.W. 3,000 Da) (UFC9003, Sigma-Aldrich) and stored at −80°C. All the recombinant proteins were treated with RNase during bacteria cell lysate preparation.

#### Protein pull-down assay

HEK293T cells were cultured in DMEM to 80% confluency, harvested by centrifugation at 500*g* for 3 min, and re-suspended with PBS (pH 7.4) containing 1% (v/v) protease inhibitor cocktail (25955-11, Nacalai Tesque, Kyoto, Japan) 0.25% (w/v) Triton X-100 and incubated on ice for 10 min. The insoluble fraction was removed by centrifugation (1,500 g, 5 min), and the supernatant was collected and treated with RNase at on ice for 10 min. For pull-down assay using GST-fusion proteins, the purified GST-tagged Gag fragments (∼5 μg) were mixed with the lysate of HEK293T cells expressing HA- or EGFP-tagged proteins (ZCCHC3 fragments) and glutathione Sepharose 4B in Pull-down buffer (PBS (pH 7.4), 1 mM DTT), and incubated for 30 min at 25°C with gentle rotation. The beads were washed, and the bound proteins were eluted with 200 mM glutathione in Pull-down buffer, mixed with SDS-PAGE sample buffer (100 mM Tris-HCl (pH 6.8), 4% (w/v) SDS, 20% (v/v) glycerol, 0.15 mg/mL bromophenol blue), and heated for 5 min. The proteins were analyzed via SDS-PAGE using 12% (w/v) acrylamide gel and subjected to western blot. HA- and EGFP-tagged proteins were incubated with anti-HA antibody (1: 2,000; 2999, Cell Signaling Technology, Danvers, MA, USA) or anti-EGFP antibody (1: 2,000; 598, Medical & Biological Laboratories Co., Ltd., Nagano, Japan), followed by the incubation with secondary antibody (HRP-conjugated goat anti-mouse antibody (1: 10,000; NA931V, Cytiva) or HRP-conjugated goat anti-rabbit antibody (1: 10,000; A-11036, Thermo Fisher Scientific, Waltham, MA, USA). The immunoreactive bands were visualized using Chemi-Lumi One Super Kit (02230-14, Nacalai Tesque) under LAS-3000 Imager (Fujifilm). The gel was also stained with Coomassie Brilliant Blue (CBB) to check the input amount and the unbound fraction.

#### RNA and DNA pull-down

GST-tagged proteins were immobilized to glutathione Sepharose beads (Cytiva) and incubated with synthesized RNA for 10 min in RNA Pull-down buffer (20 mM Tris-HCl (pH 7.4), 30 mM NaCl, 0.1 mM MgCl_2_, 1 mM DTT, 1:100 RNase inhibitor (SIN-201, TOYOBO, CO., ltd., Osaka, Japan) in RNase-free water). The protein-RNA complex was eluted with 200 mM glutathione in RNA Pull-down buffer. The RNA amount in the eluted fraction was quantified with Quanti Fluor RNA dye (E286A, Promega, Madison, WI, USA) by following the manufacturer’s instructions. The eluted fraction was also subjected to SDS-PAGE, followed by CBB staining. For DNA pull-down assay, the DNA fragments were amplified by PCR using a plasmid carrying HIV-1 5′ LTR (454–634 nt, GenBank: MN989412.1), MLV 5′ LTR (1–207 nt, GenBank: KU324804.1) or Gag (935–1115 nt, GenBank: MN989412.1) as a template and the primers described in Table S2. Single-stranded DNAs were generated by heating the dsDNA at 95°C for 5 min, and quickly chilled on ice. The dsDNA and ssDNA were used in the pull-down assay instead of RNA as described above.

#### Immunofluorescence microscopy and confocal microscopy

Cells were fixed with 4% (w/v) paraformaldehyde (PFA) in PBS (pH 8.0) for 15 min and incubated with 5% (v/v) goat serum (CL1200-100, Cedarlane, Burlington, ON, Canada), in PBS (pH 8.0) containing 0.25% (w/v) Triton X-100 for 15 min. The following primary antibodies were used: anti-LSM14A rabbit polyclonal antibody (1: 500; HPA017961, Atlas Antibodies, Stockholm, Sweden), anti-p24 mouse monoclonal antibody (1: 500; MAB7360, R&D, Minneapolis, MN, USA), anti-G3BP1 mouse monoclonal antibody (1: 500; sc-365338, Santa Cruz Biotechnology, CA, USA) and anti-ZCCHC3 mouse monoclonal antibody (1: 500; SAB1408147, Sigma-Aldrich). The following secondary antibodies were used: Alexa Fluor 647 goat anti-mouse IgG (1: 1,000; ab150115, Abcam, Cambridge, UK), Alexa Fluor 568 goat anti-rabbit IgG (1: 1,000; A-11036, Thermo Fisher Scientific) and Alexa Fluor 488 goat anti-mouse IgG (1: 1,000; A-11034, Thermo Fisher Scientific, Waltham, MA, USA). Nuclei were stained with DAPI. For the cells expressing fluorescence protein-tagged proteins, the cells were fixed with 4% (w/v) PFA in PBS (pH 8.0) and stained with DAPI. The cells were observed using a confocal laser scanning microscope (FV-3000, Olympus, Tokyo, Japan) with a 100× objective lens (NA 1.42., Olympus). The obtained images were analyzed with ImageJ (v 1.52q, NIH, Bethesda, MD, USA). A stage incubator (TOKAI HIT Corporation, Shizuoka, Japan) was used for live-cell imaging; all observations were performed at 37°C and 5.0% CO_2_.

#### Phase separation assay

EGFP-tagged recombinant protein of ZCCHC3 ZnF and mCherry-tagged recombinant protein of HIV-1 Gag NC were used for the phase separation assay. The purified protein (∼10 mg/mL) was diluted to 3 mg/mL with the following buffer: 5 mM Tris-HCl (pH 8.0), 1.2 mM ATP, 1.1 mM DTT, 50 mM KCl, 2 mM MgCl_2_, and 10% (w/v) polyethyene glycol (P2139, Sigma-Aldrich). The droplet was observed using a fluorescence microscope (FV3000).

#### Purification of virus from culture medium by centrifugation

Cells were separated from the culture medium using low-speed centrifugation (700 *g*, 5 min, 4°C). The cell lysate was prepared as described in the previous section (protein pull-down assay). For concentrating the virion from the culture medium, the medium was layered onto 20% (w/v) sucrose in PBS (pH 7.4) layer and centrifuged at 20,380 *g* for 2 h at 4°C. After removing the supernatant, the pellet was re-suspended with ice-cold lysis buffer (PBS containing 1% (v/v) protease inhibitor cocktail (25955-11, Nacalai Tesque) 0.25% (w/v) Triton X-100, pH 7.4). The lysates (cell and virus) were subjected to SDS-PAGE (12% (w/v) acrylamide gel) and western blot analyses using anti-EGFP antibody (1: 2,000; A-11036, Medical & Biological Laboratories Co., Ltd.), anti-p24 antibody (1:500; MAB7360, R&D), and anti-β actin antibody (1:5,000; A5441, Sigma-Aldrich).

#### Flow cytometry

HeLa and TZM-bl cells were fixed with 4% (w/v) PFA in PBS (pH 8.0) for 15 min at 25°C. The cells were washed three times and re-suspended with PBS (pH 7.4). After filtration through nylon mesh, the cells were analyzed using a flow cytometer (LSRFortessa, BD Biosciences or Attune CytPix Flow Cytometer, Thermo Fisher Scientific). Data were analyzed using FlowJo software (vX). To confirm the expression of HA-tagged ZCCHC3, the cell lysate was subjected to SDS-PAGE and western blot analyses using anti-HA antibody (1:2,000, 2999S, Cell Signaling Technology).

#### p24 ELISA

The culture medium of Lenti-X 293T or HEK293T cells was harvested and centrifuged at 1,500 *g* for 10 min to remove cell debris. The p24 amount in the medium was quantified by Lenti-X p24 Rapid Titer Kit (632200, Takara Bio Inc.) with the p24 control samples, following the manufacturer’s instructions.

#### HiBiT assay

For culture supernatant of Lenti-X 293T cells transfected with the psPAX2-IN/HiBiT plasmid, the HiBiT value was measured 2 days after transfection using the Nano Glo HiBiT Lytic Detection System (N3040, Promega) as described previously.^52^ The HiBiT value was converted to p24 value based on a standard curve generated with a HiBiT-containing lentiviral vector whose p24 level was already determined by p24 ELISA.

#### *ZCCHC3* depletion in Lenti-X 293T cells

To deplete *ZCCHC3*, Lenti-X 293T cells adjusted to 1.25 × 10^6^ cells per well in a 6-well plate were transfected with TriFECTa RNAi Kit (hs.Ri.ZCCHC3.13, REF#: 107099486, IDT, Coralville, IA, USA) or non-targeting control siRNA with TransIT-X2 Dynamic Delivery System (V6100, Takara Bio Inc.) in Opti-MEM. After overnight culture, the cells were re-plated on a new 96-well plate at 2.5 × 10^4^ cells per well. The cells were cultured again overnight and subjected to the quantification of mRNA by qRT-PCR using the CellAmp Direct RNA Prep Kit for RT-PCR (Real Time) (3732, Takara Bio Inc.), One Step TB Green PrimeScript PLUS RT-PCR Kit (Perfect Real Time) (RR096A, Takara Bio Inc.), and primer pairs for *ZCCHC3* (5′-CTCTCTATGCCTTCTTAAACCGA-3′ and 5′-CATCTGCACGCTACAGTTCT-3′) and *ACTB* (5′-ACAGAGCCTCGCCTTTG-3′ and 5′-C CTTGCACATGCCGGAG-3′). qRT-PCR was performed using the QuantStudio 5 Real-Time PCR System (Thermo Fisher Scientific), and the Ct values of *ZCCHC3* were normalized to the mean values obtained using *ACTB* as a housekeeping gene (ΔΔCt method).

#### CRISPR-Cas9 editing of primary CD4^+^ T cells

CRISPR-Cas9 ribonucleoprotein complexes (crRNPs) were synthesized according to previously published protocols.^64^ Briefly, lyophilized crRNA and tracrRNA (Dharmacon) were resuspended at 160 μM in a buffer of 10 mM Tris-HCl (7.4 pH) and 150 mM KCl. To form crRNPs, 10 μL of 160 μM crRNA was mixed with 10 μL of 160 μM tracrRNA and incubated for 30 min at 37°C. For the cGAS pooled guide condition, equal volumes of five crRNA targeting cGAS were mixed prior to addition of tracrRNA. 20 μL of 40 μM Cas9 (UC-Berkeley Macrolab) was gently mixed with the resulting crRNA:tracrRNA complexes, then incubated at 37°C for 15 min crRNPs were aliquoted into ten sets of 3.5 μL each and stored at −80°C prior to use. crRNA were sourced from Dharmacon, either as custom sequences or from the Dharmacon pre-designed Edit-R library as shown in the Table S2.

Immediately prior to electroporation, activated primary CD4^+^ T cells were removed from stimulation plates and pooled per donor. 1x10^6^ cells per electroporation reaction were centrifuged at 400 g for 5 min, and supernatant was removed by aspiration. Cell pellets were resuspended in electroporation buffer consisting of 16.4 μL of P3 Nucleofector solution with 3.6 μL supplement per condition (Lonza). 20 μL of cell suspension was mixed with 3.5 μL of each crRNP and transferred to 96-well electroporation cuvettes for nucleofection with the 4D 96-well shuttle unit (Lonza) using pulse code EH-115. 100 μL of warm complete RPMI was added to cells post-electroporation, and cells recovered in a cell culture incubator at 37°C for 30 min. Cells were then moved to 96-well flat bottom tissue culture plates prefilled with 100 μL complete RPMI supplemented with 40 IU/mL IL-2 and 2.5 μL stimulation beads (Miltenyi Biotec) per well. Cells were cultured for 4 days prior to downstream assays to allow for protein turnover. Additional complete media with IL-2 was added to cells on day 2 post-electroporation.

#### Virus preparation and infection of primary CD4^+^ T cells

An HIV-1NL4-3 molecular clone with GFP cloned behind an IRES cassette following the viral nef gene (NEF-IRES-GFP) (NIH AIDS Reagent Program, #11349) was used for infection of primary CD4^+^ T cells. Viral stocks of 5×10^6^ human embryonic kidney 293T (HEK293T) cells (ATCC, CRL-3216) were transfected with 10 μg of HIV-1NL4-3 Nef-IRES-GFP plasmid DNA using 30 μL PolyJet transfection reagent according to manufacturer protocols (SignaGen Laboratories). Cell supernatants were harvested at days 2 and 3 post-transfection, pooled, and filtered through 0.45-mm polyvinylidene difluoride (PVDF) filters (Millipore). Virus was precipitated in 8.5% polyethylene glycol (Sigma-Aldrich) and 0.3 M NaCl for 6 h at 4°C then centrifuged at 3,500 rpm for 20 min at 4°C. Virus was resuspended in 250 μL DPBS for 100x effective concentration, then stored at −80°C prior to infection assays.

CRISPR-edited primary CD4^+^ T cells were plated 1×10^5^ cells per well in 96 well U bottom plates in 200 μL complete RPMI supplemented with IL-2 at 20 IU/mL. Cells were infected with an equivalent of 0.2 ng p24 of HIV-1NL4-3 NEF-IRES-GFP stock per well diluted in 50 μL complete RPMI with 20 IU/mL IL-2. Cells were incubated in a cell culture incubator at 37°C with 5% CO2. At days 2 and 5 post-infection, 75 μL of cell suspension was removed from each well, transferred to a new plate, and fixed with 75 μL of 2% formaldehyde (Fisher Scientific) in DPBS for later analysis by flow cytometry. 75 μL additional complete RPMI with IL-2 was added to each well and cultures were returned to incubator. Infection rates were analyzed by flow cytometry using Attune NxT acoustic focusing cytometer (Thermo Fisher Scientific). Samples were subjected to one 150 μL mixing cycle, and all events in 50 μL of sample were recorded. FCS3.0 files were exported using Attune NxT Software v5.3.0 and analyzed using a FlowJo template for HIV-1 infection (GFP, BL-1), excluding autofluorescence cells with equal fluorescence in BL-1 and VL-2 channels.

#### Knockout validation and viability assay in primary CD4^+^ T cells

To generate protein lysates, 1×10^5^ cells per condition were spun down on day 4 post-electroporation, washed with PBS, lysed in 50 μL 2.5x Laemmli sample buffer, and heated at 98°C for 20 min prior to storage at −20°C. For Western blotting, 15 μL of sample was loaded into Criterion Precast Tris-HCl 4–20% gels (Bio-Rad) alongside 10 μL PageRuler Plus Prestained ladder (Thermo Scientific). Gels were run at 90 V for 30 min, then 150 V for 70 min. Methanol-based electrotransfer was used to transfer proteins to PVDF membranes at 90 V for 120 min. Blots were blocked in 4% milk or 5% bovine serum albumin (BSA) in PBS with 0.1% Tween 20 (PBST), then incubated overnight with ZCCHC3 (1:1000, #65321, Cell Signaling Technology), cGAS (1:1000, Clone D1DG3, Cell Signaling Technology), or mouse β-actin (1:10000, 8H10D10, Cell Signaling Technology) primary antibody at 4°C with rocking. Blots were washed in PBST and incubated with anti-rabbit or anti-mouse HRP-conjugated antibodies (1:10000, Jackson ImmunoResearch) diluted in 4% milk in PBS with 0.1% Tween 20. Blots were washed in PBST, incubated with chemiluminescent HRP substrate reagent (EMD Millipore), then imaged on an iBright imaging system. Blots were stripped using ReBlot Plus mild antibody stripping solution (EMD Millipore), re-blocked, and incubated with the next antibody in the series.

Viability of CRISPR-edited cells was assessed using CellTiter-Glo luminescent cell viability assay reagents according to manufacturer’s instructions (Promega). Luminescence was measured using a BMG FLUOstar Omega Plate reader.

#### Generation of *ZCCHC3* knockout cells

LentiCRISPRv2 plasmids (52961, Addgene) targeting *ZCCHC3* gene were generated as follows. The following oligos (100 pmol) were mixed and heated at 95°C for 5 min, followed by incubation at room temperature for 1 h for annealing, with 5′-caccgCCTGTTCCTACGCGTCTACG-3′ and 5′-aaacTAACCTCTCGGAGCCTCTGCc-3′ for sgRNA#1, and 5′-caccgAGGGCGAATTCCGCGAGCCG-3′ and 5′-aaacCGGCTCGCGGAATTCGCCCTc-3′ for sgRNA#2. The mixture was 250-fold diluted with water and used for ligation with the lentiCRISPRv2 plasmid, which was predigested with Esp3I (R0734 S, NEB). The solution was mixed with DNA Ligation Kit <Mighty Mix> (6023, Takara Bio Inc.) and used for transformation with NEB 5-alpha F′Iq Competent *E. coli* (High Efficiency) (C2992H, NEB, Ipswich, MA, USA). After the miniprep, the nucleotide sequence of the plasmid was verified by nucleotide sequencing using a primer (5′-GAGGGCCTATTTCCCATGATT-3′).

The lentiCRISPRv2-*ZCCHC3*-sgRNA#1 or lentiCRISPRv2-*ZCCHC3*-sgRNA#2 plasmids were used for co-transfection with psPAX2-IN/HiBiT and pMD2.G plasmids on Lenti-X 293T cells using TransIT-293 Transfection Reagent. The culture supernatant was collected 2 days after transfection and used for infection on Lenti-X 293T cells. The cells were cultured for 2 weeks in the presence of 1 μg/mL puromycin (ant-pr-1, InvivoGen). The cells were single-cell-cloned using a limiting dilution method. The presence or absence of a ZCCHC3 specific band with each clone was determined by western blotting using an anti-human ZCCHC3 antibody (SAB1408147, Sigma-Aldrich).

#### Reverse transcription quantitative PCR

Lenti-X 293T cells were co-transfected with pNL4-3 with or without pCMV-HA-ZCCHC3 plasmid. At 2 days after transfection, the total RNA was extracted from cells using an RNeasy Mini Kit (74104, QIAGEN, Hilden, Germany) and QIAshredder (79656, QIAGEN, Hilden, Germany), and subjected to RT-qPCR for quantification with the primer pairs for Gag-coding region (5′-TGTAATACCCATGTTTTCAGCA-3′ and 5′-TCTGGCCTGGTGCAATAGG-3′) and *ACTB* (5′-TCCAAATATGAGATGCGTTGTT-3′ and 5′-TGCTATCACCTCCCCTGTGT-3′). The qPCR was performed using the StepOne Plus Real-Time PCR System (Thermo Fisher Scientific). The Ct values of Gag-coding region were normalized to the mean values obtained using *ACTB* as a housekeeping gene (ΔΔCt method).

For quantifying lentiviral RNA in the virion released from the producer cells to the culture medium, RNA was purified from the medium with NucleoSpin RNA Virus kit (U0956A, Takara Bio Inc.) by following the manufacturer’s instruction and subjected to RT-qPCR with the primer pairs for EGFP (5′-CAAGCTGACCCTGAAGTTCATCTG-3′ and 5′-TTGAAGAAGTCGTGCTGCTTCATG-3′) and U5-coding region (5′-TCTGGCTAACTAGGGAACCCACTG-3′ and 5′-ACTGCTAGAGATTTTCCACACTGAC-3′). The PCR mix (15 μL) contained One step TB Green RT-PCR kit (RR096A, Takara Bio Inc.), each primer, 2 μL of nucleic acid samples and water. The primers were presented at a final concentration of 400 nM. The qPCR was performed using the StepOne Plus Real-Time PCR System (Thermo Fisher Scientific).

#### Preparation of viral RNA

The plasmid carrying HIV-1 5′ LTR (454–634 nt, GenBank: MN989412.1), MLV 5′ LTR (1–207 nt, GenBank: KU324804.1), EIAV 5′ LTR (1–114 nt, GenBank: AF247394.1), SIV 5′ LTR (1–351 nt, GenBank: DQ374657.1) and Gag (935–1115 nt, GenBank: MN989412.1) under T7 promotor was linearized at the 3′ end of the insert, and used as a template in *in vitro* transcription reaction (MegaSscript T7 Transcription Kit (AM1333, Ambion, Waltham, MA, USA). The synthesized RNA was purified by isopropanol precipitation and quantified by measuring OD 260 nm. Short stem-loop RNA molecules derived from HIV-1 LTR (SL-1, 2, and 3) were synthesized by FasMac. The sequences were as follows: SL1, 5′-UCUCUGGUUAGACCAGAUCUGAGCCUGGGAGCUCUCUGGCUAACUAGGGA-3’; SL2, 5′-CCACUGCUUAAGCCUCAAUAAAGCUUGCCUUGAGUGCUCAAAGUAGUGU-3’; and SL3, 5′-CUAGAGAUCCCUCAGA CCCUUUUAGUCAGUGUGGAAAAUCUCUAG-3’.

#### Electrophoretic mobility shift assay (EMSA)

ssRNA, dsDNA, and ssDNA were prepared as described in the previous section (RNA and DNA pull down). Synthesized nucleic acids (0.1 pmol) were incubated with purified proteins (0, 0.1, 0.2, 1 pmol) in EMSA binding buffer (40 mM Tris (pH 8.0), 2 mM KCl, 1 mM MgCl_2_, 1% (w/v) NP-40, 1 mM DTT in RNase-free water) at 25°C for 10 min. The samples were separated by electrophoresis using 3.5% (w/v) acrylamide/bisacrylamide gel in TBE (100 mM Tris-base, 100 mM boric acid, 2 mM EDTA), and visualized with SYBR Gold Nucleic Acid Gel Stain (S11494, Thermo Fisher Scientific).

#### Stable cell line generation and BioID analysis

HEK293T cells were co-transfected with pDON-5 Neo-TurboID tagged ZCCHC3, pGP and pMD2.G at the ratio of 2:1:1 by PEI. The culture medium was collected 48 h after transfection, centrifuged at 1,500 g for 10 min at 4°C to remove cell debris, and added to the culture medium of HeLa cells. G418 was added to the medium at a final concentration of 100 μg/mL 48 h after infection. Cells were diluted to a 96-well-plate at the concentration of 1–5 cells per well. Positive wells were screened using a confocal laser scanning microscope (FV-3000, Olympus) The cells were infected by lentivirus produced as described in the previous section (Plasmid transfection, virus production and collection, virus infection) for 30 min before labeling. For labeling with biotin, the cells were incubated with 500 mM biotin for 30 min. The cells were rinsed with 5 mL ice-cold HEPES-saline (20 mM HEPES-NaOH, pH 7.5, 137 mM NaCl). Another 2 mL HEPES-saline was added to the dish to collect the cells with scraping. The cells were harvested by centrifugation at 800*g* for 3 min at 4°C and re-suspended with 500 μL guanidine-TCEP buffer (8 M guanidine, 100 mM HEPES-NaOH, pH 7.5, 10 mM tris(2-carboxyethyl)phosphine hydrochloride, 40 mM chloroacetamide). Proteins were extracted and digested with trypsin followed by enrichment of biotinylated peptides as described previously.^65^ LC-MS/MS analysis of the biotinylated peptides was performed on an EASY-nLC 1200 UHPLC connected to an Orbitrap Fusion mass spectrometer (Thermo Fisher Scientific) as described previously.^65^ Raw data were directly analyzed against the SwissProt database restricted to *Homo sapiens* using Proteome Discoverer version 2.5 (Thermo Fisher Scientific) with the Sequest HT search engine. The search parameters were as follows: (a) trypsin as an enzyme with up to two missed cleavages; (b) precursor mass tolerance of 10 ppm; (c) fragment mass tolerance of 0.6 Da; (d) carbamidomethylation of cysteine as a fixed modification; and (e) acetylation of protein N-terminus, oxidation of methionine, and biotinylation of lysine as variable modifications.

#### Luciferase assay

Cells infected with a luciferase-encoding virus were lysed 2 days after infection with a Bright-Glo Luciferase Assay System (E2620, Promega), and the luminescent signal was measured using a GloMax Explorer Multimode Microplate Reader (Promega).

#### FISH (fluorescence in situ hybridization)

HeLa CD4^+^ cells were plated on poly-L-lysine pre-treated cover glass and incubated for overnight before infection or transfection. The HIV-1 lentivirus was produced as described previously, then centrifuged at 1,500 *g* for 10 min at 4°C to collect the supernatant. The supernatant was mixed with the 4X lenti-concentrator (TR30025, OriGene, MD, USA) at a ratio of 1:4 for O/N at 4°C. The supernatant was removed by centrifugation at 3,500 *g* for 25 min and 5 min at 4°C. The pellet was re-suspended with PBS (pH 7.4). The pre-transfected HeLa CD4^+^ cells were fixed by 4% of PFA 30 min after infected with lentivirus. The ViewRNA Cell Plus Assay kit (QVC001, Thermo Fisher) was used for the next steps. Cells were stained with antibodies and probes (VF6-13336, Thermo Fisher) following the manufacturer’s instruction. The samples were observed by a confocal laser scanning microscope (FV-3000, Olympus) with a 100× NA1.42 objective lens.

#### Protein structure predictions

The three-dimensional protein structure of human ZCCHC3 (NM_033089.7) was predicted using ColabFold (v.1.5.2: AlphaFold2 using MMseqs2) (https://colab.research.google.com/github/sokrypton/ColabFold/blob/main/AlphaFold2.ipynb).

#### Protein-RNA binding predictions

The protein-RNA binding was predicted busing catRAPID omics (v2.0) (http://s.tartaglialab.com/page/catrapid_omics2_group). The amino acids sequence of ZCCHC3, the nucleotide sequences of HIV-1 LTR RU5 region and MLV LTR used for the prediction could be found in NCBI (ZCCHC3: NM_033089.7; HIV-1 LTR RU5 region: MN989412.1; and MLV LTR: KU324804.1).

### Quantification and statistical analysis

The total number of independent experiments in each analysis is described in the figure legends. All statistical analyses were evaluated by an unpaired, two-tailed Student’s *t* test unless otherwise indicated. p ≤ 0.05 were considered statistically significant. The tests were performed using Prism 9 software v9.1.1 (GraphPad Software Inc., Boston, MA, USA).

## Supplemental information

Document S1. Figures S1–S5 and Table S4

Table S1. Raw data of proximity-dependent biotin identification of proteins (BioID analysis) using TurboID-fused ZCCHC3. Worksheets labeled “experiment 1–3” show 3 independent experiments, related to Fig. 6

Table S2. Primers used in this study, related to STAR methods

Table S3. catRAPID prediction of the interaction between ZCCHC3 protein and the HIV-1 genome (NC_001802.1), related to Fig. S4A

Data S1. Raw images of western blots and microscopic images, related to Figure 1

Data S2. Raw images of western blots and microscopic images, related to Figure 2

Data S3. Raw images of western blots and microscopic images, related to Figure 3

Data S4. Raw images of western blots and microscopic images, related to Figure 4

Data S5. Raw images of western blots and microscopic images, related to Figure 5

Data S6. Numerical data of all the Figures and Supplementary Figures in this article
