# Supplementary material for: Host ZCCHC3 blocks HIV-1 infection and production through a dual mechanism
Source: iScience. 2024 Feb 5;27(3):109107. doi: 10.1016/j.isci.2024.109107 (PMC10879702; doi:10.1016/j.isci.2024.109107)
Supplement: Document S1. Figures S1–S5 and Table S4 [file mmc1.pdf]

**iScience, Volume *27***

## Supplemental information

# Host ZCCHC3 blocks HIV-1

# infection and production through a dual mechanism

**Binbin Yi, Yuri L. Tanaka, Daphne Cornish, Hidetaka Kosako, Erika P. Butlertanaka, Prabuddha Sengupta, Jennifer Lippincott-Schwartz, Judd F. Hultquist, Akatsuki Saito, and Shige H. Yoshimura**

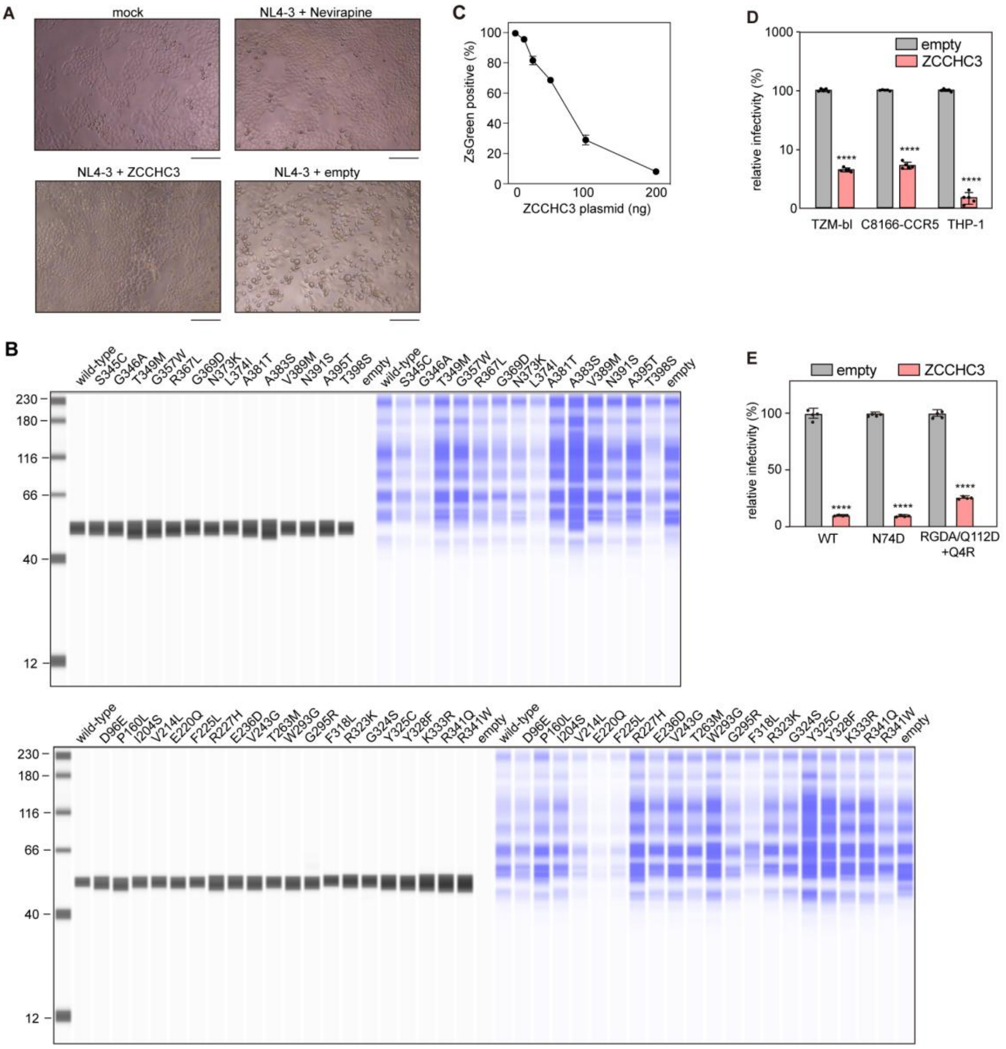
A
mock
NL4-3 + Nevirapine
NL4-3 + ZCCHC3
NL4-3 + empty
B
wild-type S345C G346A T349M G357W R367L G369D N373K L374I A381T A383S V389M N391S A395T T398S empty wild-type S345C G346A T349M G357W R367L G369D N373K L374I A381T A383S V389M N391S A395T T398S empty
230
180
116
66
40
12
wild-type D96E P160L I204S V214L E220Q F225L R227H E236D V243G T263M W293G G295R F318L R323K G324S Y325C Y328F K333R R341Q R341W empty wild-type D96E P160L I204S V214L E220Q F225L R227H E236D V243G T263M W293G G295R F318L R323K G324S Y325C Y328F K333R R341Q R341W empty
230
180
116
66
40
12
C
ZsGreen positive (%)
100
80
60
40
20
0
0 100 200
ZCCHC3 plasmid (ng)
D
empty
ZCCHC3
relative infectivity (%)
1000
100
10
0
****
****
****
TZM-bl
C8166-CCR5
THP-1
E
empty
ZCCHC3
relative infectivity (%)
100
50
0
****
****
****
WT
N74D
RGDA/Q112D
+Q4R

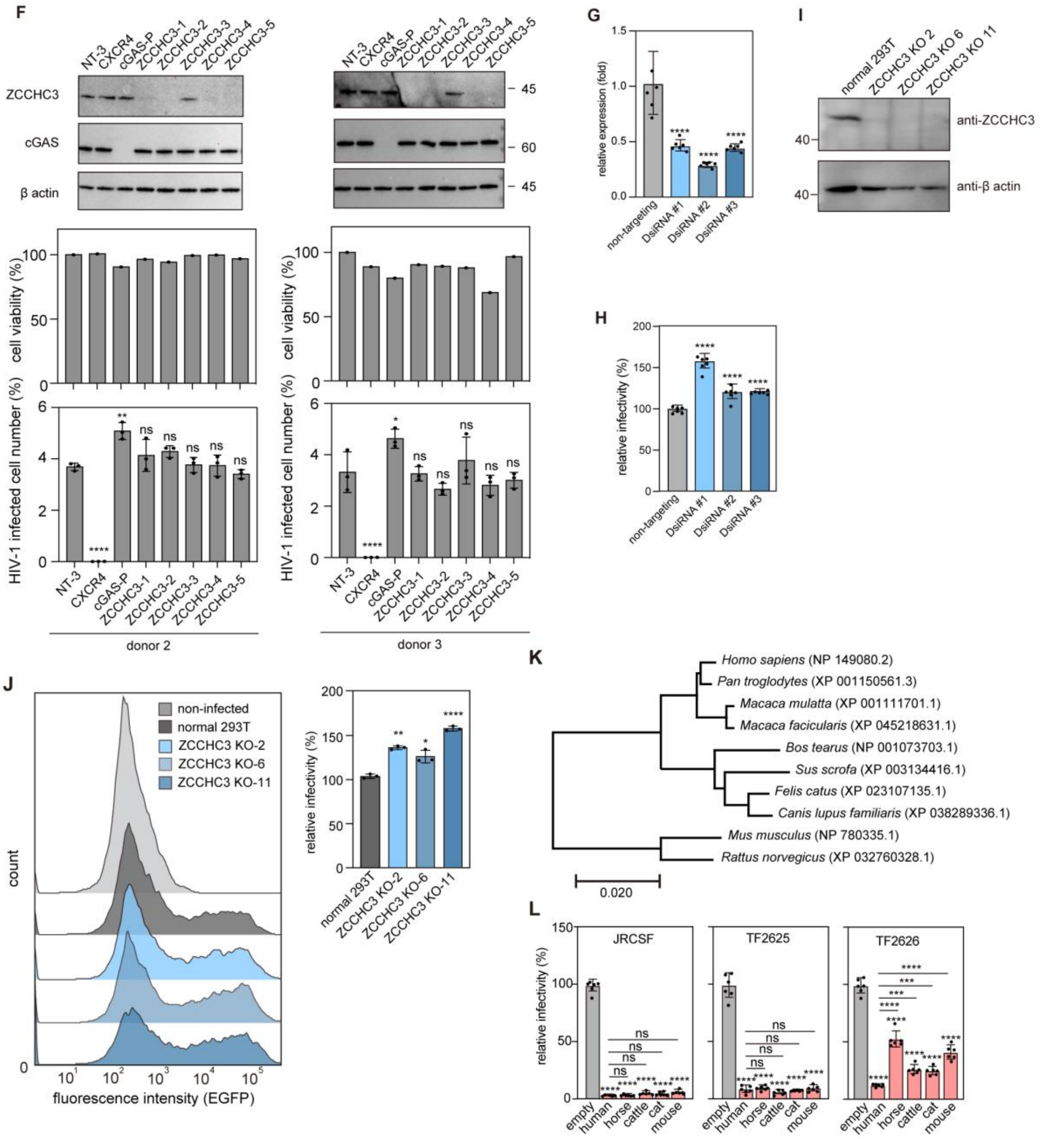

F
NT-3
CXCR4
cGAS-P
ZCCHC3-1
ZCCHC3-2
ZCCHC3-3
ZCCHC3-4
ZCCHC3-5
ZCCHC3
cGAS
β actin
45
60
45
cell viability (%)
HIV-1 infected cell number (%)
donor 2
donor 3
G
relative expression (fold)
non-targeting
DsiRNA #1
DsiRNA #2
DsiRNA #3
H
relative infectivity (%)
I
normal 293T
ZCCHC3 KO 2
ZCCHC3 KO 6
ZCCHC3 KO 11
anti-ZCCHC3
anti-β actin
J
non-infected
normal 293T
ZCCHC3 KO-2
ZCCHC3 KO-6
ZCCHC3 KO-11
count
fluorescence intensity (EGFP)
K
Homo sapiens (NP 149080.2)
Pan troglodytes (XP 001150561.3)
Macaca mulatta (XP 001111701.1)
Macaca facicularis (XP 045218631.1)
Bos tearus (NP 001073703.1)
Sus scrofa (XP 003134416.1)
Felis catus (XP 023107135.1)
Canis lupus familiaris (XP 038289336.1)
Mus musculus (NP 780335.1)
Rattus norvegicus (XP 032760328.1)
0.020
L
JRCSF
TF2625
TF2626
empty
human
horse
cattle
cat
mouse

**Figure S1 Additional data on the effect of ZCCHC3 on viral infection, related to Figure 1.** **A**, TZM-bl cells were infected with HIV-$1_{NL4-3}$ viruses produced in the presence or absence of an HA-ZCCHC3 expression plasmid. The cells were observed by optical microscopy 2 days after infection. TZM-bl cells infected with HIV-$1_{NL4-3}$ in the presence of Nevirapine served as a control (n =1). Scale bar, 50 μm. **B**, Effect of SNPs in human *ZCCHC3* gene on viral infectivity of retroviral vectors. A western blot analysis of the ZCCHC3 expression level in the transfected cells together with CBB staining. **C**, Dose-dependent suppression of lentiviral infectivity by ZCCHC3. Lenti-X 293T cells were co-transfected with EGFP-expressing lentiviral vectors together with different amounts of HA-ZCCHC3 expression vector. Culture supernatant was collected 2 days after transfection and used to infect MT4 cells. Cell fluorescence was measured by flow cytometry 2 days after infection. The mean and standard deviation values from quadruplicate measurements are shown. **D**, Antiviral effect of ZCCHC3 is Env-independent. pMSMnG plasmid was introduced into Lenti-X 293T cells with or without a ZCCHC3 expression plasmid. VSV-G-pseudotyped viruses were collected 2 days after transfection and used to infect the indicated cells. Infectivity on TZM-bl cells was determined as in (Figure 1C); otherwise, it was determined by flow cytometry as a percentage of GFP-positive cells 2 days after infection. Values relative to those for cells harboring empty vector are shown as the mean ± standard deviation (n = 5). **E**, Sensitivity of CA mutants to ZCCHC3. pMSMnG plasmid encoding the indicated HIV-1 CA variant was introduced into Lenti-X 293T cells with or without a ZCCHC3 expression plasmid. Culture supernatant was collected 2 days after transfection and used to infect C8166-CCR5 cells. Infectivity was determined by flow cytometry as in (Figure S1B). Values relative to those for cells harboring empty vector are shown as the mean ± standard deviation (n = 6). **F**, Effect of *ZCCHC3* knockout on viral infection in primary $CD4^+$ T cells. The experiment shown in Figure 1F were conducted for another two donors and are presented in the same manner. **G**, Knockdown of *ZCCHC3*. *ZCCHC3* mRNA in Lenti-X 293T cells transfected with DsiRNA targeting *ZCCHC3* or control DsiRNA was quantified by RT-qPCR. The mean and standard

deviation values are shown (n = 6). **H**, Effect of *ZCCHC3* knockdown on viral infectivity on target cells. Lenti-X 293T cells transfected with DsiRNA targeting *ZCCHC3* or control DsiRNA were infected with a lentiviral vector encoding the luciferase reporter protein. Infectivity was quantified as relative light units of luciferase and is presented as the mean and standard deviation (n = 6). **I**, Western blot analysis of *ZCCHC3*-KO cells. Three KO cell lines were analyzed by western blotting using an anti-ZCCHC3 antibody, with β-actin as a loading control. A representative image from three independent experiments is shown. **J**, Infectivity of lentivirus produced from normal and *ZCCHC3*-KO cells, analyzed by flow cytometry (left). Positive cells were counted (right). The mean and standard deviation values are shown from three independent experiments. **K**, Phylogenetic tree of mammalian ZCCHC3 proteins. The tree was constructed using the maximum-likelihood method. **L**, Anti-HIV-1 effect of mammalian ZCCHC3. A plasmid encoding the indicated virus was introduced into Lenti-X 293T cells with or without mammalian HA-ZCCHC3 expression plasmid. Culture supernatant was prepared, and TZM-bl cells were infected and analyzed as in (Figure 1C). Values relative to that of human ZCCHC3 are shown as the mean and standard deviation (n = 6). In **D**, **E**, **J**, differences were examined by a two-tailed, unpaired Student's *t*-test; ****$p < 0.0001$, **$p < 0.01$, *$p < 0.05$. In **F**, **G**, **H** and **L** differences were examined by one-way ANOVA, followed by Tukey's test; ****$p < 0.0001$, ***$p < 0.001$, **$p < 0.01$, * $p < 0.05$ ns $p \geq 0.05$.

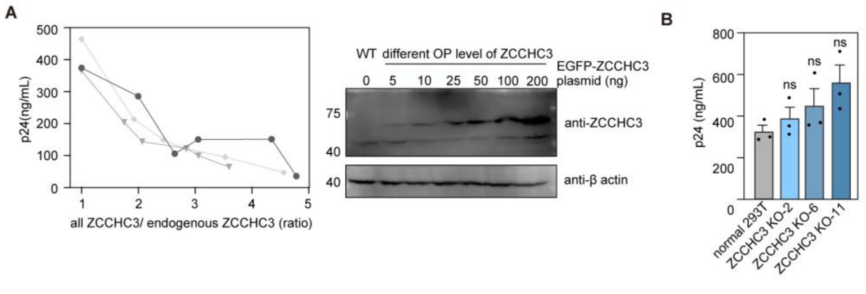


**Figure S2 Additional data on the effect of ZCCHC3 on viral production, related to Figure 2. A**, The inhibitory effect of ZCCHC3 on viral production is dose-dependent. HEK293T cells were transfected with different amounts of ZCCHC3-encoding plasmid together with a constant amount of lentiviral vectors. The culture medium was subjected to p24 ELSA 2 days after the transfection. The cell lysate was also subjected to western blot analysis using anti-ZCCHC3 to quantify endogenous and exogenous ZCCHC3. The p24 amount was plotted against the total ZCCHC3 amount for three independent experiments (left panel). A representative western blot image of ZCCHC3 expression level is shown (right panel). **B**, The amount of HIV-1 released into the culture medium was quantified in three different *ZCCHC3*-KO cell lines by p24 ELISA. The mean and standard deviation values from three independent experiments are shown. Differences were examined by a two-tailed, unpaired Student's $t$-test. ****$p < 0.0001$, ***$p < 0.001$, *$p < 0.05$; ns, $p \geq 0.05$.

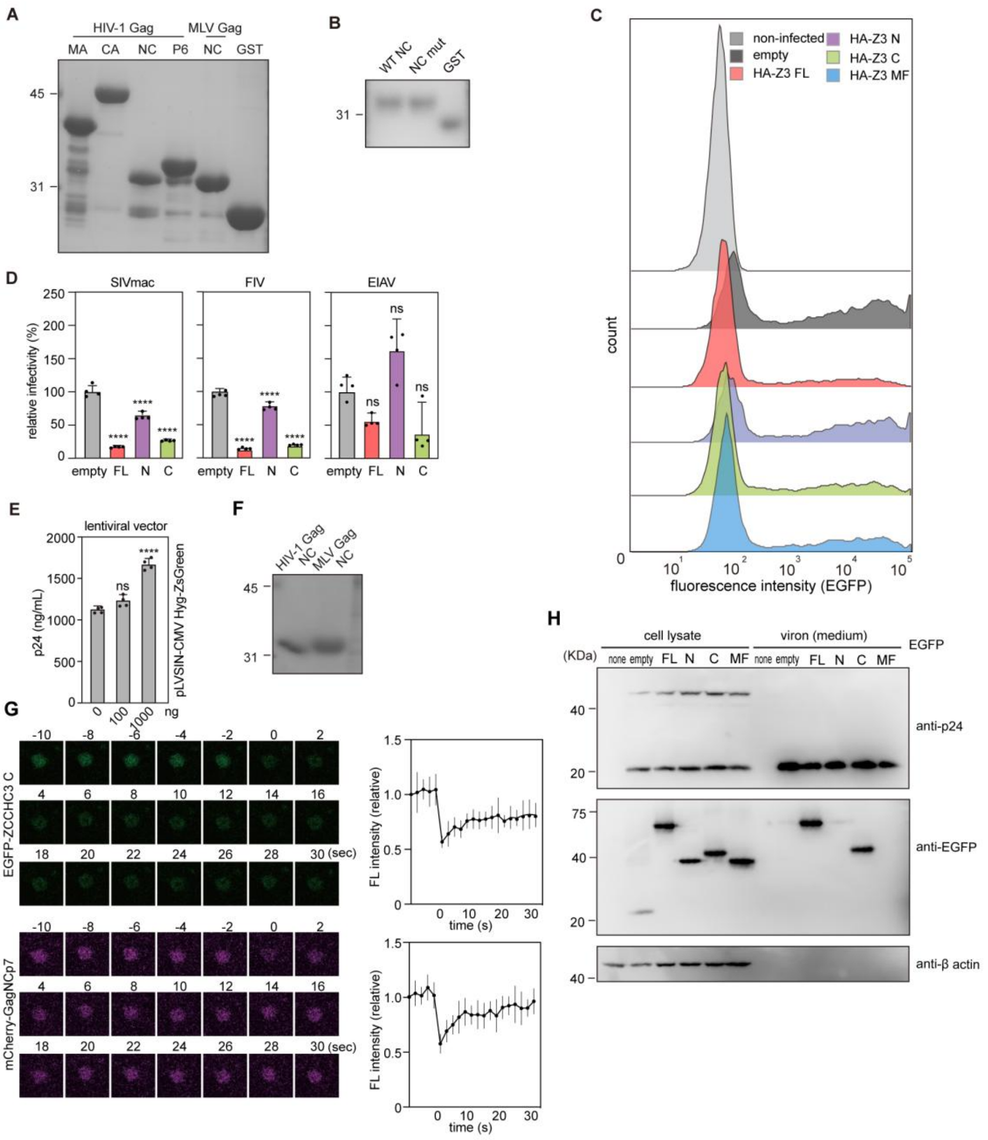

A
HIV-1 Gag
MLV Gag
MA CA NC P6 NC GST
45
31
B
WT NC
NC mut
GST
31
C
non-infected
empty
HA-Z3 FL
HA-Z3 N
HA-Z3 C
HA-Z3 MF
count
fluorescence intensity (EGFP)
D
SIVmac
FIV
EIAV
relative infectivity (%)
empty FL N C
E
lentiviral vector
p24 (ng/mL)
pLVSIN-CMV Hyg-ZsGreen
0 100 1000 ng
F
HIV-1 Gag NC
MLV Gag NC
45
31
G
EGFP-ZCCHC3 C
mCherry-GagNCp7
FL intensity (relative)
time (s)
H
cell lysate
viron (medium)
EGFP
(KDa)
none empty FL N C MF none empty FL N C MF
anti-p24
anti-EGFP
anti-β actin

**Figure S3 Additional data on ZCCHC3 binding to GagNC via C-terminal ZnFs, related to Figure 4. A**, The CBB staining showing the amount of the bait protein of Figure 3A. **B**, The CBB staining showing the amount of the bait protein of Figure 3D. **C**, Changes of lentiviral infectivity upon ZCCHC3 loading. HeLa cells were infected with a p24-normalized amount of lentiviruses carrying a ZCCHC3 fragment (FL, N, C, or MF). Infectivity was analyzed based on the expression of a viral gene (EGFP) using flow cytometry. **D**, ZCCHC3 C fragment suppresses retroviral infection. Lenti-X 293T cells were co-transfected with plasmids for generating SIVmac, FIV, or EIAV lentiviral vectors in the presence of a ZCCHC3 fragment (FL, N, or C), and the resultant viruses were used to infect MT4 cells. Infectivity was determined as relative light units 2 days after infection. Values relative to those of cells without ZCCHC3 expression are shown as the mean ± standard deviation (n = 5). **E**, Lenti-X 293T cells were co-transfected with 1000 ng of psPAX2-IN/HiBiT plasmid and different amounts of pLVSIN-CMV Hyg-ZsGreen vector. p24 concentration (converted from HiBiT value) in the culture supernatant was determined 2 days after transfection. Values are shown as the mean ± standard deviation (n = 4). **F**, The CBB staining showing the amount of the bait protein of Figure 4E. **G**, FRAP analysis of a condensate formed by GagNCp7 and the ZCCHC3 C fragment, as shown in Figure 4F. The fluorescence signal of the condensate was bleached at time 0, and the fluorescence intensity in the bleached area was monitored through time-lapse imaging. Data are shown as the mean (bold solid line) and standard deviation (vertical lines) (n = 10). **H**, Incorporation of different ZCCHC3 fragments into the lentiviral virion. A plasmid encoding EGFP-tagged ZCCHC3 FL, N, C, or MF was introduced into HEK293T cells together with lentiviral plasmids (pLV-SIN-Hyg, psPAX2, pMD2.G). Virions released into the culture medium were harvested by centrifugation and analyzed by immunoblotting with anti-GFP, anti-p24, and anti-β-actin antibodies. In **D** and **E**, differences were examined by one-way ANOVA, followed by Tukey's test. ****$p < 0.0001$; ns, $p \geq 0.05$.

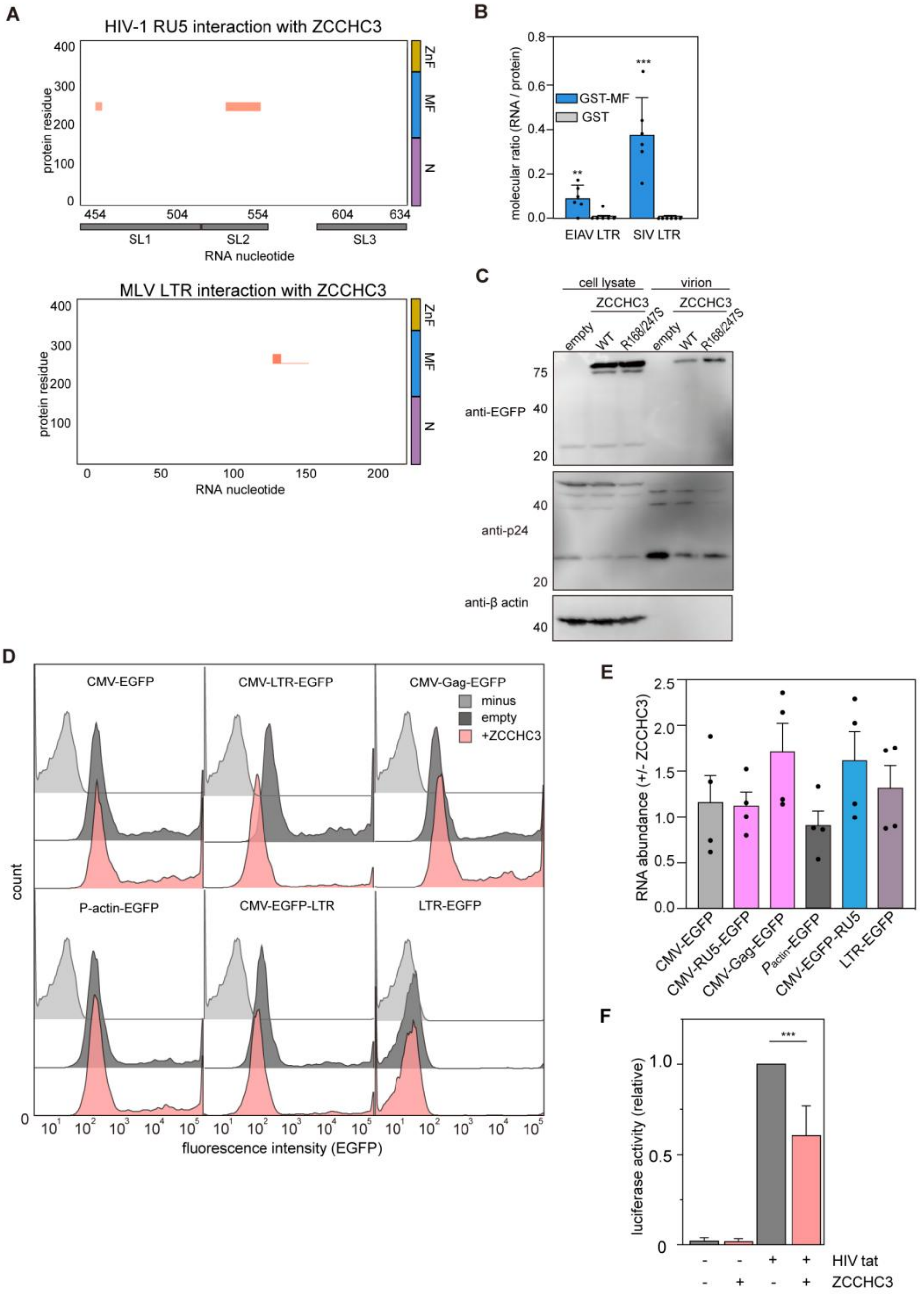
A
HIV-1 RU5 interaction with ZCCHC3
protein residue
400
300
200
100
0
454
504
554
604
634
SL1
SL2
SL3
RNA nucleotide
ZnF
MF
N
MLV LTR interaction with ZCCHC3
protein residue
400
300
200
100
0
50
100
150
200
RNA nucleotide
B
molecular ratio (RNA / protein)
GST-MF
GST
**
***
EIAV LTR
SIV LTR
C
cell lysate
virion
ZCCHC3
empty
WT
R168/247S
anti-EGFP
anti-p24
anti-β actin
75
40
20
D
CMV-EGFP
CMV-LTR-EGFP
CMV-Gag-EGFP
minus
empty
+ZCCHC3
P-actin-EGFP
CMV-EGFP-LTR
LTR-EGFP
count
fluorescence intensity (EGFP)
E
RNA abundance (+/- ZCCHC3)
CMV-EGFP
CMV-RU5-EGFP
CMV-Gag-EGFP
Pactin-EGFP
CMV-EGFP-RU5
LTR-EGFP
F
luciferase activity (relative)
***
HIV tat
ZCCHC3

**Figure S4 Additional data on ZCCHC3 binding to retroviral RNA, related to Figure 5.** **A**, The binding propensity of ZCCHC3 to HIV1-1 LTR (R-U5) RNA sequence and MLV LTR sequence, predicted by catRAPID. The nucleotide numbers of HIV-1 (GenBank: MN989412.1) and MLV genomes (GenBank: KU324804.1) are plotted on the x-axis. The HIV-1 SL1, SL2, and SL3 positions are depicted along the x-axis. ZCCHC3 N, MF, and ZnF positions are depicted along the y-axis. **B**, ZCCHC3 MF domain binding to EIAV and SIV LTR RNA. RNA pull-down assay was performed using the ZCCHC3 MF fragment and EIAV LTR and SIV LTR, as in Figure 4B. The mean and standard deviation values from three independent experiments. **C,** R168/247S mutations failed to affect incorporation into the virion and suppression of the viral production. Lentiviral plasmids (pLV-EGFP, psPAX2, and pIIIenv3-1) were introduced into HEK293T cells with or without the plasmid encoding EGFP-ZCCHC3 WT or R168/247S mutant. Virions were harvested via centrifugation, and analyzed using immunoblotting with anti-ZCCHC3, anti-p24, and anti-β-actin antibodies. **D**. Representative traces of the reporter genes expression level by flow cytometry (Figure 5G). **E** The amount of cellular mRNA in the cells described in Fig. 5G. The RNA amount was quantified by RT-qPCR, and presented as the ratio of with (+) and without (-) the expression of ZCCHC3. The results are presented as the mean and standard deviation of four independent experiments. **F**, Effect of ZCCHC3 on viral gene expression in a proviral state. TZM-bl cells carrying a luciferase gene flanked by 5′- and 3′-LTRs were transfected with a plasmid encoding HIV-1 Tat with or without HA-ZCCHC3. Luciferase activity in the cell lysate was then measured. The data are presented as the mean ± standard deviation from four independent experiments. In **B, E,** and **F,** differences were examined by a two-tailed, unpaired Student's *t*-test. ***$p < 0.001$, **$p < 0.01$, *$p < 0.05$; ns, $p \geq 0.05$.

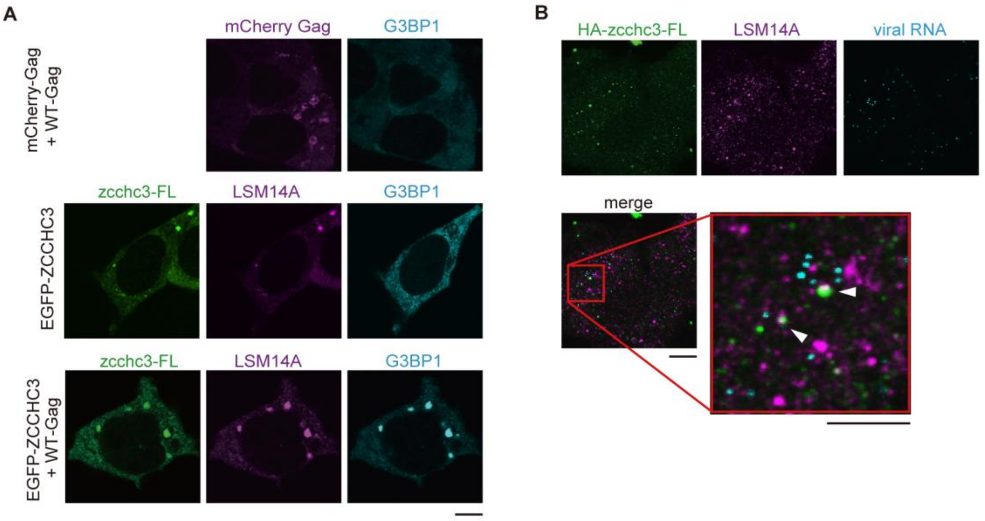


**Figure S5 Additional data of ZCCHC3 sequestration of viral RNA in P-body, related to Figure 6. A**. G3BP1 co-localized with ZCCHC3 and LSM14A. HEK293T cells were co-transfected with a plasmid encoding WT-Gag and mCherry-Gag (top) or EGFP-ZCCHC3 (middle and bottom), and the bottom panel was also transfected with WT Gag. The cells were immuno-stained with anti-LSM14A and anti-G3BP1 antibodies. Representative images are shown. Scale bar, 5 µm. **B**. FISH-immunostaining of lentivirus-infected cells. HA-ZCCHC3-expressing HeLa CD4+ cells were infected with lentivirus and immuno-stained with anti-LSM14A followed by FISH using the probe against HIV-1 Gag gene. Representative images are shown. Scale bar, 5 µm (25 µm for enlarged image).

Table S4. Sequence information for the three stem-loop structures within the HIV-1 LTR. Related to Fig. 5.

| name | sequence |
| --- | --- |
| SL1 | UCUCUGGUUAGACCAGAUCUGAGCCUGGGAGCUCUCUGGCUAACUAGGGA |
| SL2 | CCACUGCUUAAGCCUCAAUAAAGCUUGCCUUGAGUGCUCAAAGUAGUGU |
| SL3 | CUAGAGAUCCCUCAGACCCUUUUAGUCAGUGUGGAAAAUCUCUAG |
