# Supplementary material for: Host ZCCHC3 blocks HIV-1 infection and production through a dual mechanism
Source: iScience. 2024 Feb 5;27(3):109107. doi: 10.1016/j.isci.2024.109107 (PMC10879702; doi:10.1016/j.isci.2024.109107)

Data S1: Raw images of western blots and microscopic images, related to Figure 1.

Figure 1A

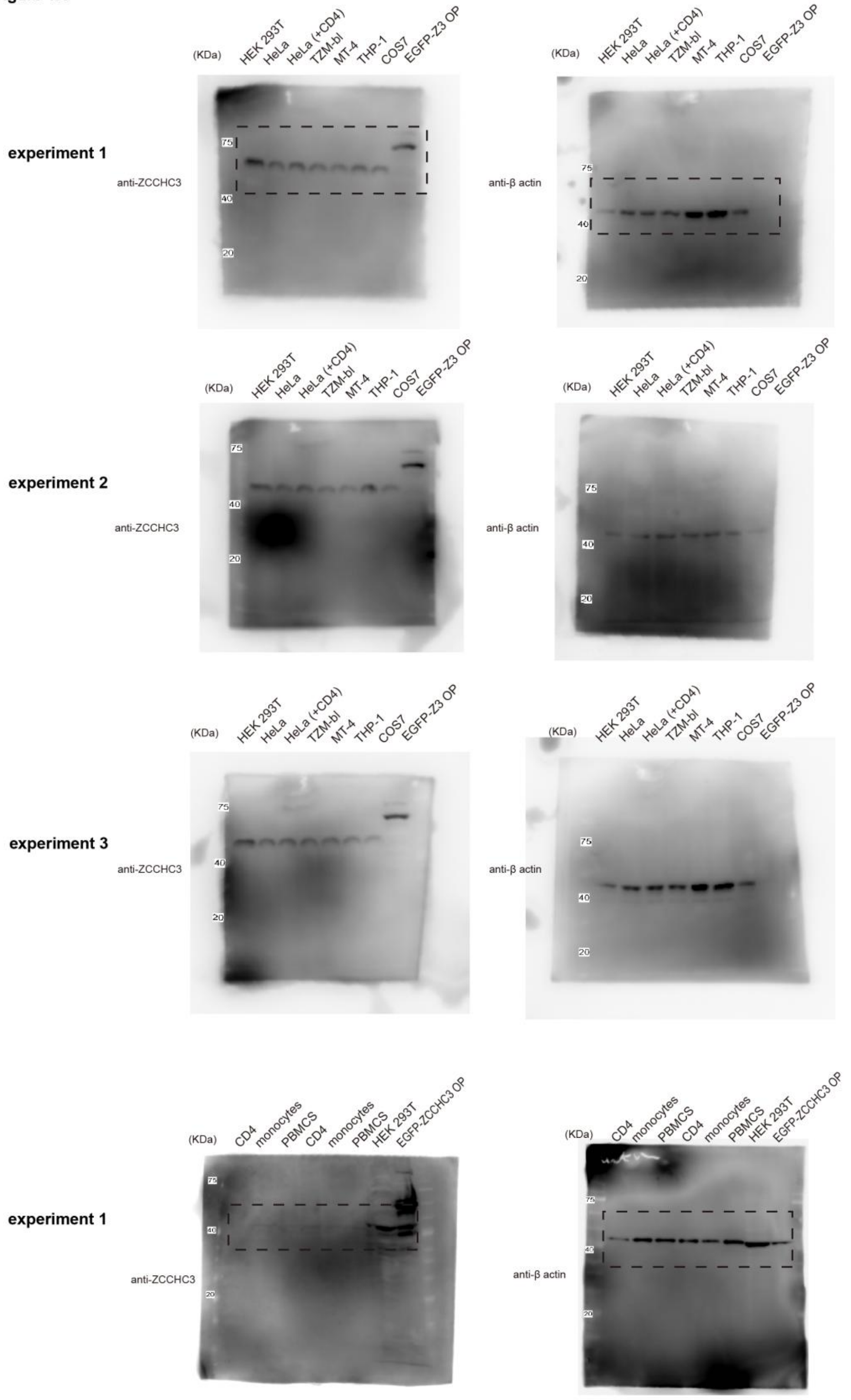

Figure 1F and S1F

donor 2 and 1

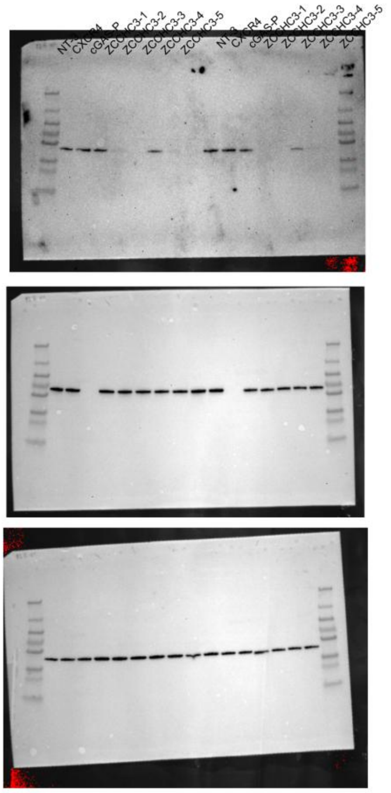

donor 3

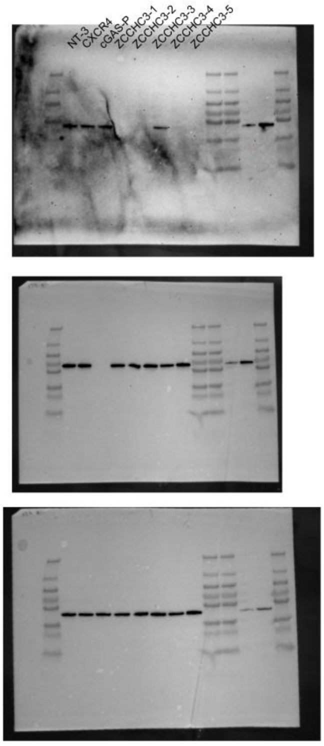

Figure S1A

mock

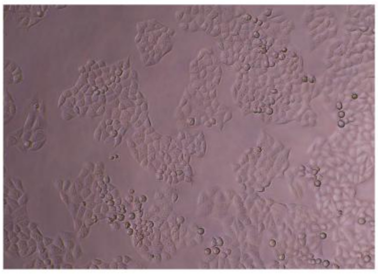

NL4-3 + Nevirapine

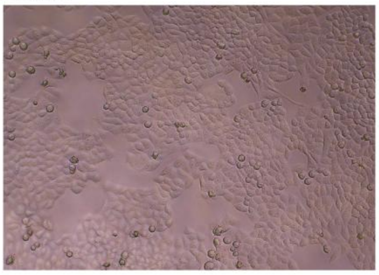

NL4-3 + ZCCHC3

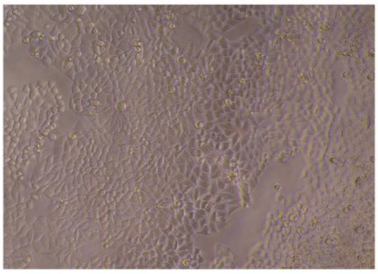

NL4-3 + empty

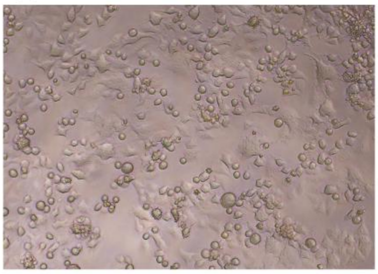

Figure S1B

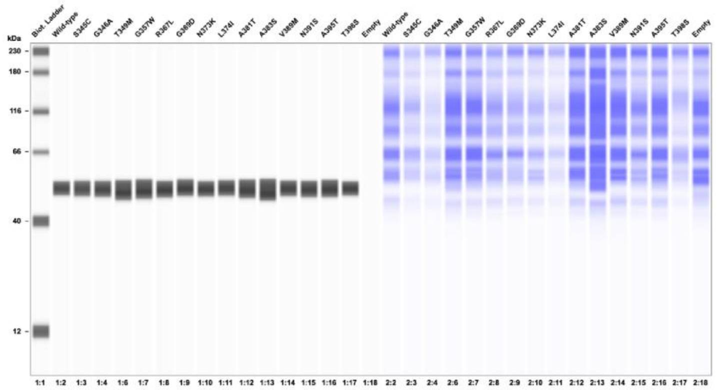
kDa
230
180
116
66
40
12
Biot. Ladder
Wild-type
S345C
G346A
T349M
G357W
R367L
G369D
N373K
L374I
A381T
A383S
V389M
N391S
A395T
T398S
Empty
Wild-type
S345C
G346A
T349M
G357W
R367L
G369D
N373K
L374I
A381T
A383S
V389M
N391S
A395T
T398S
Empty
1:1 1:2 1:3 1:4 1:6 1:7 1:8 1:9 1:10 1:11 1:12 1:13 1:14 1:15 1:16 1:17 1:18 2:2 2:3 2:4 2:6 2:7 2:8 2:9 2:10 2:11 2:12 2:13 2:14 2:15 2:16 2:17 2:18

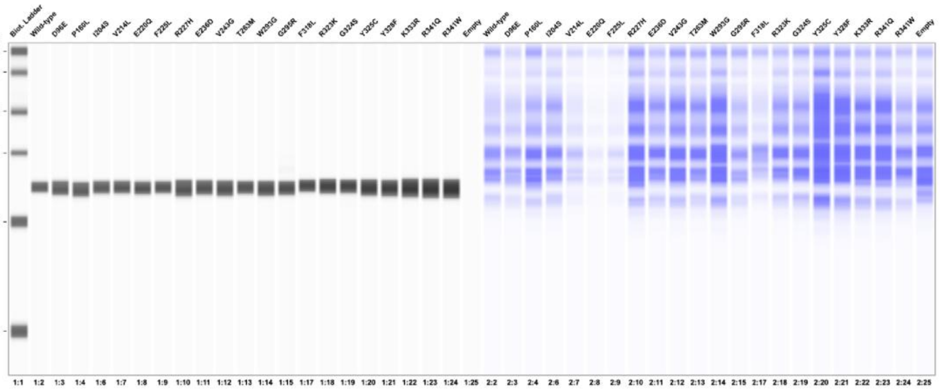
kDa
230
180
116
66
40
12
Biot. Ladder
Wild-type
D96E
P160L
I204S
V214L
E220Q
F225L
R227H
E236D
V243G
T263M
W293G
G295R
F318L
R323K
G324S
Y325C
Y328F
K333R
R341Q
R341W
Empty
Wild-type
D96E
P160L
I204S
V214L
E220Q
F225L
R227H
E236D
V243G
T263M
W293G
G295R
F318L
R323K
G324S
Y325C
Y328F
K333R
R341Q
R341W
Empty
1:1 1:2 1:3 1:4 1:6 1:7 1:8 1:9 1:10 1:11 1:12 1:13 1:14 1:15 1:17 1:18 1:19 1:20 1:21 1:22 1:23 1:24 1:25 2:2 2:3 2:4 2:6 2:7 2:8 2:9 2:10 2:11 2:12 2:13 2:14 2:15 2:17 2:18 2:19 2:20 2:21 2:22 2:23 2:24 2:25

Figure S1I

experiment 1

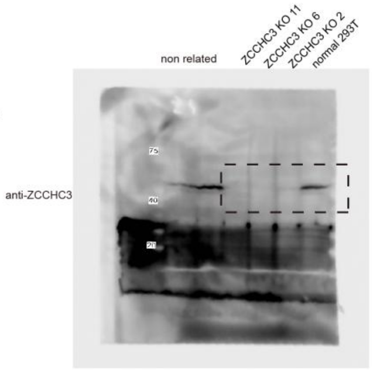

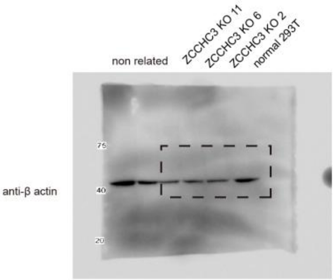

experiment 2 and 3

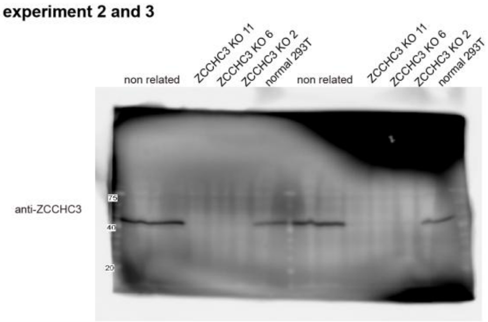

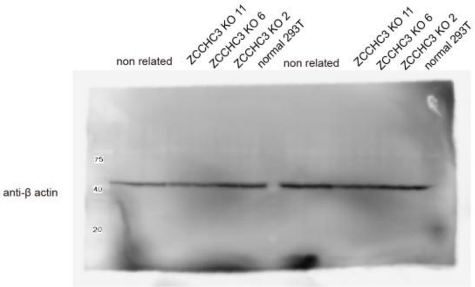

Figure 2B

TF2625 cell lysate (293T)

cell lysate

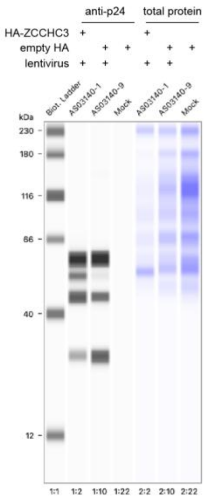

virion

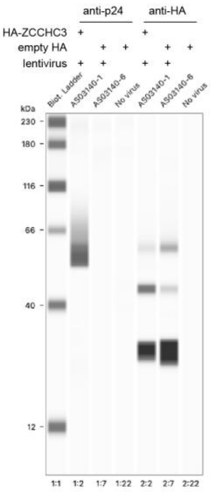

Supplement: Data S1. Raw images of western blots and microscopic images, related to Figure 1 [file mmc5.pdf]
