# Supplementary figures and images for: Host ZCCHC3 blocks HIV-1 infection and production through a dual mechanism
Source: iScience. 2024 Feb 5;27(3):109107. doi: 10.1016/j.isci.2024.109107 (PMC10879702; doi:10.1016/j.isci.2024.109107)

Data S2: Raw images of western blots and microscopic images, related to Figure 2.

Figure S2A

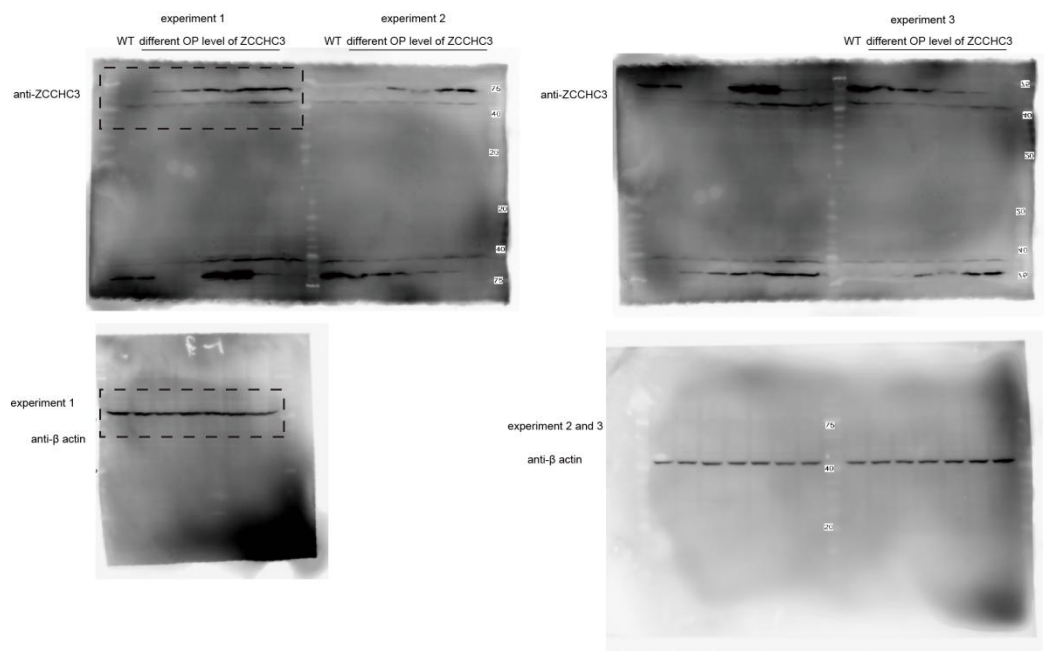

Supplement: Data S2. Raw images of western blots and microscopic images, related to Figure 2 [file mmc6.pdf]
