# Supplementary material for: Host ZCCHC3 blocks HIV-1 infection and production through a dual mechanism
Source: iScience. 2024 Feb 5;27(3):109107. doi: 10.1016/j.isci.2024.109107 (PMC10879702; doi:10.1016/j.isci.2024.109107)
Supplement: Data S3. Raw images of western blots and microscopic images, related to Figure 3 [file mmc7.pdf]

Data S3: Raw images of western blots and microscopic images, related to Figure 3.

Figure 3A and S3A

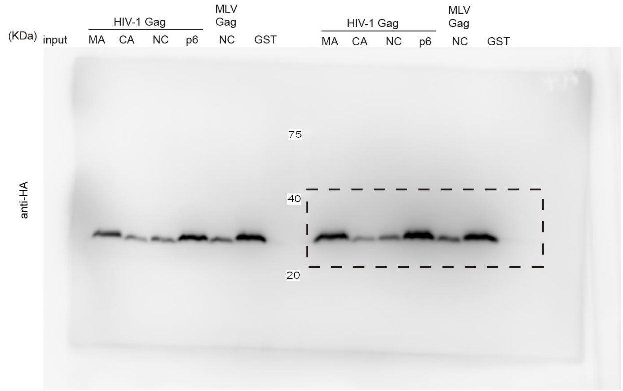

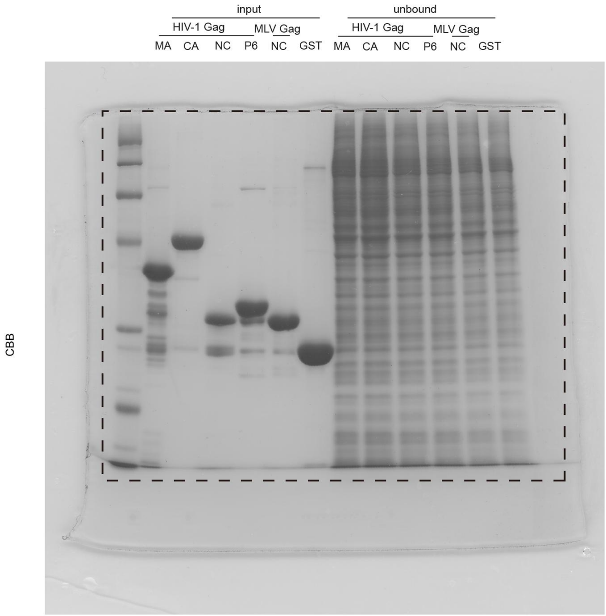

Figure 3B

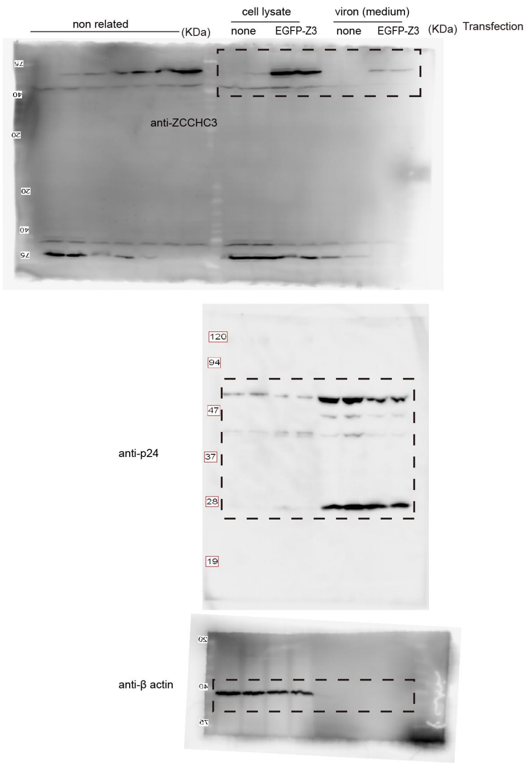
non related
(KDa)
cell lysate
none
EGFP-Z3
viron (medium)
none
EGFP-Z3
(KDa)
Transfection
anti-ZCCHC3
120
94
47
37
28
19
anti-p24
anti-β actin

Figure 3C and 4J

experiment 1

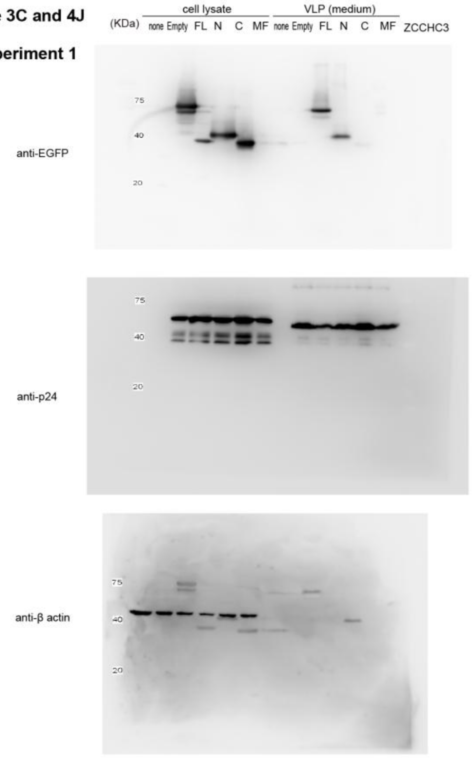

experiment 2

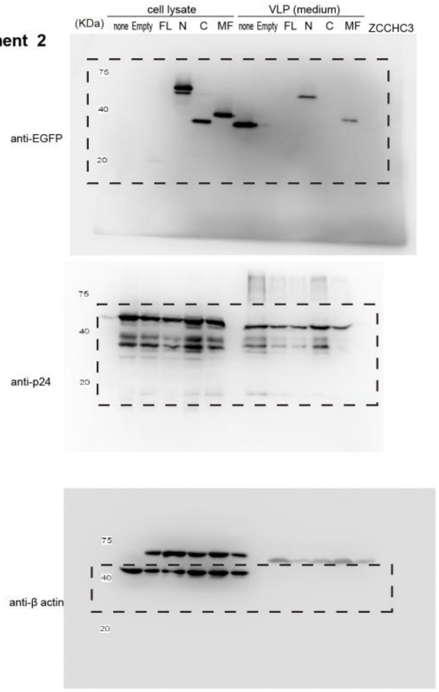

experiment 3

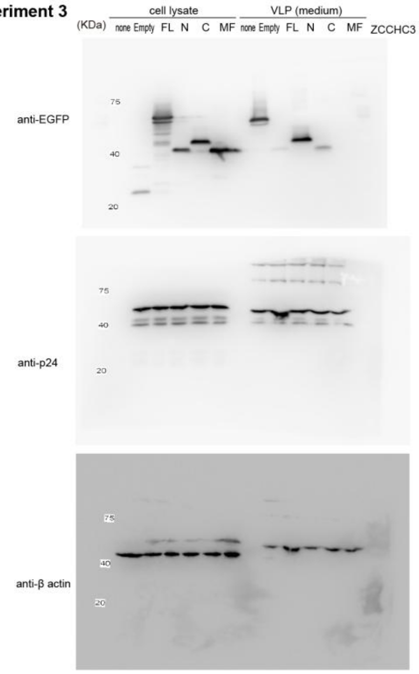

Figure 3D and S3B

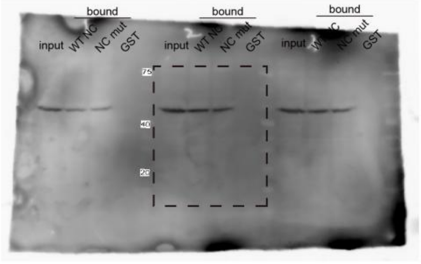

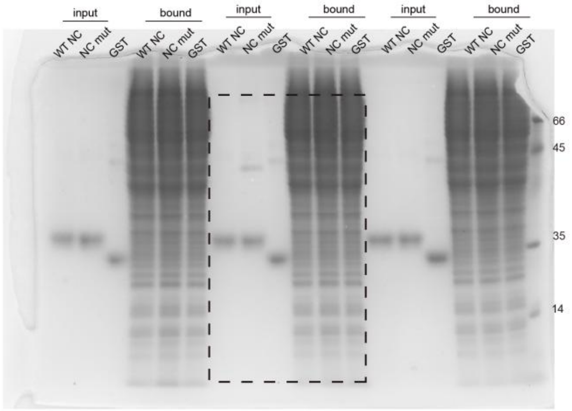

Figure S3H

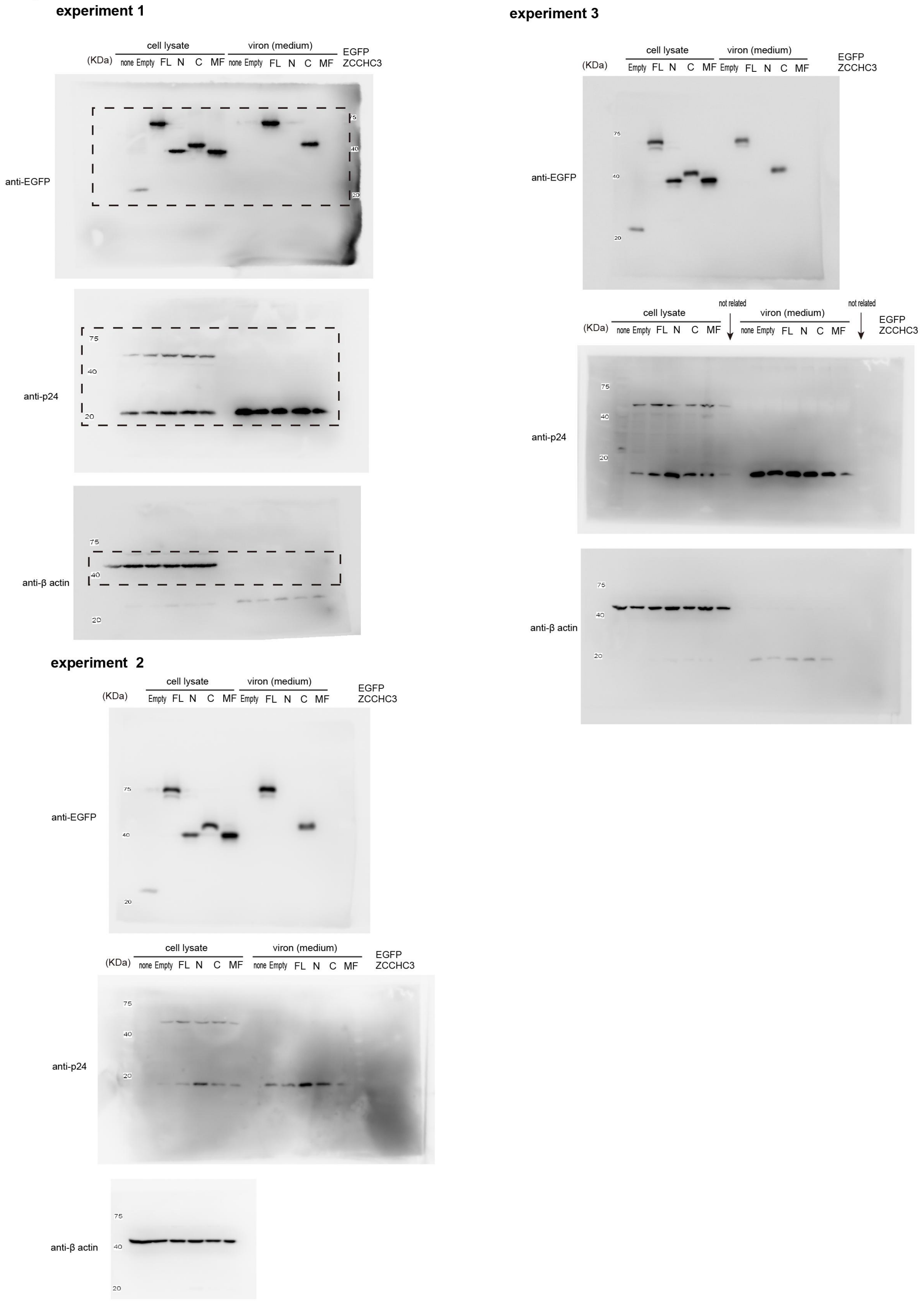
experiment 1
cell lysate
viron (medium)
(KDa)
none Empty FL N C MF none Empty FL N C MF
EGFP
ZCCHC3
anti-EGFP
anti-p24
anti-β actin
75
40
20
experiment 2
cell lysate
viron (medium)
(KDa)
Empty FL N C MF Empty FL N C MF
EGFP
ZCCHC3
anti-EGFP
none Empty FL N C MF none Empty FL N C MF
anti-p24
anti-β actin
experiment 3
cell lysate
viron (medium)
(KDa)
Empty FL N C MF Empty FL N C MF
EGFP
ZCCHC3
anti-EGFP
not related
none Empty FL N C MF none Empty FL N C MF
anti-p24
anti-β actin
