# Supplementary material for: Host ZCCHC3 blocks HIV-1 infection and production through a dual mechanism
Source: iScience. 2024 Feb 5;27(3):109107. doi: 10.1016/j.isci.2024.109107 (PMC10879702; doi:10.1016/j.isci.2024.109107)

Data S4: Raw images of western blots and microscopic images, related to Figure 4.

**CBB staining of Figure 4D**

**experiment 1**

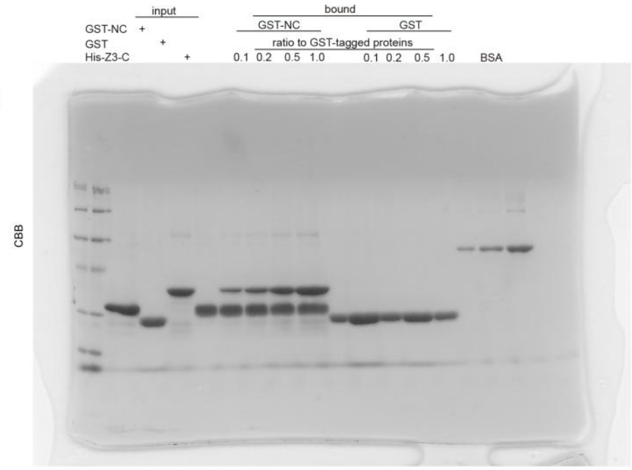


**experiment 2**

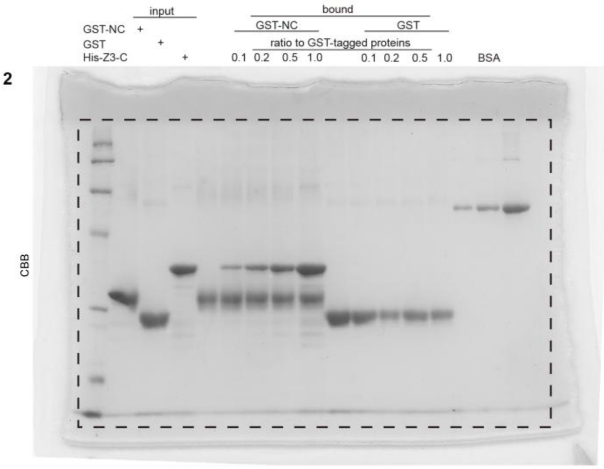


**experiment 3**

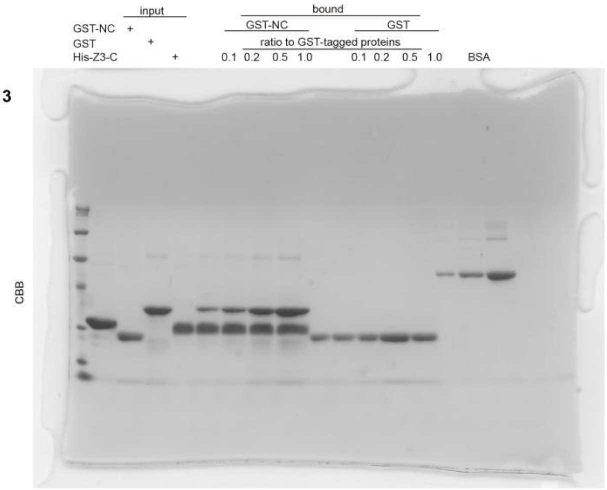

**CBB staining of Figure S4B**

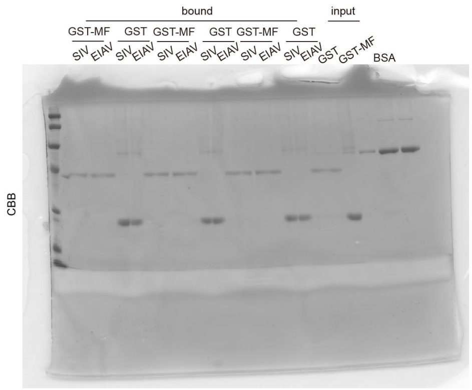


**Figure S4C**

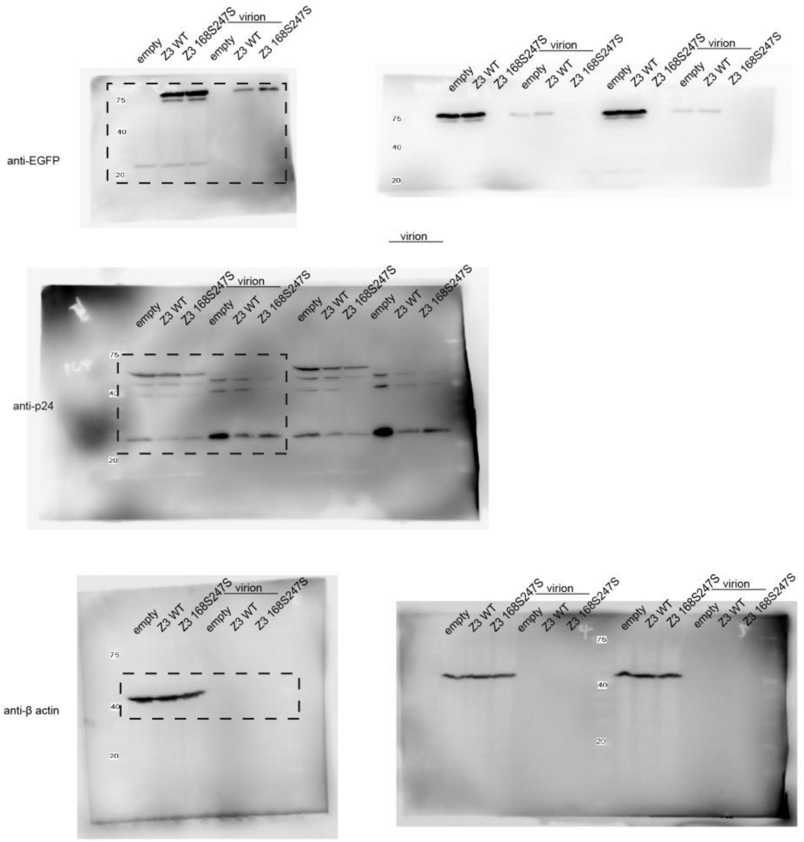

**Figure 4E and S3F**

**experiment 1**

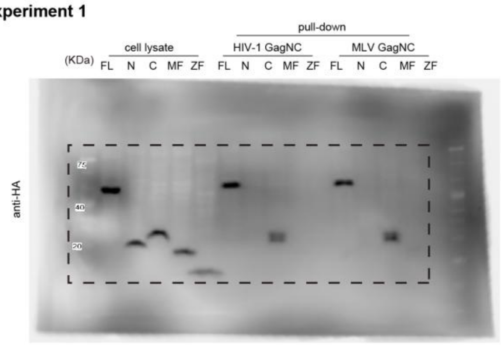


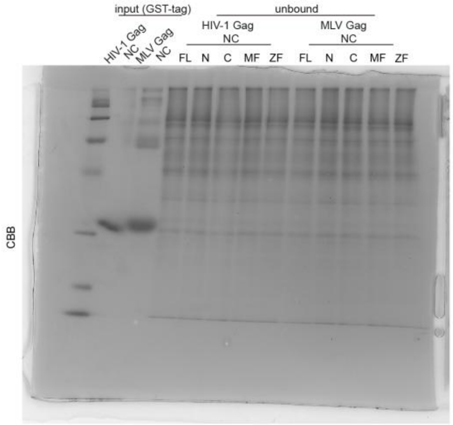


**experiment 2**

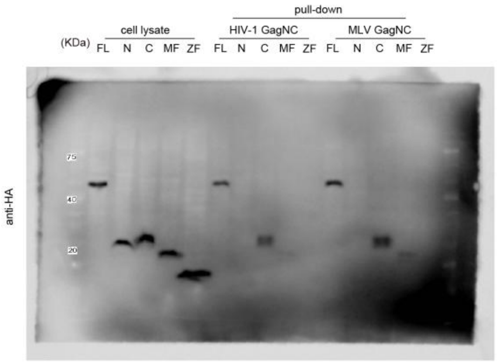


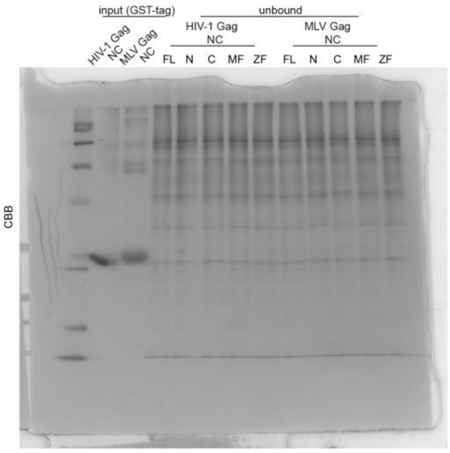


**experiment 3**

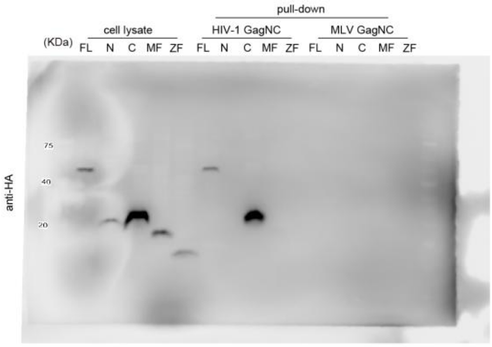


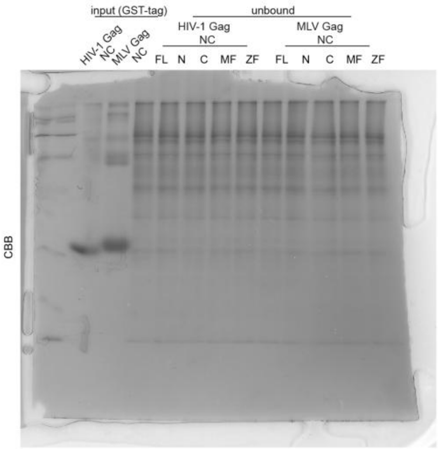

Supplement: Data S4. Raw images of western blots and microscopic images, related to Figure 4 [file mmc8.pdf]
