# Supplementary material for: Host ZCCHC3 blocks HIV-1 infection and production through a dual mechanism
Source: iScience. 2024 Feb 5;27(3):109107. doi: 10.1016/j.isci.2024.109107 (PMC10879702; doi:10.1016/j.isci.2024.109107)

Data S5: Raw images of western blots and microscopic images, related to Figure 5.

Figure 5B

CBB staining for RNA pulldown

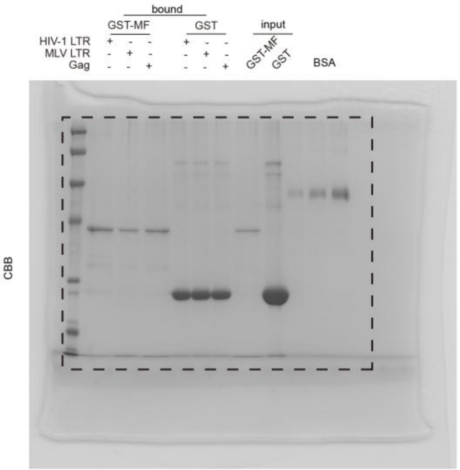

CBB staining for DNA pulldown

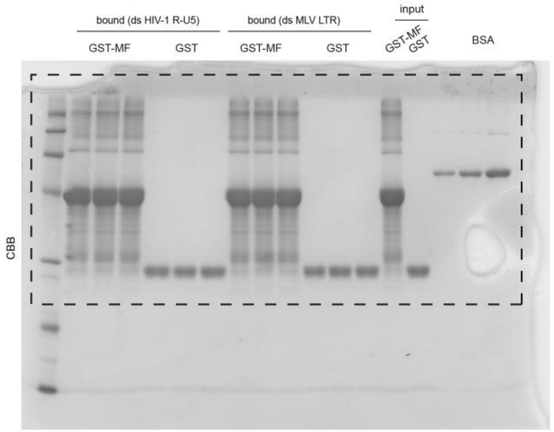

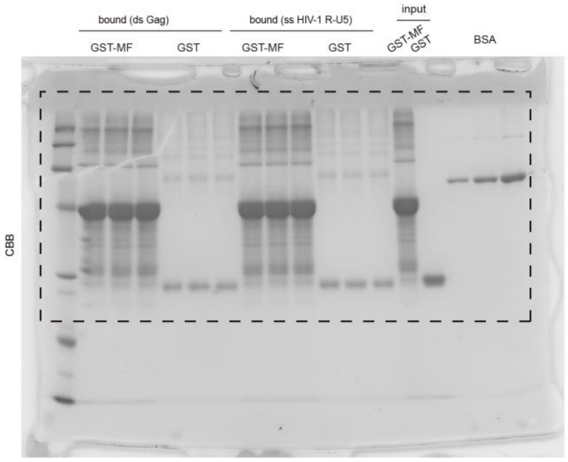

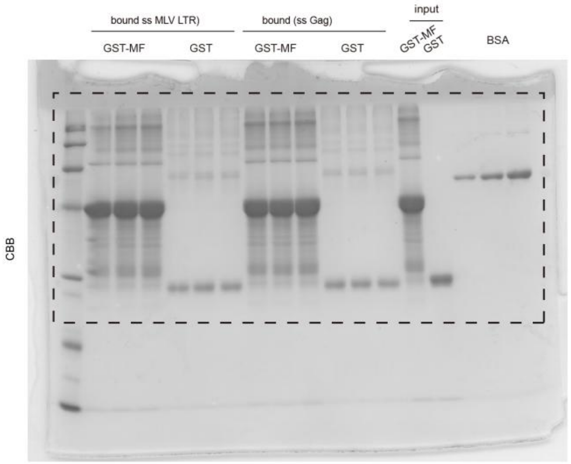

Figure 5C

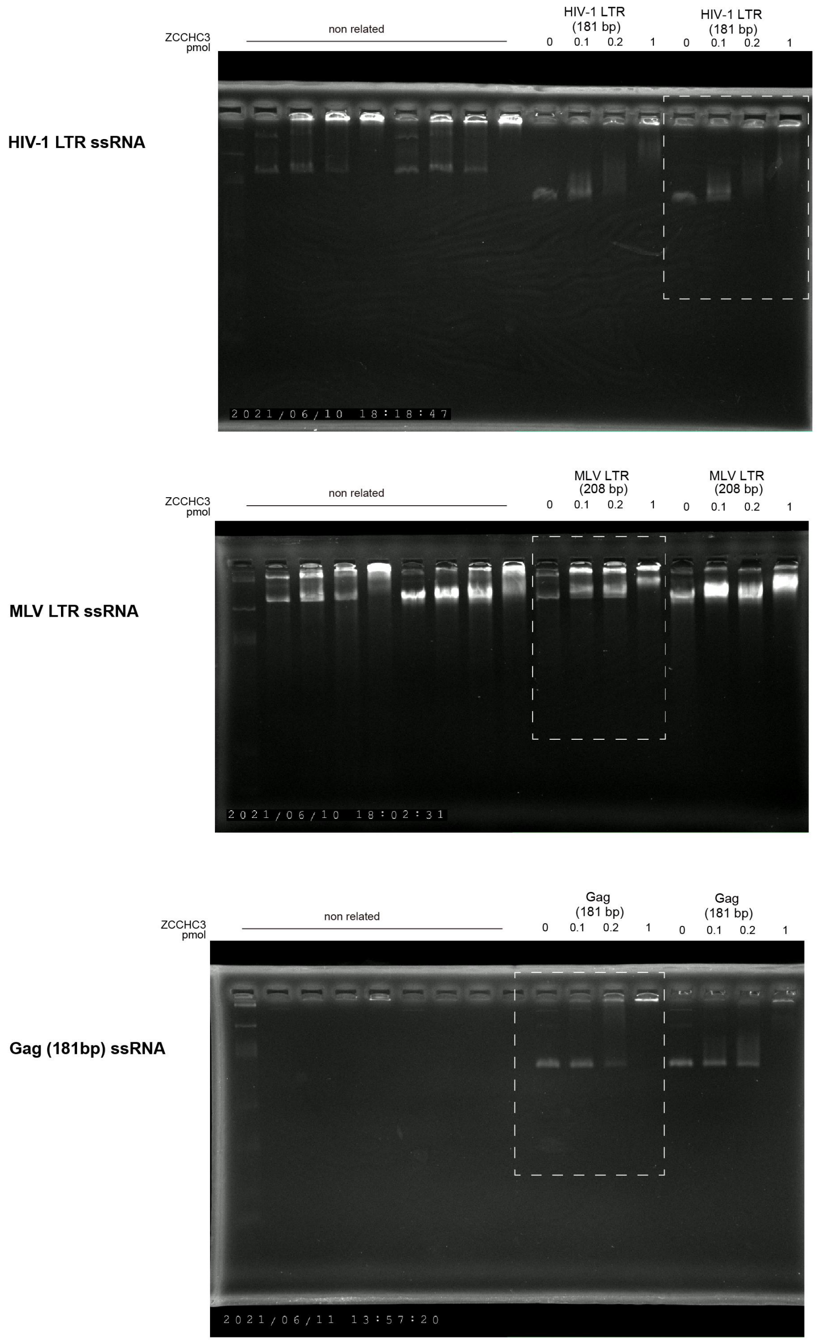
ZCCHC3 pmol
non related
HIV-1 LTR (181 bp)
0 0.1 0.2 1
HIV-1 LTR (181 bp)
0 0.1 0.2 1
HIV-1 LTR ssRNA
2021/06/10 18:18:47
ZCCHC3 pmol
non related
MLV LTR (208 bp)
0 0.1 0.2 1
MLV LTR (208 bp)
0 0.1 0.2 1
MLV LTR ssRNA
2021/06/10 18:02:31
ZCCHC3 pmol
non related
Gag (181 bp)
0 0.1 0.2 1
Gag (181 bp)
0 0.1 0.2 1
Gag (181bp) ssRNA
2021/06/11 13:57:20

Figure 5C

HIV-1 LTR ss and dsDNA

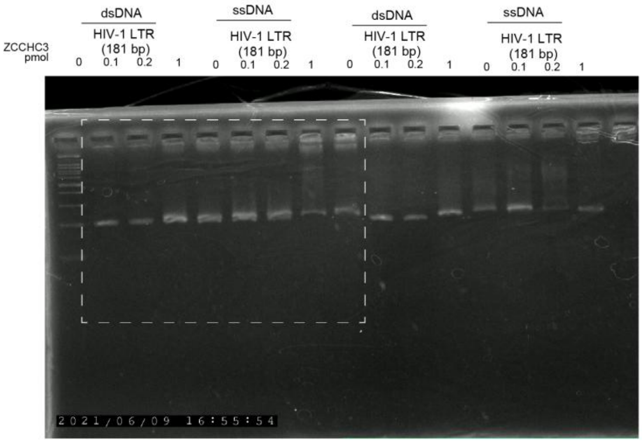

MLV LTR ssDNA and dsDNA

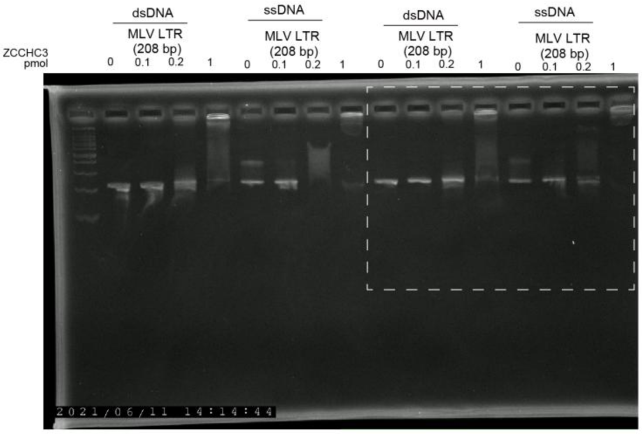

Gag (181bp) ssDNA and dsDNA

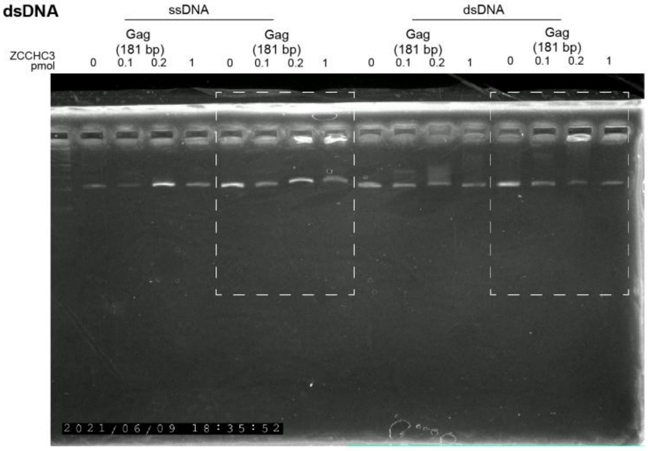

CBB staining of Figure 5D

experiment 1 and 2

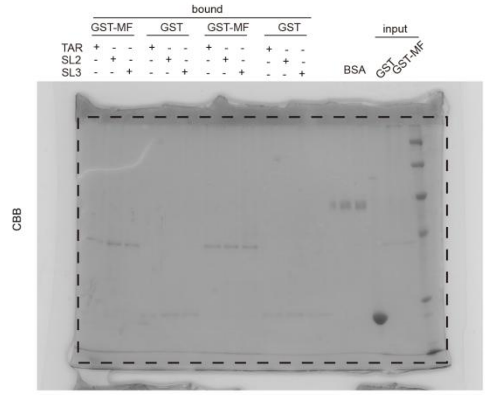

experiment 3 and 4

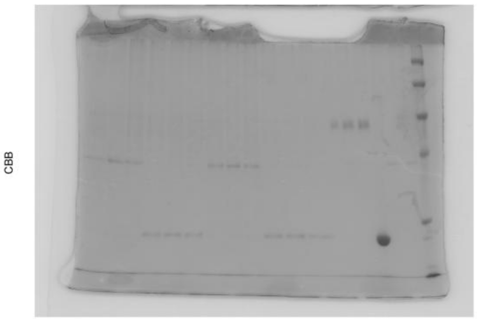

experiment: RRE

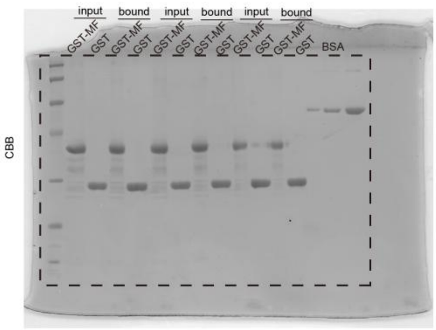

experiment: ZCCHC3 FL

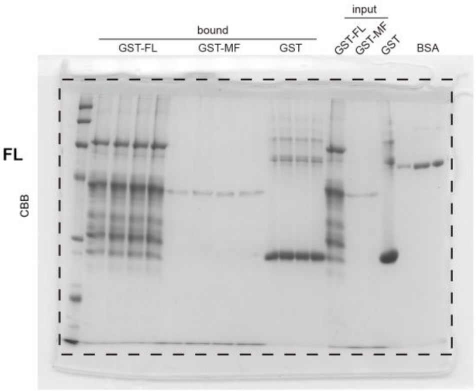

CBB staining of Figure 5F

input

WT R164S R168S R210S R247S R253S R289S K298S K329S 2X GST BSA

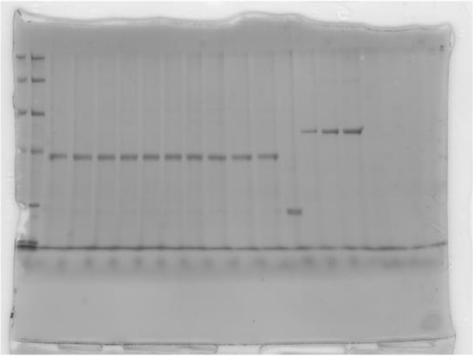

bound 1

WT R164S R168S R210S R247S R253S R289S K298S K329S 2X GST BSA

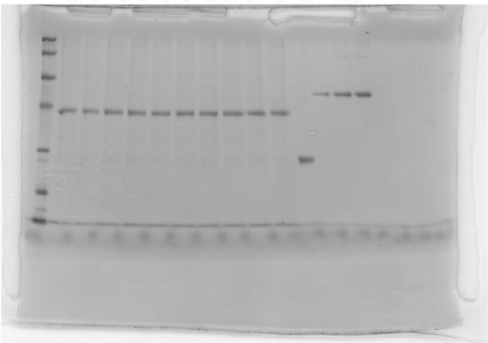

bound 2

WT R164S R168S R210S R247S R253S R289S K298S K329S 2X GST BSA

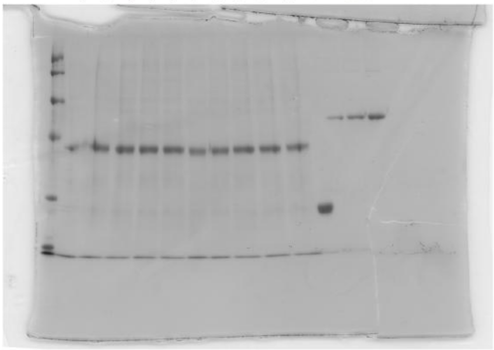

bound 3

WT R164S R168S R210S R247S R253S R289S K298S K329S 2X GST BSA

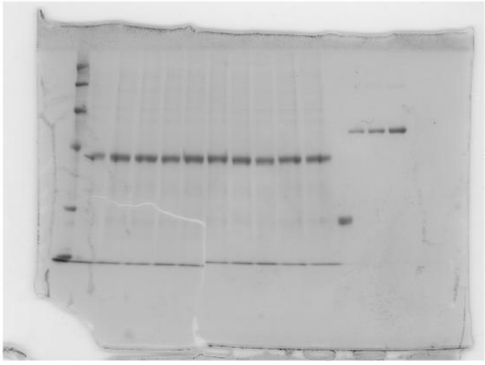

CBB staining of Figure 5F

experiment 1

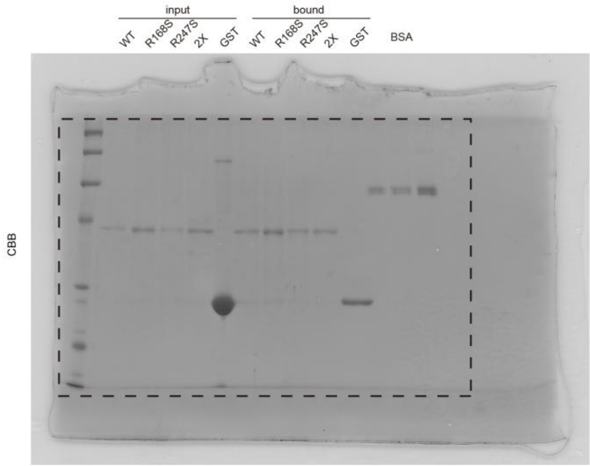

experiment 2

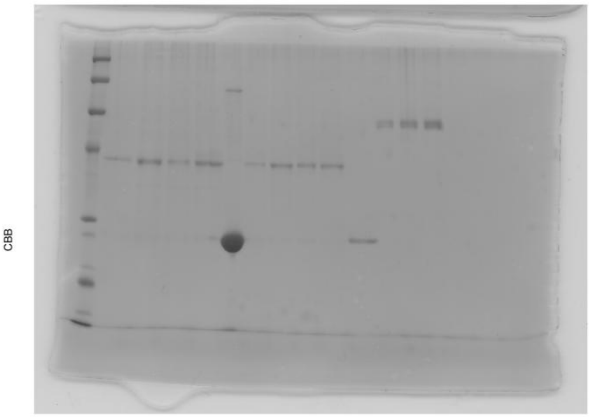

experiment 3

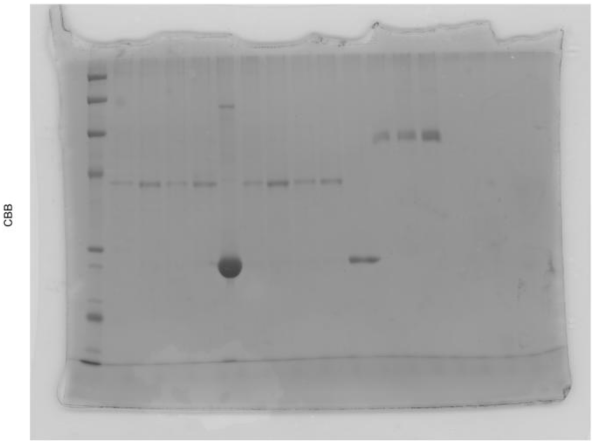

Supplement: Data S5. Raw images of western blots and microscopic images, related to Figure 5 [file mmc9.pdf]
